# Cancer IDO1‐Mediated Tryptophan–Kynurenine Metabolic Reprogramming to Drive Skeletal Muscle Atrophy and Cachexia Acceleration

**DOI:** 10.1002/jcsm.70295

**Published:** 2026-04-24

**Authors:** Leng Han, Lingjie Jing, Xinting Zhu, Ciqin Li, Lu Bai, Yang Fang, Yuxuan Zhou, Dingyuan Bai, Jin Lu, Yonglong Han, Cheng Guo, Shumin Zhou, Quanjun Yang

**Affiliations:** ^1^ Department of Pharmacy Shanghai Sixth People's Hospital Affiliated Shanghai Jiao Tong University School of Medicine Shanghai China; ^2^ Shanghai University of Traditional Chinese Medicine Shanghai China; ^3^ School of Biological and Biomedical Engineering Donghua University Shanghai China; ^4^ College of Food Science Shanghai Ocean University Shanghai China; ^5^ Institute of Microsurgery on Extremities Shanghai Sixth People's Hospital Affiliated Shanghai Jiao Tong University School of Medicine Shanghai China

**Keywords:** cancer cachexia, indoleamine 2,3‐dioxygenase 1, kynurenine, metabolic reprogramming, muscle atrophy, tryptophan

## Abstract

**Background:**

Cancer cachexia is a debilitating syndrome characterized by severe skeletal muscle wasting, which significantly impairs patient quality of life and survival. Indoleamine 2,3‐dioxygenase 1 (IDO1), a key enzyme in tryptophan (Trp) metabolism, is often upregulated in cancers, but its specific role in driving lung cancer–associated cachexia remains inadequately defined. This study investigated the mechanistic role of *Ido1* in cancer cachexia and evaluated the therapeutic potential of its inhibition.

**Methods:**

We established Lewis lung carcinoma (LLC) models in C57BL/6 mice using wild‐type, *Ido1*‐overexpressing (*Ido1*‐OE) and *Ido1*‐knockout (*Ido1*‐KO) cells. Muscle mass, tumour growth and metabolic changes were assessed in vivo. Transcriptomic and targeted metabolomic analyses were performed on muscle and serum samples. In vitro, we examined the effects of tumour‐conditioned media, the Trp metabolite kynurenine (Kyn) and Trp supplementation on C2C12 myotube atrophy. In vivo experiments verified the efficacy of the *Ido1* inhibitor palmatine hydrochloride (PAL). Molecular pathways were analysed via western blot and qPCR.

**Results:**

Compared to LLC mouse models, *Ido1*‐OE significantly exacerbated tumour growth and cachexia, leading to a significant decrease in lean body weight, gastrocnemius and tibialis anterior muscle weights (*p* < 0.01, *p <* 0.0001, *p* < 0.001). Gastrocnemius muscle fibre cross‐sectional area significantly decreased in the *Ido1*‐OE group (*p* < 0.0001). Transcriptomic analysis revealed that *Ido1‐*OE activated pro‐inflammatory and protein degradation pathways (upregulating MuRF1/Atrogin1, *p* < 0.05) while suppressing anabolic signalling pathways (oestrogen pathways, *p* < 0.01). Metabolomics analysis revealed unique metabolic signatures in *Ido1*‐OE mice: Trp depletion and Kyn accumulation. In vitro experiments demonstrated that *Ido1‐*OE enhanced LLC cell proliferation and migration capabilities (*p* < 0.0001, *p* < 0.0001). Tumour‐conditioned medium (TCM) derived from *Ido1*‐OE tumours significantly induced C2C12 myotube atrophy (*p* < 0.01). Similarly, direct treatment with Kyn led to dose‐dependent muscle fibre shrinkage, with significant atrophy observed at 30 μM (*p* < 0.01) and 100 μM (*p* < 0.0001). Notably, the myotube atrophy induced by Kyn was significantly reversed by the addition of supplemental Trp (*p* < 0.0001). Compared with the *Ido1*‐OE group, PAL treatment reduced gastrocnemius and tibialis anterior atrophy (*p* < 0.01; *p* < 0.05). Mechanistically, PAL inhibited the mRNA expression levels of MuRF1/Atrogin1 (*p* < 0.0001, *p* < 0.001), as well as their corresponding protein levels (*p* < 0.0001, *p* < 0.0001). Furthermore, PAL restored the phosphorylation level of mTOR (*p* < 0.001), as well as the mRNA expression of myosin heavy chain (*p* < 0.01).

**Conclusions:**

Our findings demonstrate that *Ido1* accelerates muscle atrophy and cancer cachexia by driving a metabolic reprogramming centred on the Trp–Kyn pathway. Pharmacological inhibition of *Ido1* with PAL effectively mitigates these effects, positioning *Ido1* as a promising therapeutic target for treating cancer cachexia.

## 1 Introduction

Cancer cachexia is a multifactorial and debilitating metabolic syndrome characterized by progressive and involuntary loss of skeletal muscle mass with or without adipose tissue wasting that cannot be fully reversed by conventional nutritional support [1, 2]. This disorder affects the majority of patients with advanced cancers, particularly occurring in 50%–80% of lung cancer patients [3]. Notably, cachexia is directly responsible for approximately 20% of all cancer‐related deaths [1]. The clinical sequelae of cachexia, including profound weakness, fatigue and functional impairment, severely diminish patients' quality of life and tolerance to anti‐cancer therapies [4]. Given its significant clinical impact, the urgent need for effective, precise and targeted interventions has driven extensive research into the underlying mechanisms and therapeutic concepts.

The pathogenesis of cancer cachexia is driven by complex interactions between the tumour and host, mediated by metabolic dysregulation, systemic inflammation and disrupted energy balance [5]. Cancer‐related metabolites are key contributors that activate metabolic and signalling pathways such as the ubiquitin‐proteasome system (UPS), which drives protein catabolism and muscle wasting [6]. The muscle‐specific E3 ubiquitin ligases, Muscle RING Finger 1 (MuRF1) and Muscle Atrophy F‐box (Atrogin‐1), have been reported to be associated with cancer cachexia and metabolic dysfunction [7]. Accompanying these shifts, the depletion of essential metabolic nutrients and accumulation of secondary metabolites contribute to disorders in metabolic sensing signals such as the mechanistic target of rapamycin (mTOR), thereby impairing protein anabolism [8].

Multiple serum metabolomic studies have revealed cancer cachexia in patients with Trp metabolism disorders [9]. Trp is an essential amino acid that is catabolized primarily via the Kyn pathway, a process initiated and rate‐limited by indoleamine 2,3‐dioxygenase 1 (IDO1) [10]. *IDO1* is overexpressed in multiple human cancers and is associated with reduced survival [11]. Its role in promoting an immunosuppressive tumour microenvironment by depleting Trp and accumulating Kyn and other immunosuppressive metabolites has been extensively studied [12]. Emerging evidence suggests that this metabolic pathway may be directly involved in driving muscle wasting [13]. Preclinical studies indicate that elevated Kyn levels can induce oxidative stress and muscle atrophy, whereas Trp depletion may impair protein synthesis [14]. Tumours overexpressing *IDO1* show local and circulatory inflammatory cytokine elevations, such as tumour necrosis factor‐alpha (TNF‐α) and interleukin‐6 (IL‐6) [15]. These inflammatory cytokines have also been reported to contribute to the inhibition of protein synthesis and trigger protein catabolic processes [16]. Based on these observations, we investigated the role of *Ido1‐*mediated Trp–Kyn metabolic reprogramming in lung cancer–associated cachexia pathogenesis.

In this study, we hypothesized that tumour‐derived *Ido1* exacerbates cancer cachexia by reprogramming the Trp metabolism, particularly the Trp–Kyn pathway, thereby activating muscle catabolism and suppressing anabolism. We employed a Lewis lung carcinoma (LCC) model with genetic manipulation of *Ido1* expression to systematically evaluate its impact on tumour growth, muscle wasting and global metabolic profiles. We further investigated the direct effects of the *Ido1* metabolite Kyn on C2C12 myotubes and evaluated the therapeutic potential of the *Ido1* inhibitor palmatine hydrochloride (PAL) [17]. Our findings establish *Ido1* as a critical mediator of cancer cachexia and provide a compelling rationale for its therapeutic targeting.

## 2 Materials and Methods

### 2.1 Reagents and Antibodies

IDO1 Rabbit Monoclonal Antibody (D8W5E, #51851), mTOR Rabbit Monoclonal Antibody (7C10, #2983), Phospho‐mTOR (Ser2448) Rabbit Monoclonal Antibody (D9C2, #5536), 70 kDa ribosomal protein S6 kinase (p70S6K) Rabbit Monoclonal Antibody (E8K6T, #34475), Phospho‐p70 S6 Kinase (Thr389) Rabbit Monoclonal Antibody (108D2, #9234), eIF4E‐binding protein 1 (4E‐BP1) Rabbit Monoclonal Antibody (53H11, #9644) and Phospho‐4E‐BP1 (Thr37/46) Rabbit Monoclonal Antibody (236B4, #2855) were purchased from Cell Signaling Technology (MA, USA). Myosin Heavy Chain (MYH) Antibody (B‐5, SC‐376157) was purchased from Santa Cruz Biotechnology (CA, USA). Tripartite motif‐containing 63 (TRIM63) Polyclonal antibody (55 456‐1‐AP), MYH1 Monoclonal antibody (1G10H9, 67299‐1‐Ig) and MYH7‐specific Polyclonal antibody (22280‐1‐AP) were purchased from Proteintech (Wuhan, China). Anti‐Fbx32 antibody [EPR9148(2), ab168372] was purchased from Abcam (MA, USA). ABflo 488‐conjugated Goat anti‐Mouse IgG (H + L) (AS037) and Cy3‐conjugated Goat anti‐Rabbit IgG (H + L) (AS007) were purchased from ABclonal Biotechnology (Wuhan, China). HRP Conjugated Anti‐GAPDH Antibody (SA30‐01, HA721131) was purchased from Huaan Biotechnology (Hangzhou, China). Palmatine hydrochloride (10605‐02‐4, A0031) was purchased from Chengdu Must Biotechnology (Chengdu, China). l‐Kynurenine (2922‐83‐0, HY‐104026) and l‐tryptophan (73‐22‐3, HY‐N0623) were purchased from MedChemExpress (NJ, USA).

### 2.2 Cell Culture and Differentiation

The mouse C2C12 myoblast cell line, Lewis lung carcinoma cell line (LLC) and HEK293 T‐cell line (Cell Bank of Shanghai Branch, Chinese Academy of Sciences, Shanghai, China) were cultured in DMEM (Gibco, MA, USA) supplemented with 10% fetal bovine serum (FBS, Bioagrio, Shanghai, China) and 1% penicillin and streptomycin. To differentiate the C2C12 myoblasts into myotubes, the medium was changed to differentiation medium (DMEM with 2% horse serum) when cell confluence reached 70%. The differentiation medium was replaced with fresh medium every day, and myotubes were observed after 3 days. All the cells were incubated at 37°C with 5% CO_2_ in an air atmosphere.

### 2.3 The Conditioned Medium Collection

LLC (2 × 10^6^ cells) were seeded in 10‐mm dishes and grown to 90% confluence. The medium was changed to DMEM containing 2% horse serum for 36 h, and the cell supernatant was collected and centrifuged at 450 g for 5 min. The supernatant was referred to as tumour‐conditioned medium (TCM).

### 2.4 Immunofluorescence

C2C12 myotubes were fixed in 4% paraformaldehyde and permeabilized with 0.5% Triton X‐100 at room temperature, followed by incubation with anti‐MYH antibody overnight at 4°C. After incubation with secondary antibodies, C2C12 myotubes were stained with 10 μg/mL 4′,6‐diamidino‐2‐phenylindole (DAPI, Servicebio Technology, Wuhan, China) and photographed using Leica Application Suite X (Leica, HE, Germany).

### 2.5 *Ido1* KO and OE in LLC Using a Lentivirus

To produce *Ido1*‐overexpression (*Ido1‐*OE) LLC cells, the cDNA of *Ido1* (NM_008324.3) was amplified by PCR. The plasmid pLV‐EF1α‐FLAG‐IRES‐Puro (Plasmid #85132 from Addgene) was digested with EcoR1 and BamH1. The lentiviral plasmid pLV‐CMV‐*Ido1*‐EF1α‐GFP‐Puro was produced by Gibson assembly. The resulting plasmid was sequenced to verify the correct insertion of the insert. To produce *Ido1*‐knockout (*Ido1‐*KO) LLC cells, the three sgRNAs were designed by CRISPick (https://portals.broadinstitute.org/gppx/crispick/public), and three sgRNAs were selected. The sgRNA sequences used were sg1: 5′‐CAAGTGAAACTTGCTGACTT‐3′, sg2: 5′‐CTTGGCCTGGTTCTGTCAGA‐3′ and sg3: 5′‐CATGGCTAAAATTACTTCTG‐3′. The three sgRNAs were synthesized and cloned into the plasmid pLV3‐U6‐MCS‐EF1α‐Cas9‐FLAG‐IRES‐Puro. The lentiviruses were produced in low‐passage HEK293 T cells (Cell Bank of Shanghai Branch, Chinese Academy of Sciences, Shanghai, China). Approximately 2 h before transfection, the complete DMEM medium was replaced by 13 mL of pre‐warmed Opti‐MEM medium (Invitrogen Inc., CA, USA). For each 15‐cm plate, 450 μL of Opti‐MEM was mixed with 20 μg lentiviral transfer plasmid, 15 μg psPAX2 packaging plasmid, 10 μg pMD2.G envelope plasmid and 130 μL polyethyleneimine. The mixture was incubated for 15 min at room temperature and added to the cells. After 6–12 h, the Opti‐MEM medium was replaced with 20 mL of pre‐warmed complete DMEM medium. Viral supernatant was collected at 48‐ and 72‐h post‐transfection. Cell debris was removed by centrifugation (5 min at 2000 rpm). Lentiviruses were concentrated using AmiconUltra 100 kD ultrafiltration centrifuge tube (Millipore, HE, Germany) and stored at −80°C. To generate *Ido1*‐OE LLC cells, 0.2 mL of concentrated lentivirus was added to low‐passage LLC cells at 5 × 10^4^ cells per well in 24‐well plates. The myoblasts were cultured for 16 h. The *Ido1*‐OE cells were selected with 1 μg/mL antibiotics for 7 days.

### 2.6 Animal Experimentation

All animal care and experiments were conducted according to the guidelines of the China Animal Welfare Legislation and were approved by the Ethics on Animal Care and Treatment Committee of Shanghai Jiao Tong University (No.: DWSY2023‐0174). Healthy male C57BL/6J mice (6–8 weeks old, 20–25 g) were purchased from the Shanghai Cyper‐BK Experimental Animal Co. Ltd. (Shanghai, China). The mice were housed under a 12:12 light–dark cycle at a controlled temperature of 21°C–23°C, designated as specific pathogen‐free (SPF) and provided standard rodent chow and water ad libitum. A mouse model of cancer cachexia was established as previously described [18]. The specific procedure was as follows: C57BL/6J mice were randomly divided into five groups: negative control (NC) LLC, *Ido1‐*OE, *Ido1‐*KO and *Ido1‐*OE with 15 mg/kg palmatine hydrochloride (PAL) (five mice per group). Each group received a subcutaneous injection of 1 × 10^6^ cells in 100 μL. Starting on the day after tumour inoculation, the mice in each group received daily injections of 15 mg/kg/day PAL (diluted in PBS) or PBS as a control. The optimal PAL dose was determined through a preliminary dose‐escalation pilot study, in which two dosages (15 and 30 mg/kg) were compared for their efficacy. As 15 mg/kg PAL achieved a significant restoration of gastrocnemius and tibialis anterior muscle weight without additional benefit observed at the higher 30 mg/kg dose, it was selected as the final experimental dosage. CCK‐8 assays were performed to evaluate the direct effect of PAL on LLC cell proliferation, and the results indicated that PAL did not exhibit significant cytotoxicity. This dose also aligns with pharmacological concentrations reported in previous studies [S1, 19]. Body and tumour weights were measured daily. Tumour weight (g) was calculated using the formula: 0.52 g/mm^3^ × tumour length (mm) × tumour width (mm)^2^/1000. Mice were euthanized when they exhibited significant weight loss and tumour weight did not exceed 10% of body weight. After euthanasia, tumour, plasma, gastrocnemius muscle and tibialis anterior muscle tissues were rapidly collected to confirm cachexia symptoms. Tumour, plasma and muscle samples were immediately snap‐frozen in liquid nitrogen and subsequently stored at −80°C for downstream transcriptomic sequencing, targeted metabolomic and various molecular assays. Concurrently, a segment of the gastrocnemius muscle and tibialis anterior muscle were excised and immersed in 4% paraformaldehyde for fixation to facilitate subsequent pathological and histological examinations, including muscle fibre cross‐sectional area (CSA) analysis.

### 2.7 Histological Examination

Gastrocnemius muscles were fixed with 4% paraformaldehyde and embedded in paraffin. Myofibre sizes were measured in sections stained with haematoxylin and eosin (H&E) and analysed using ImageJ software (Version 1.54g, NIH, USA). Immunofluorescence staining was performed to detect MYH1 and MYH7 expression.

### 2.8 Western Blot Analysis

The muscle tissues, tumour tissues and C2C12 myotubes were harvested and lysed using RIPA buffer containing Protease Inhibitor and Phosphatase Inhibitor Cocktail (Servicebio Technology, Wuhan, China). Equal amounts of protein samples (20–40 μg) were separated on SDS–PAGE gels and transferred to polyvinylidene fluoride membranes. The membranes were blocked with 5% fat‐free milk or BSA in Tris‐buffered saline with Tween 20 (TBST) and incubated with primary antibodies overnight at 4°C. The membranes were then incubated with secondary antibodies at room temperature for 2 h and visualized using an Odyssey system (LICOR, Lincoln, Nebraska). The density of each protein band was quantified using the ImageJ software (Version 1.54g, NIH, USA).

### 2.9 Quantitative PCR (qPCR)

Total RNA was isolated from C2C12 myotubes, muscle samples and tumour samples using TRIzol reagent (NCM Biotech, Suzhou, China), and 1 μg of total RNA was reverse‐transcribed into cDNA using Primescript II RT Master Mix Kits (Takara, Otsu, Japan) according to the manufacturer's protocol. Samples were diluted 1/5 and used to evaluate the mRNA expression of *Ido1*, *Murf1*, *Atrogin‐1*, *Myh*, *Myog* and *Myod*. GAPDH mRNA expression was used as an endogenous control, and relative expression was calculated using the ΔΔCt method. Primer sequences are listed in Table S1.

### 2.10 mRNA‐Seq and Transcriptomics Analysis

Total RNA was extracted from each sample using a standard TRIzol‐based protocol. RNA integrity was assessed using an Agilent Bioanalyzer (RIN > 7.0), and 1 μg of high‐quality total RNA per sample was used for mRNA library preparation. Poly(A) + mRNA was enriched from 1 μg total RNA using oligo (dT) magnetic beads. The mRNA was chemically fragmented, followed by first‐strand cDNA synthesis using reverse transcriptase and random hexamer primers and second‐strand cDNA synthesis incorporating dUTP to preserve strand orientation. The double‐stranded cDNA was end‐repaired, A‐tailed and ligated to indexed Illumina sequencing adapters. Libraries were PCR amplified using barcoded primers for multiplexing. Library amplification was performed with 11 PCR cycles using KAPA HiFi HotStart ReadyMix: 98°C for 30 s, followed by 11 cycles of 98°C for 10 s, 65°C for 75 s and a final extension at 72°C for 5 min. The adaptor‐ligated library was then purified and sent for quality assessment. If the library was of good quality, it was sequenced using the NovaSeq system (Illumina Inc., San Diego, CA, USA). Each sample was analysed in triplicate. Raw FASTQ reads were quality‐controlled using FastQC and trimmed using Trimmomatic. Clean reads were aligned to the mouse reference genome GRCm39 (Ensembl release 112) using HISAT2. Transcript assembly and abundance estimation were performed using StringTie software. Gene‐level counts were generated using the feature counts. Differential expression analysis was conducted using DESeq2, with genes considered differentially expressed if |log_2_(fold change)| > 0.263 and the adjusted *p*‐value < 0.05. Functional enrichment analysis (GO and KEGG) was performed using clusterProfiler. Gene Set Enrichment Analysis (GSEA) was carried out using the pre‐ranked mode in GSEA v4.0 (Broad Institute), ranking genes by log fold change.

### 2.11 Metabolomics and Data Analysis

Serum samples were collected from mice at the end of the experiment and stored at −80°C until analysis. Targeted metabolomic profiling was performed using ultra‐high‐performance liquid chromatography coupled with tandem mass spectrometry (UHPLC‐MS/MS; Thermo Fisher Scientific) following protein precipitation with cold methanol. Metabolites were separated on a C18 reverse‐phase column under gradient elution and detected in multiple reaction monitoring (MRM) mode. Quantification was performed by using calibration curves generated from authentic standards. Data acquisition and peak integration were conducted using the Xcalibur software, and metabolite identities were confirmed based on retention time and fragmentation patterns. Quality control samples were included throughout the run to ensure analytical reproducibility. Normalized metabolite intensities were log‐transformed and subjected to principal component analysis (PCA), hierarchical clustering and correlation analysis using MetaboAnalyst 5.0 (https://www.metaboanalyst.ca). Differential metabolites were identified based on fold changes and adjusted *p*‐values (*p* < 0.05), and pathway enrichment was performed using the KEGG and HMDB databases.

### 2.12 Statistical Analysis

Statistical analysis was conducted using the GraphPad Prism 8.0.2 software (Version 8.0.2, GraphPad Software Inc., USA). The *p*‐values are denoted with asterisks as follows: not significant (ns); *p < 0.05; **p < 0.01; ***p < 0.001; ****p < 0.0001. Data are presented as mean ± standard deviation (SD). At least three independent biological replicates were performed, and individual data points are shown in all graphs. Data were not excluded from the analysis. The two groups were compared using an unpaired two‐tailed Student's *t*‐test. For multiple independent groups, one‐way or two‐way analysis of variance (ANOVA) with Tukey's post hoc multiple comparisons test or Sidak's multiple comparisons test was used. Relative expression was determined by comparing the treatment values with the control values after normalization to the control values. Data that did not pass the variance test were compared using nonparametric two‐tailed Mann–Whitney rank‐sum tests or ANOVA on rank tests. Table S2 included raw dataset for Figure S1A. Table S3 included all raw datasets for statistical analysis and raw western blot images.

## 3 Results

### 3.1 *Ido1*‐OE Exacerbated Cancer Cachexia in the Murine LLC Model

**FIGURE 1:**
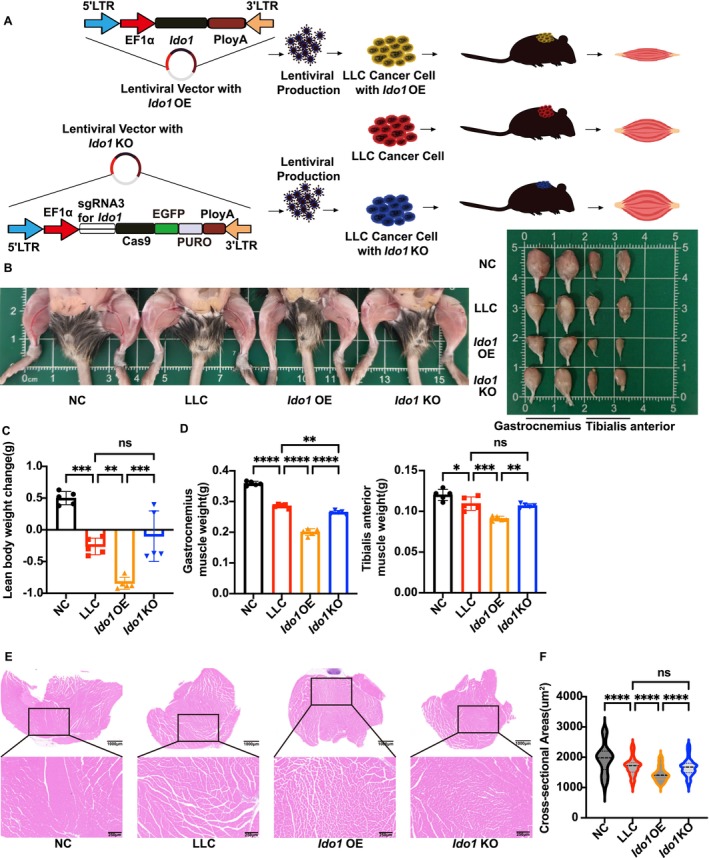
*Ido1‐*OE exacerbated cancer cachexia in the murine LLC model. (A) The diagram illustrates the construction of *Ido1‐*OE and *Ido1‐*KO in LLC cells and the effect on skeletal muscle in cancer cachexia mice. (B) The typical pictures of the hindlimb muscle, gastrocnemius and anterior tibial muscle from each group of mice. (C) The lean body weight changes of each group of mice. One‐way ANOVA with Sidak's multiple comparisons test was used for statistical analysis. The *p*‐values are denoted with asterisks as follows: not significant (ns); *p < 0.05; **p < 0.01; ***p < 0.001; ****p < 0.0001. (D) The gastrocnemius and anterior tibial muscle weights in each group of mice. One‐way ANOVA with Sidak's multiple comparisons test was used for statistical analysis. The *p*‐values are denoted with asterisks as follows: not significant (ns); *p < 0.05; **p < 0.01; ***p < 0.001; ****p < 0.0001. (E) The typical pictures of cross‐sectional H&E‐stained histopathological images of the gastrocnemius muscle from each group of mice. (F) The CSA of each group of mice. One‐way ANOVA with Sidak's multiple comparisons test was used for statistical analysis. The *p*‐values are denoted with asterisks as follows: not significant (ns); *p < 0.05; **p < 0.01; ***p < 0.001; ****p < 0.0001.

*IDO1* was overexpressed in multiple forms of lung cancer (Figure S1A). Accordingly, we analysed data from the TCGA database, which included 288 patients with *IDO1* alterations and 3421 unaltered individuals in a Kaplan–Meier survival analysis. The Logrank test yielded a *p*‐value of 0.301 (Figure S1B). Despite the lack of statistical significance, patients with *IDO1* alterations tended to exhibit poorer survival outcomes. In this study, we successfully established LLC cell lines with either overexpression or knockout of *Ido1* using lentiviral transduction and CRISPR‐Cas9 gene editing technologies (Figure S1C). The efficiency of these modifications was rigorously validated using both western blot and RT‐qPCR. The *Ido1*‐OE cells exhibited a dramatic increase in *Ido1* expression at both the mRNA and protein levels compared to control cells. For the knockout cell lines, three independent sgRNAs were screened. Quantitative analysis revealed that whereas all sgRNAs reduced *Ido1* levels, sg3 achieved the highest knockout efficiency, resulting in the most significant depletion of IDO1 protein (Figures S1D and S1E). Consequently, the sg3‐targeted LLC line was selected for all subsequent experiments due to its optimal efficiency and stability. These engineered and wild‐type LLC cells were subcutaneously implanted into C57BL/6 mice to generate a cancer cachexia model and to evaluate the role of *Ido1* in tumour‐induced muscle wasting (Figure 1A). C57 mice were randomly divided into the NC LLC, *Ido1‐*OE and *Ido1‐*KO groups (*n* = 5). By Day 6 post‐injection, palpable tumours were observed in all tumour‐bearing mice, and by Day 16, the animals were sacrificed for endpoint analysis. Gross examination of the hind limb and muscle tissues revealed marked differences between groups (Figure 1B). Tumour weight progressively increased over time in all groups, with *Ido1‐*OE significantly accelerating tumour growth, whereas *Ido1‐*KO markedly suppressed tumour growth (Figure S1F). Lean body weight change, calculated as the difference between final and initial body weights, showed a pronounced decline in the *Ido1‐*OE group compared to that in the LLC group (*p* < 0.01), whereas *Ido1‐*KO mitigated this loss (Figure 1C). Further analysis of skeletal muscle weight demonstrated that both gastrocnemius (*p* < 0.0001) and tibialis anterior (*p* < 0.001) muscles were significantly reduced in the *Ido1‐*OE group (Figure 1D). Histological assessment of the gastrocnemius muscle revealed that *Ido1‐*OE led to a substantial reduction in CSA, indicative of muscle atrophy (*p* < 0.0001) (Figure 1E,F). In contrast, *Ido1‐*KO alleviated this effect by maintaining larger fibre diameters. Collectively, these findings demonstrated that *Ido1* not only promotes tumour progression but also exacerbates cancer‐associated cachexia by inducing skeletal muscle wasting in tumour‐bearing C57BL/6 mice.

### 3.2 *Ido1* Remodelled the Skeletal Muscle Transcriptome by Suppressing Anabolic and Activating Catabolic Pathways

**FIGURE 2:**
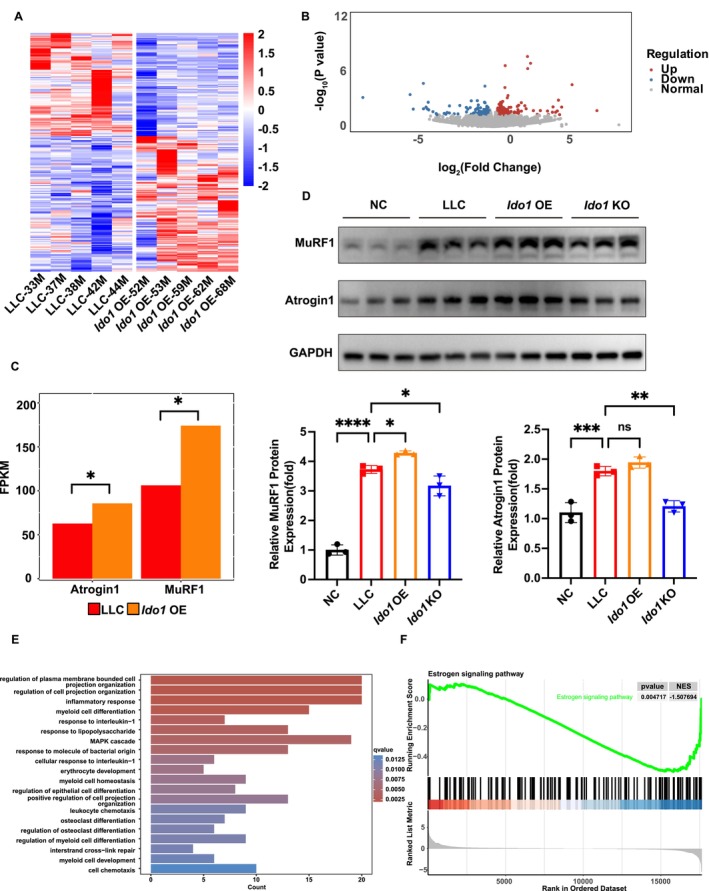
*Ido1* remodelled the skeletal muscle transcriptome by suppressing anabolic and activating catabolic pathways. (A) Heatmap of transcriptomic profiles from gastrocnemius muscle tissues, showing clear separation between the LLC and *Ido1‐*OE groups. (B) Volcano plot of DEGs between *Ido1‐*OE and LLC groups. Red dots represent upregulated genes (133), and blue dots represent downregulated genes (101), under the threshold of |log_2_FC| > 0.263 and *p* < 0.05. (C) Expression levels (FPKM) of the muscle atrophy marker genes MuRF1 and Atrogin1 from the transcriptomic data of LLC and *Ido1‐*OE groups. (D) Western blot analysis and quantification of MuRF1 and Atrogin1 protein expression in each group. Data are presented as fold change relative to NC. One‐way ANOVA with Sidak's multiple comparisons test was used for statistical analysis. The *p*‐values are denoted with asterisks as follows: not significant (ns); *p < 0.05; **p < 0.01; ***p < 0.001; ****p < 0.0001. (E) GO enrichment analysis of biological processes for DEGs in the *Ido1‐*OE group. The top enriched terms are associated with inflammatory and immune responses. Bar length indicates the number of genes; colour represents the *q*‐value. (F) GSEA plot shows significant suppression of the oestrogen signalling pathway in *Ido1‐*OE group (NES = −1.51, *p* = 0.0047). The running enrichment score and ranked list metric are displayed.

To further elucidate the molecular mechanisms of *Ido1* in cancer cachexia, we performed transcriptomic analysis of gastrocnemius muscle tissues from both LLC and *Ido1‐*OE groups. Differential gene expression analysis revealed a clear separation between *Ido1‐*OE and LLC cell groups (Figures 2A and S2A). Under the parameter criteria of |log2FC| > 0.263 and *p* < 0.05, 234 differentially expressed genes (DEGs) were identified, comprising 133 upregulated and 101 downregulated genes (Figure 2B). Notably, the expression of MuRF1 and Atrogin1, recognized as key mediators of skeletal muscle atrophy that function as E3 ubiquitin ligases with substrate recognition sites to promote protein ubiquitination [20], was significantly upregulated in the *Ido1‐*OE group (Figure 2C). This finding was further validated at the protein level using western blot analysis (Figure 2D). Concurrently, Gene Ontology (GO) enrichment analysis of biological processes (BP) demonstrated significant activation of pro‐inflammatory processes in the *Ido1‐*OE group. The most significantly enriched terms included ‘inflammatory response’, ‘response to interleukin‐1’ and ‘leukocyte chemotaxis’ (Figure 2E). GO enrichment analysis for cellular component (CC) and molecular function (MF) further indicated that *Ido1‐*OE affected MF related to inflammation, oxidative stress and signal transduction in muscle tissue (Figures S2B and S2C). Intramuscular inflammation was also associated with muscle atrophy. Clinical studies have confirmed that serum levels of inflammatory markers such as IL‐6 and TNF‐α are significantly elevated in cachectic patients and correlate with the extent of muscle loss [21]. Tumour‐driven inflammatory responses, by activating the JAK/STAT and NF‐κB signalling pathways, induced oxidative stress in skeletal muscle, thereby activating protein degradation programmes and ultimately leading to muscle atrophy in cancer cachexia [22]. Furthermore, GSEA indicated significant suppression of the oestrogen signalling pathway in the muscle of the *Ido1‐*OE group (NES = −1.51, *p* = 0.0047; Figure 2F). This downregulation was critically important, as studies have shown that oestrogen plays a key role in maintaining skeletal muscle integrity, particularly in postmenopausal women who experience accelerated loss of muscle mass and strength [23]. Oestrogen receptor α (ERα) was central to the complex regulation of skeletal muscle metabolism and mitochondrial function, underscoring its importance in muscle homeostasis [24]. Clinically, the oestrogen receptor agonists medroxyprogesterone acetate and medroxyprogesterone were first‐line treatments for cancer cachexia. Oestrogen receptor agonists partially improved cachexia symptoms by stimulating appetite and regulating metabolism, but they did not prolong survival [18]. Targeting and precision drugs for the regulation of anabolic and protective pathways were likely to constitute a key approach for the treatment of cancer cachexia. Integrating our data, *Ido1*‐driven metabolic reprogramming not only promoted the activation of inflammatory and muscle protein degradation pathways but also attenuated the protective effects of oestrogen signalling, thereby accelerating the progression of lung cancer–associated cachexia. These results suggested that *Ido1*‐mediated metabolic reprogramming may serve as a molecular switch that suppresses multiple anabolic signalling pathways, including oestrogen and mTOR pathways, to drive muscle wasting.

### 3.3 *Ido1‐*OE Induced a Fast‐to‐Slow Fibre Type Shift and Suppressed mTOR Anabolic Signalling in Skeletal Muscle

**FIGURE 3:**
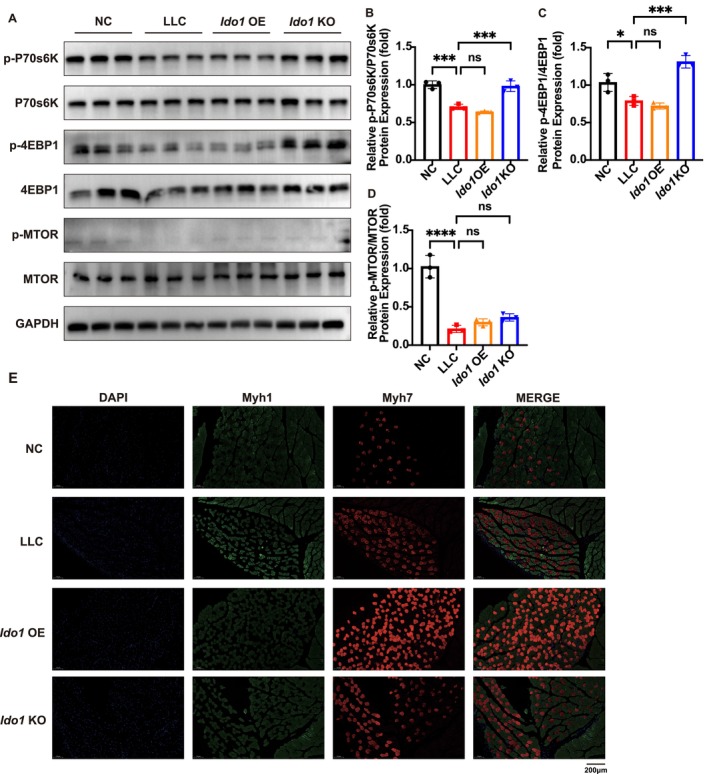
*Ido1‐*OE induced a fast‐to‐slow fibre type shift and suppressed mTOR anabolic signalling in skeletal muscle. (A) Representative western blot images of phosphorylated and total MTOR, 4EBP1 and P70S6K. (B–D) Quantitative analysis of p‐MTOR/MTOR, p‐4EBP1/4EBP1 and p‐P70S6K/P70S6K protein levels. One‐way ANOVA with Sidak's multiple comparisons test was used for statistical analysis. The *p*‐values are denoted with asterisks as follows: not significant (ns); *p < 0.05; **p < 0.01; ***p < 0.001; ****p < 0.0001. (E) Representative immunofluorescence images of gastrocnemius muscle cross sections stained for myosin heavy chain type IIx (Myh1, green) and Type I (Myh7, red). Nuclei are counterstained with DAPI (blue). Scale bar: 200 μm.

We subsequently assessed the activation status of the mTOR anabolic pathway, a core regulator of muscle protein synthesis [25]. Inhibition of this pathway was a known mechanism underlying cachectic muscle atrophy as it directly impeded new protein synthesis [26]. Western blot analysis revealed that phosphorylation levels within this pathway were not significantly suppressed in the *Ido1‐*OE group. Notably, the *Ido1‐KO* group exhibited a marked trend towards restored p‐4EBP1 (*p* < 0.001) and p‐P70S6K (*p* < 0.001) levels, suggesting that *Ido1* KO helped maintain anabolic signalling pathways under tumour burden (Figure 3A–D).

Studies have shown that cancer cachexia led to alterations in human skeletal muscle molecular subtypes [27]. We then investigated the effects of *Ido1* on skeletal muscle fibre types. Immunofluorescence staining of gastrocnemius muscle sections revealed significant alterations in the fibre type composition (Figure 3E). In the *Ido1‐*OE group, the proportion of Type IIx (fast‐twitch) muscle fibres decreased (stained with anti‐Myh1 antibody), whereas the proportion of Type I (slow‐twitch) muscle fibres increased (stained with anti‐Myh7 antibody), indicating a shift from fast‐twitch to slow‐twitch fibre types. This shift represented a hallmark of muscle atrophy. It was primarily driven by the preferential loss of fast‐twitch glycolytic fibres during catabolic states, a pathological response that led to a disproportionate decline in muscle strength and function [28]. The *Ido1‐*KO group exhibited an intermediate phenotype between those of the LLC and *Ido1‐*OE groups, suggesting partial mitigation of cachexia‐induced myofibrillar remodelling. Collectively, these data demonstrated that *Ido1* directly inhibited anabolic processes, leading to muscle mass loss and a shift towards more fatigable slow‐twitch muscle fibre types.

### 3.4 *Ido1*‐OE Reprogrammed Trp Metabolism and Correlated With Cancer‐Associated Cachexia

**FIGURE 4:**
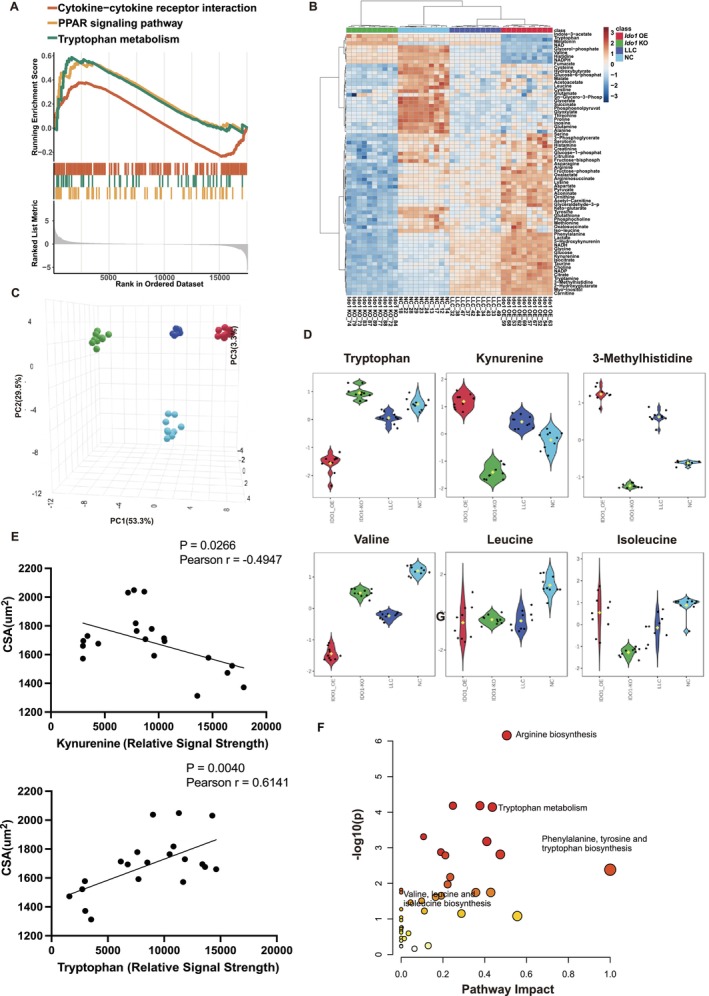
*Ido1*‐OE reprogrammed Trp metabolism and correlated with cachexia‐associated cachexia. (A) GSEA of transcriptomic data from the gastrocnemius muscle. (B) Heatmap of targeted metabolomic profiling of serum samples from the four groups. (C) PCA score plot of serum metabolomic data. (D) PLS‐DA loading plot identifying key metabolites contributing to group separation, including tryptophan, kynurenine, 3‐methylhistidine and branched‐chain amino acids (valine, leucine and isoleucine). (E) Correlation analysis between serum metabolites and muscle atrophy indicators. Each data point represents an individual biological replicate (*n* = 20). The solid line indicates the linear regression fit. (F) Pathway impact analysis integrating metabolomic data, revealing the most significantly altered metabolic pathways, including tryptophan metabolism, phenylalanine/tyrosine/tryptophan biosynthesis and valine/leucine/isoleucine biosynthesis.

Previous studies have demonstrated that metabolic crosstalk mechanisms played a role in cancer cachexia [5]. To comprehensively analyse *Ido1*‐driven metabolic alterations, we performed integrated transcriptomic and targeted metabolomic analyses across the four cohorts. GSEA revealed significant enrichment of pathways associated with cytokine–cytokine receptor interactions, PPAR signalling and Trp metabolism (Figure 4A). These pathways were closely associated with muscle wasting and systemic inflammation in cancer cachexia. Cytokine signalling (e.g., IL‐6 and TNF‐α) and PPAR dysregulation have been demonstrated to promote muscle wasting and fat loss, whereas *Ido1*‐mediated Trp metabolism was known to drive immunosuppression and muscle degeneration [14]. Targeted metabolomic analysis revealed a distinct separation in serum metabolite levels across the four groups (Figures 4B and S3A). PCA further confirmed this pronounced clustering phenomenon; the PC1 dimension (explaining 53.3% of variance) exhibited clear separation, highlighting *Ido1*‐driven metabolic differentiation (Figures 4C and S3B). Correlation analysis of the top 25 metabolites significantly associated with Trp and Kyn was shown in Figure S3C,D. Partial least squares discriminant analysis (PLS‐DA) was performed to identify the most discriminative metabolites of the cachectic phenotype. Key metabolites involved in Trp metabolism and protein degradation, including Kyn and 3‐methylhistidine, were significantly elevated in the Ido1‐OE group. Conversely, essential amino acids linked to protein synthesis, particularly Trp and branched‐chain amino acids (BCAAs), were markedly depleted in Ido1‐OE mice compared to the control groups (Figure 4D). To further clarify the quantitative association between these metabolic shifts and muscle wasting, we performed correlation analyses between serum metabolite concentrations and CSA. Our results showed that serum Trp levels were significantly positively correlated with CSA (*r* = 0.6141, *p* = 0.0040), whereas Kyn levels exhibited a robust negative correlation with CSA (*r* = −0.4947, *p* = 0.0266) (Figure 4E). These findings aligned with those of previous studies that demonstrated that *Ido1* activation promotes Kyn accumulation and disrupts systemic energy metabolism [29]. Furthermore, the depletion of BCAAs (valine, leucine and isoleucine) in the cachexia model further underscored their critical role in muscle wasting [30]. Additional differentially altered metabolites are shown in Figure S3E. Pathway impact analysis further confirmed that pathways involving Trp metabolism, phenylalanine/tyrosine/Trp biosynthesis and valine/leucine/isoleucine biosynthesis were among the most significantly altered (Figure 4F). Collectively, these data indicated that *Ido1* not only accelerated Trp–Kyn metabolism but also drove broader metabolic reprogramming, thereby promoting the development of cancer cachexia.

### 3.5 *Ido1*‐OE Promoted the Proliferation and Migration Capabilities of LLC Cells and Indirectly Induced Atrophy in C2C12 Cells

**FIGURE 5:**
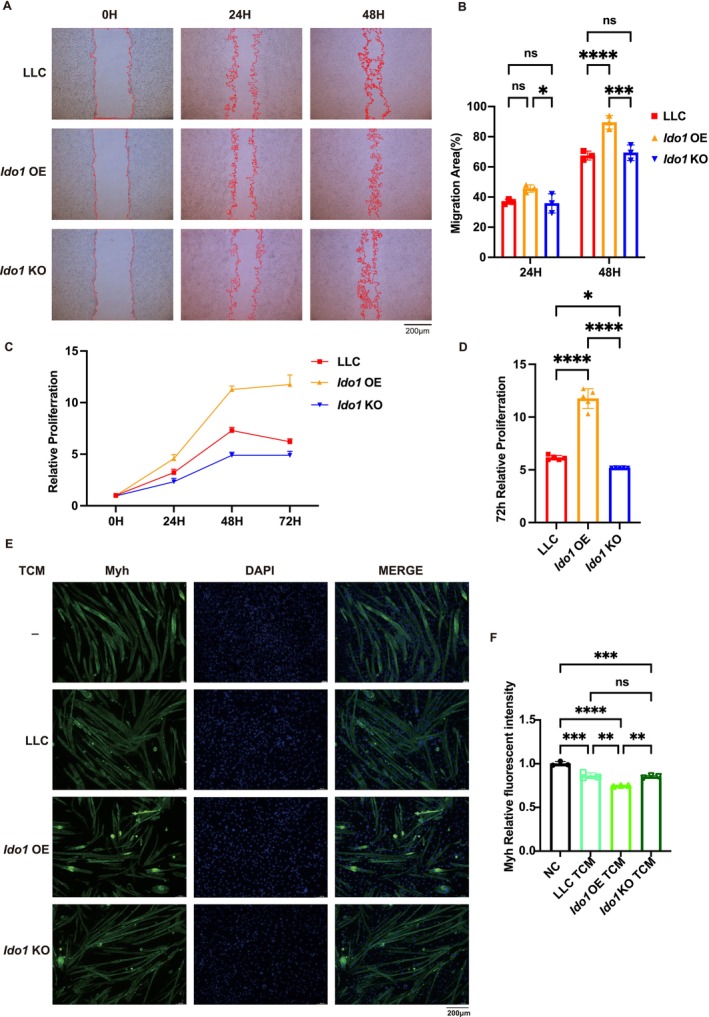
*Ido1‐*OE promoted the proliferation and migration capabilities of LLC cells and indirectly induced atrophy in C2C12 cells. (A) Representative image of the wound healing assay at 0, 24 and 48 h. Scale bar: 200 μm. (B) Quantification of wound closure (migration area) over time. One‐way ANOVA with Sidak's multiple comparisons test was used for statistical analysis. The *p*‐values are denoted with asterisks as follows: not significant (ns); *p < 0.05; **p < 0.01; ***p < 0.001; ****p < 0.0001. (C) Growth curves over 72 h of each group. (D) Relative proliferation at the 72‐h time point of each group. One‐way ANOVA with Sidak's multiple comparisons test was used for statistical analysis. The *p*‐values are denoted with asterisks as follows: not significant (ns); *p < 0.05; **p < 0.01; ***p < 0.001; ****p < 0.0001. (E) Representative immunofluorescence images of C2C12 myotubes treated with TCM from different tumour groups, stained for Myh (green) and DAPI (blue). Scale bar: 200 μm. (F) Quantification of myotube atrophy, shown as relative Myh fluorescent intensity. One‐way ANOVA with Sidak's multiple comparisons test was used for statistical analysis. The *p*‐values are denoted with asterisks as follows: not significant (ns); *p < 0.05; **p < 0.01; ***, *p* < 0.001; ****p < 0.0001.

To investigate the functional role of *Ido1* at the cellular level, we first evaluated the proliferation and migration capabilities of LLC tumour cells with different *Ido1* expression levels. Wound healing assays revealed that compared to LLC cells, *Ido1‐*OE significantly enhanced cell migration; wound closure rates were 45.6% ± 2.4% at 24 h and 89.5% ± 4.5% at 48 h (*p* < 0.0001; Figure 5A,B). CCK‐8 assays similarly demonstrated that *Ido1‐*OE promoted LLC cell proliferation, with a 1.9‐fold increase in the proliferation rate at 72 h compared with that in the LLC group (*p* < 0.0001). Conversely, *Ido1‐*KO cells exhibited reduced proliferation capacity, decreasing to 0.83‐fold that of the LLC group (*p* < 0.0001; Figure 5C,D).

Subsequently, we investigated the effects of tumour *Ido1* expression on C2C12 myoblasts at the cellular level. Differentiated C2C12 myotubes were treated with conditioned medium from LLC, Ido1‐OE and Ido1‐KO tumours. Immunofluorescence staining revealed that compared to the LLC group, TCM from *Ido1‐*OE tumours induced more severe myotube atrophy (*p* < 0.01; Figure 5E,F). These findings indicated that *Ido1* not only enhanced tumour malignancy but also indirectly suppressed skeletal muscle differentiation capacity.

### 3.6 Kyn Directly Induced Myotube Atrophy, Which Was Partially Rescued by Trp Supplementation

**FIGURE 6:**
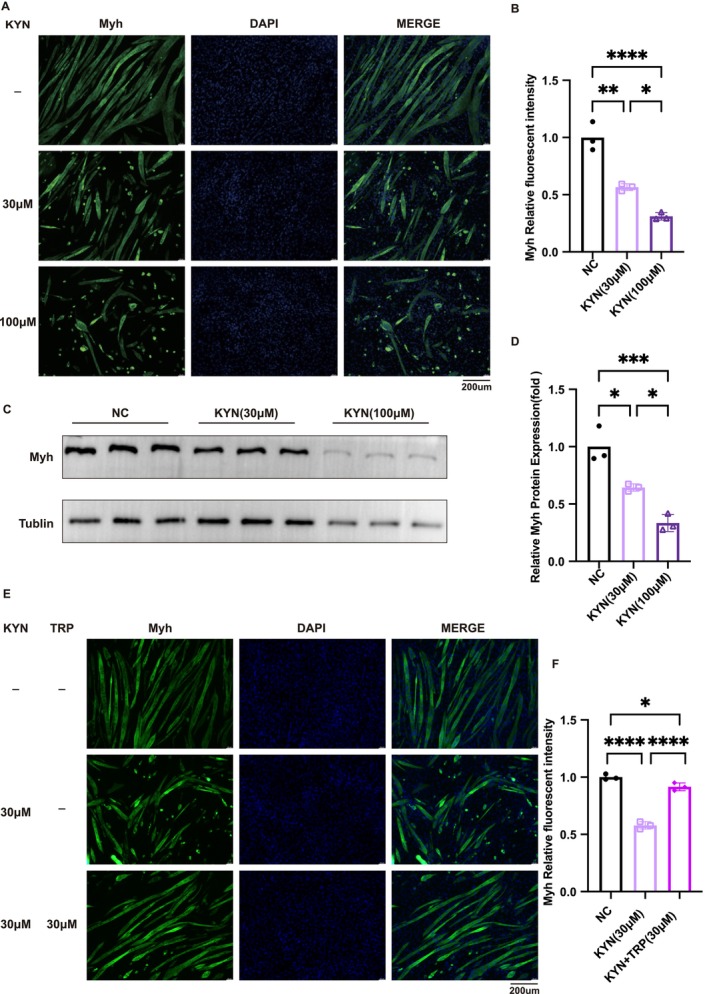
Kyn directly induced myotube atrophy, which was partially rescued by Trp supplementation. (A) Representative immunofluorescence images of C2C12 myotubes treated with different concentrations of Kyn (30 and 100 μM) or control (NC), stained for Myh (green) and DAPI (blue). Scale bar: 200 μm. (B) Quantification of myotube atrophy shown as relative Myh fluorescent intensity. One‐way ANOVA with Sidak's multiple comparisons test was used for statistical analysis. The *p*‐values are denoted with asterisks as follows: not significant (ns); *p < 0.05; **p < 0.01; ***p < 0.001; ****p < 0.0001. (C) Representative Western blot images of Myh protein levels. Tubulin serves as the loading control. (D) Quantification of relative Myh protein expression. One‐way ANOVA with Sidak's multiple comparisons test was used for statistical analysis. The *p*‐values are denoted with asterisks as follows: not significant (ns); *p < 0.05; **p < 0.01; ***p < 0.001; ****p < 0.0001. (E) Representative immunofluorescence images of C2C12 myotubes treated with Kyn (30 μM) alone or in combination with Trp (30 μM). Scale bar: 200 μm. (F) Quantification of myotube atrophy shown as relative Myh fluorescent intensity. One‐way ANOVA with Sidak's multiple comparisons test was used for statistical analysis. The *p*‐values are denoted with asterisks as follows: not significant (ns); *p < 0.05; **p < 0.01; ***p < 0.001; ****p < 0.0001.

Based on our previous finding that tumours overexpressing *Ido1* exhibited enhanced Kyn production (Figure 4D), we sought to determine whether Kyn directly causes myotubular atrophy. Differentiated C2C12 myotubes were treated with different concentrations of Kyn (30 and 100 μM). Immunofluorescence analysis revealed a dose‐dependent decrease in Myh expression; fluorescence intensity in the 30 and 100 μM Kyn‐treated groups decreased to 0.57 ± 0.03 and 0.31 ± 0.03, respectively (control: 1.0 ± 0.13; *p* < 0.01 and *p* < 0.0001; Figure 6A,B). Western blot analysis further confirmed this effect, showing significantly reduced Myh protein levels at both concentrations (*p* < 0.05, *p* < 0.001; Figure 6C,D). These results indicated that Kyn, a key Trp metabolite that accumulated in high‐*Ido1* tumours, induced significant atrophy of myotubes.

To investigate whether restoring Trp supply could counteract Kyn‐induced atrophy, C2C12 myotubes were cotreated with 30 μM Kyn and 30 μM Trp. Notably, Trp supplementation partially reversed Kyn‐induced myotube atrophy, as evidenced by Myh fluorescence intensity recovery from 0.58 ± 0.03 to 0.92 ± 0.03 (*p* < 0.0001; Figure 6E,F). This demonstrated that the balance between Trp and its metabolite, Kyn, was critical for maintaining muscle protein homeostasis.

Combined with our previous observations of *Ido1*‐driven Trp depletion and Kyn accumulation, as well as *Ido1‐*OE TCM‐induced muscle atrophy, this study established a direct causal role for Kyn in myotubular wasting and highlighted the potential of Trp supplementation as a strategy to improve *Ido1*‐mediated muscle defects.

### 3.7 The *Ido1* Inhibitor PAL Relieved Cancer Cachexia

**FIGURE 7:**
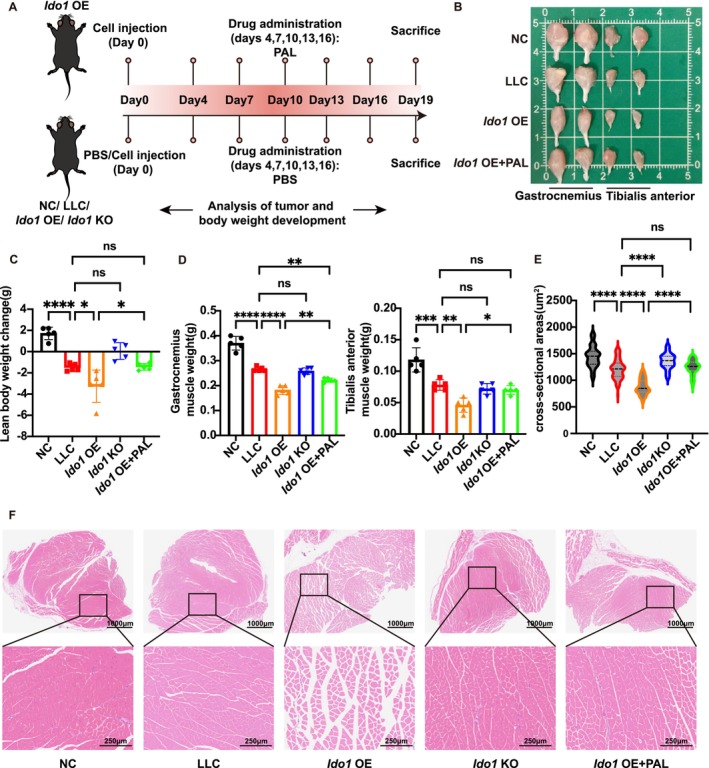
The *Ido1* inhibitor PAL relieved cancer cachexia. (A) Schematic diagram of the experimental timeline. (B) The typical picture of the gastrocnemius and anterior tibial muscle from each group of mice. (C) The lean body weight changes of each group of mice. One‐way ANOVA with Sidak's multiple comparisons test was used for statistical analysis. The *p*‐values are denoted with asterisks as follows: not significant (ns); *p < 0.05; **p < 0.01; ***p < 0.001; ****p < 0.0001. (D) The gastrocnemius and anterior tibial muscle weights in each group of mice. One‐way ANOVA with Sidak's multiple comparisons test was used for statistical analysis. The *p*‐values are denoted with asterisks as follows: not significant (ns); *p < 0.05; **p < 0.01; ***p < 0.001; ****p < 0.0001. (E) The CSA of each group of mice. One‐way ANOVA with Sidak's multiple comparisons test was used for statistical analysis. The *p*‐values are denoted with asterisks as follows: not significant (ns);*p < 0.05; **p < 0.01; ***p < 0.001; ****p < 0.0001. (F) The typical pictures of cross‐sectional H&E‐stained histopathological images of the gastrocnemius muscle from each group of mice.

To evaluate the potential of targeting *Ido1* to treat cancer cachexia, we administered the *Ido1* inhibitor PAL (15 mg/kg) to *Ido1‐*OE tumour‐bearing mice on Days 4, 7, 10, 13 and 16 after tumour inoculation (Figure 7A). This specific dosage was selected as the optimal concentration based on our preliminary dose‐escalation pilot study, which demonstrated that 15 mg/kg PAL effectively restored muscle mass without further incremental benefits at higher doses (Figure S4A–C). CCK‐8 assays were performed to evaluate the direct effect of PAL on LLC cell proliferation, and the results indicated that PAL did not exhibit significant cytotoxicity (Figure S4D). PAL was a natural *Ido1* inhibitor with an isoquinoline alkaloid structure that had been reported to possess multiple bioactivities, including anti‐inflammatory, anti‐tumour, cardiovascular protective and metabolic regulatory functions [31]. Clinically, herbs containing PAL, such as *Phellodendron amurense* and *Coptis chinensis* , had been used to relieve various acute conditions and care for chronic metabolic diseases based on the traditional Chinese medicine theory [32, 33]. The molecular docking results showed PAL exhibited favourable binding with the IDO1 protein, as evidenced by a docking score of −4.867 and an MM/GBSA binding free energy of −13.54 kcal/mol. The molecule adopted a rigid quasi‐planar structure that stably embedded into the protein's active pocket. Analysis of key interactions further confirmed a strong binding affinity between the PAL and IDO1 (Figure S5A–C). Then, in the present study, SPF‐grade C57BL/6J mice were euthanized on Day 19 to analyse tumour growth and muscle atrophy. Consistent with previous findings, *Ido1‐*OE tumours exhibited the most pronounced cachectic phenotype: reduced lean body weight (*p* < 0.05), decreased gastrocnemius (*p* < 0.0001) and tibialis anterior muscle (*p* < 0.01) weights and diminished muscle fibre CSA (*p* < 0.0001). Visual inspection of the hind limbs and dissected muscles further confirmed severe muscle atrophy in the *Ido1‐*OE group (Figures 7B and S4E). Notably, compared to untreated *Ido1‐*OE mice, PAL treatment delayed tumour growth (Figure S4F), mitigated lean body weight loss (*p* < 0.05; Figure 7C), increased gastrocnemius (*p* < 0.01) and tibialis anterior muscle (*p* < 0.05) weights (Figure 7D) and increased the muscle fibre CSA (*p* < 0.0001; Figure 7E,F). Crucially, PAL treatment restored the muscle weight and fibre size to nearly those of the *Ido1‐*KO group, indicating that pharmacological inhibition of *Ido1* can effectively recapitulate the genetic knockout phenotype. These findings established *Ido1* as a viable therapeutic target for alleviating cancer cachexia, validated PAL's efficacy in preserving muscle mass and highlighted the translational potential of *Ido1* inhibition for cachexia management.

### 3.8 Molecular Mechanism of the *Ido1* Inhibitor PAL in Alleviating Skeletal Muscle Atrophy

**FIGURE 8:**
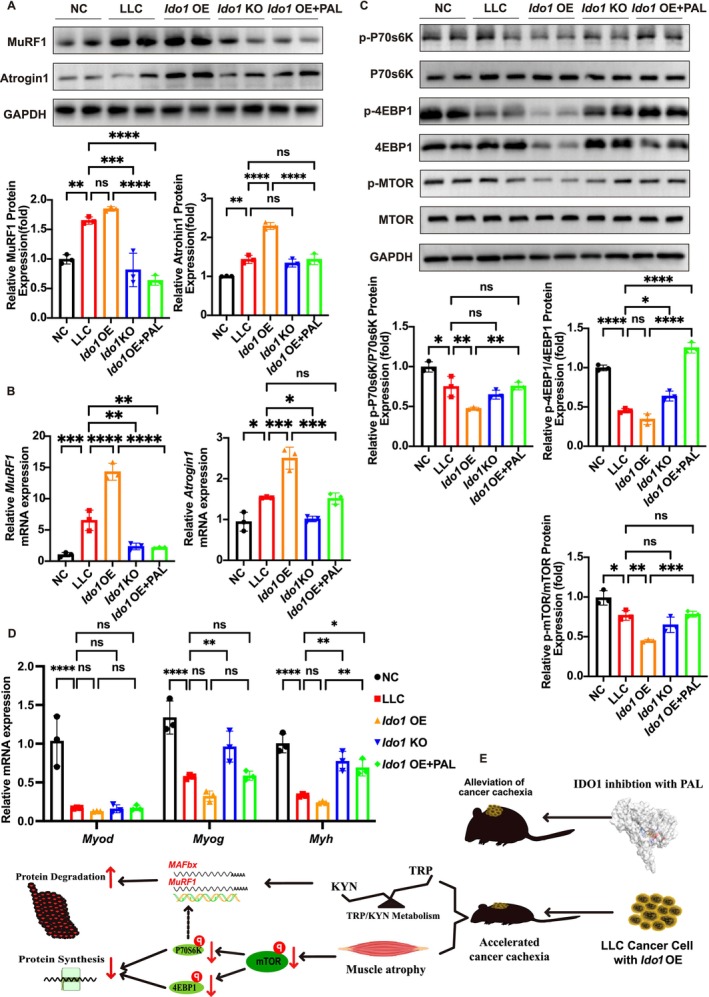
Molecular mechanism of the *Ido1* inhibitor PAL in alleviating skeletal muscle atrophy. (A) Representative western blot images of MuRF1 and Atrogin1 protein expression in the gastrocnemius muscle. Quantitative analysis of MuRF1 and Atrogin1 protein levels. One‐way ANOVA with Sidak's multiple comparisons test was used for statistical analysis. The *p*‐values are denoted with asterisks as follows: not significant (ns); *p < 0.05; **p < 0.01; ***p < 0.001; ****p < 0.0001. (B) PAL treatment alters the mRNA expression of genes involved in muscle atrophy and regeneration. Quantitative RT‐PCR analysis of MuRF1 and Atrogin1. One‐way ANOVA with Sidak's multiple comparisons test was used for statistical analysis. The *p*‐values are denoted with asterisks as follows: not significant (ns); *p < 0.05; **p < 0.01; ***p < 0.001; ****p < 0.0001. (C) Representative western blot images of phosphorylated and total mTOR, 4EBP1 and P70S6K protein expression in the gastrocnemius muscle. Quantitative analysis of p‐MTOR/MTOR, p‐4EBP1/4EBP1 and p‐P70S6K/P70S6K protein levels. One‐way ANOVA with Sidak's multiple comparisons test was used for statistical analysis. The *p*‐values are denoted with asterisks as follows: not significant (ns);*p < 0.05; **p < 0.01; ***p < 0.001; ****p < 0.0001. (D) PAL treatment alters the mRNA expression of genes involved in muscle atrophy and regeneration. Quantitative RT‐PCR analysis of Myod, Myog and Myh. Two‐way ANOVA with Sidak's multiple comparisons test was used for statistical analysis. The *p*‐values are denoted with asterisks as follows: not significant (ns); *p < 0.05; **p < 0.01; ***p < 0.001; ****p < 0.0001. (E) The mechanism diagram of cancer *Ido1* OE promotes cancer cachexia progression by reprogramming tryptophan metabolism, leading to KYN accumulation and TRP depletion, thereby affecting protein synthesis and degradation. PAL treatment reprogramming TRP metabolism and alleviates the cancer cachexia progression.

We further validated the ameliorative effect of the *Ido1* inhibitor PAL on cancer cachexia at the molecular level. Western blot analysis of gastrocnemius muscle revealed that PAL treatment significantly downregulated the protein expression of ubiquitin ligases MuRF1 and Atrogin1 in *Ido1‐*OE tumour‐bearing mice (*p* < 0.0001 and *p* < 0.0001; Figure 8A). Consistent with this, the mRNA levels of *Murf1* and *Atrogin1* were also significantly reduced in the PAL‐treated group compared to those in the untreated *Ido1‐*OE group (*p* < 0.0001, *p* < 0.001; Figure 8B), indicating that PAL inhibited muscle protein degradation. Concurrently, PAL treatment restored the activity of the mTOR anabolic signalling pathway, significantly increasing the phosphorylation levels of mTOR and its downstream effectors, 4EBP1 and p70S6K (*p* < 0.01, *p* < 0.0001 and *p* < 0.01; Figure 8C). This indicated that PAL alleviated defects in protein synthesis in cachectic muscles.

We further assessed the expression of the key myogenic factors. PAL treatment elevated mRNA levels of *Myod* and *Myog*, although without statistical significance (Figure 8D), both of which are essential for myoblast differentiation and muscle regeneration [34]. Additionally, the expression of the *Myh* gene encoding the myosin heavy chain recovered following PAL treatment (*p* < 0.01). Collectively, these data indicated that the *Ido1* inhibitor, PAL, exerted a protective effect against muscle atrophy by synergistically inhibiting protein degradation, enhancing protein synthesis and promoting myogenic differentiation. In summary, cancer *Ido1*‐OE promoted cancer cachexia progression by reprogramming Trp metabolism, leading to Kyn accumulation and Trp depletion, thereby affecting protein synthesis and degradation (Figure 8E). PAL treatment reprogrammed Trp metabolism and alleviated the cancer cachexia progression.

## 4 Discussion

In the present study, we investigated that tumour‐derived *Ido1* exacerbated cancer cachexia by reprogramming the Trp metabolism, particularly the Trp–Kyn pathway, thereby activating muscle catabolism and suppressing anabolism. We employed an LLC model with genetic manipulation of *Ido1* expression to systematically evaluate its impact on tumour growth, muscle wasting and global metabolic profiles. *Ido1*‐OE remodelled the skeletal muscle transcriptome by suppressing anabolic pathways and activating catabolic pathways. Based on the finding that *Ido1*‐OE reprogrammed Trp metabolism and correlated it with cancer‐associated cachexia, we found that Kyn, a downstream metabolite of Trp, directly induced myotube atrophy. This study systematically elucidated *Ido1* as a key driver of systemic metabolic reprogramming and skeletal muscle atrophy, positioning it as a crucial mediator of cancer cachexia.

Clinical studies indicated that lung cancers overexpressing *IDO1* accelerate cachexia progression [35], and our data provide mechanistic support for this observation. Consistent with *Ido1*'s classical role in tumour immune evasion [36], we found that *Ido1‐*OE promoted tumour growth. Notably, *Ido1‐*OE mice exhibited significant muscle loss and reduced muscle fibre CSA, highlighting *Ido1*'s dual detrimental effects on tumour progression and host muscle integrity. Transcriptomic and metabolomic analyses further revealed that *Ido1‐*OE activated inflammatory and proteolytic pathways (e.g., MuRF1/Atrogin1 upregulation) while suppressing protective signalling pathways, such as oestrogen receptor α–mediated mitochondrial maintenance [37]. Given the established role of oestrogen signalling in preserving muscle weight and mitochondrial function, its inhibition may represent a novel mechanism by which *Ido1* exacerbated muscle wasting. Concurrently, serum metabolomic analysis revealed a characteristic metabolic profile marked by Trp depletion, Kyn accumulation and BCAA metabolism disruption [38, 39]. Because BCAAs, particularly leucine, were potent activators of the mTORC1 pathway, their depletion likely contributed to anabolic resistance in cachexia. Our integrated analysis provided a clearer link between *Ido1*‐driven metabolic shifts and the suppression of muscle anabolic signalling. Specifically, the overexpression of *Ido1* resulted in a characteristic metabolic profile of Trp exhaustion and Kyn accumulation. The GSEA results indicated that these metabolic changes were accompanied by significant transcriptomic alterations in the oestrogen signalling and mTOR pathways, both of which were critical for maintaining muscle protein proteostasis. To validate these findings, we performed western blot analysis, which confirmed that *Ido1*‐KO significantly increased the phosphorylation levels of the mTOR pathway. The concurrent depletion of essential amino acids and the downregulation of these major growth‐promoting pathways suggested that *Ido1*‐mediated metabolic reprogramming created a multifaceted inhibitory environment. This convergence of nutrient deficiency and suppressed anabolic signalling effectively explained the accelerated muscle protein degradation and impaired synthesis observed in *Ido1*‐OE model, ultimately driving the progression of cancer cachexia. Collectively, these data revealed *Ido1*‐driven systemic metabolic dysregulation culminating in a catabolic state that overwhelms anabolic processes.

A key mechanism identified in this study was the direct myotoxic effect of Kyn. In vitro experiments confirmed that Kyn induced C2C12 myotube atrophy in a dose‐dependent manner. This finding aligned with those of previous studies on Trp metabolism abnormalities in models of cachexia [40]. Notably, Trp supplementation partially reversed Kyn‐induced myotubular atrophy, indicating that muscle wasting was driven not only by toxic metabolite accumulation but also by essential amino acid depletion. This dual mechanism highlighted the therapeutic potential of combining nutritional support with *Ido1* inhibition.

This study confirmed that the natural product PAL, an *Ido1* inhibitor, effectively alleviated cancer cachexia. PAL was an isoquinoline alkaloid that was recorded in the Chinese Pharmacopoeia and was present in the herbs of *P. amurense* and *C. chinensis* [32, 33]. PAL has been reported to possess multiple bioactivities, including anti‐inflammatory, anti‐tumour and cardiovascular protective effects, as well as metabolic regulatory functions. It was clinically used to relieve various acute conditions and care for chronic metabolic diseases based on the traditional Chinese medicine theory [S2]. PAL treatment not only improved muscle weight in *Ido1‐*OE tumour‐bearing mice but also exerted a mechanistic effect by downregulating MuRF1/Atrogin1 and restoring mTOR anabolic signalling. The dual action of inhibiting protein degradation and promoting protein synthesis directly targeted the two core pathological processes of cachexia. Furthermore, PAL partially restored the expression of *MyoD*, *Myog* and *Myh*, suggesting an enhanced muscle regenerative capacity. These findings highlighted the translational medical significance of PAL and its potential use as a therapeutic agent.

In the present study, we found *Ido1* had been a key driver of Trp/Kyn metabolic disorders and skeletal muscle atrophy, positioning it as a crucial mediator in the progression of cancer cachexia. Importantly, *Ido1* inhibitor PAL exerted a Trp/Kyn metabolic reprogramming effect and reversed *Ido1*‐OE exacerbating cancer cachexia. A limitation of this study is that although we demonstrated the independent anti‐cachectic effects of *Ido1* inhibition via PAL and the rescue potential of Trp supplementation in vitro, their direct in vivo comparison and potential synergistic effects remain to be fully explored. Given that cancer cachexia involved both the accumulation of the toxic metabolite Kyn and the depletion of the essential nutrient Trp, combining *Ido1* inhibitors with nutritional Trp support may offer a more potent therapeutic strategy by simultaneously suppressing muscle proteolysis and promoting anabolic synthesis. Future studies incorporating dose–response titrations for such combination therapies in diverse preclinical models will be essential to validate this dual‐targeting approach.

## 5 Conclusions

In summary, our study identifies that *Ido1*‐mediated Trp–Kyn reprogramming accelerates the development of tumour cachexia. This metabolic shift collectively suppresses protein synthesis by inhibiting mTOR signalling and accelerates protein degradation via the activation of E3 ubiquitin ligases, thereby promoting skeletal muscle atrophy and cachexia progression. Furthermore, the *Ido1* inhibitor PAL effectively reverses these pathological processes by restoring metabolic homeostasis and maintaining muscle protein balance. These findings establish the targeting of *Ido1*‐mediated metabolic reprogramming as a promising and mechanism‐directed therapeutic strategy for managing cancer cachexia.

## Funding

This work was supported by grants from the National Natural Science Foundation of China (82272925 and 82274151) and the Shanghai Jiao Tong University School of Medicine Research Physician Program (20240815).

## Ethics Statement

Animal experimental designs and protocols were reviewed and approved by the Institutional Animal Care and Use Committee of Shanghai Sixth People's Hospital, affiliated with Shanghai Jiao Tong University School of Medicine (Protocol No. DWSY2023‐0174).

## Conflicts of Interest

The authors declare no conflicts of interest.

## Supporting information


**Figure S1:** Effects of *Ido1* on genetic alterations, survival outcomes in human lung cancer models and tumour progression in animal models. (A) Frequency of *IDO1* genetic alterations in the lung cancer cohort, including mutations (black), structural variations (blue), copy number amplifications (green), deep deletions (red) and multiple alterations (grey). (B) Kaplan–Meier survival analysis comparing overall survival between IDO1‐altered (red) and non‐altered (blue) patient groups. (C) Lentiviral plasmid map with *Ido1* KO (based on CRISPR/CAS9 method) and OE. (D) Western blot analysis showing *Ido1* protein expression levels in LLC, *Ido1*‐OE and three CRISPR knockout lines (sg1, sg2, sg3). GAPDH served as an internal control. One‐way ANOVA with Sidak's multiple comparisons test was used for statistical analysis. The *p*‐values are denoted with asterisks as follows: not significant (ns); *p < 0.05; **p < 0.01; ***, *p* < 0.001; ****p < 0.0001. (E) RT‐qPCR showing *Ido1* mRNA expression levels in LLC, *Ido1*‐OE and three CRISPR knockout lines (sg1, sg2, sg3). GAPDH served as an internal control. One‐way ANOVA with Sidak's multiple comparisons test was used for statistical analysis. The *p*‐values are denoted with asterisks as follows: not significant (ns);*p < 0.05; **p < 0.01; ***p < 0.001; ****p < 0.0001. (F) The tumour weight curve of each group of mice.

**Figure S2:** Correlation analysis and functional enrichment of LLC and *Ido1*‐OE skeletal muscle samples based on transcriptomic results. (A) Correlation matrix of LLC and *Ido1*‐OE gastrocnemius tissue samples based on transcriptomic profiles. (B) Enrichment plot of cellular component terms identified by Gene Ontology (GO) analysis. Bar height represents the number of genes associated with each term, with colour gradient reflecting statistical significance (*p*‐value). (C) Enrichment plot of molecular function terms based on GO analysis. Bars indicate gene counts, colour‐coded according to *q*‐value thresholds.

**Figure S3:** Serum metabolome analysis and compound correlation studies. (A) Heatmap displaying pairwise correlation coefficients of detected metabolites across experimental groups. (B) PCA of metabolome data for each group. (C) Top 25 metabolites most strongly correlated with tryptophan levels. (D) The 25 metabolites most strongly correlated with kynurenine levels. (E) Violin plot comparing 5‐hydroxycanine uric acid and creatinine levels across the four experimental groups.

**Figure S4:** The *Ido1* inhibitor PAL relieved cancer cachexia. (A) The gastrocnemius muscle weight in each group of mice. One‐way ANOVA with Sidak's multiple comparisons test was used for statistical analysis. The *p*‐values are denoted with asterisks as follows: not significant (ns); *p < 0.05; **p < 0.01; ***p < 0.001; ****p < 0.0001. (B) The anterior tibial muscle weight in each group of mice. One‐way ANOVA with Sidak's multiple comparisons test was used for statistical analysis. The *p*‐values are denoted with asterisks as follows: not significant (ns); *p < 0.05; **p < 0.01; ***p < 0.001; ****p < 0.0001. (C) The lean body weight changes of each group of mice. One‐way ANOVA with Sidak's multiple comparisons test was used for statistical analysis. P‐values are denoted with asterisks as follows: not significant (ns); *p < 0.05; **p < 0.01; ***p < 0.001; ****p < 0.0001. (D) Growth curves over 48 h of each group. (E) The typical picture of the hindlimb muscle from each group of mice. (F) The tumour weight curve of each group of mice. One‐way ANOVA with Sidak's multiple comparisons test was used for statistical analysis. The *p*‐values are denoted with asterisks as follows: not significant (ns); *p < 0.05; **p < 0.01; ***p < 0.001; ****p < 0.0001.

**Figure S5:** Docking of palmatine with IDO1. (A) Chemical structure and 2D interaction diagram of palmatine. (B) The optimal binding pocket of IDO1 (PDB: 5EK2) obtained from the open‐source website Cavity Plus. Parameters: Box centre (Å) 21.25, −2.0, −22.25; box size (Å) 21.5, 23.0, 24.5; volume (Å^3^) 2360.25; surface area (Å^2^) 1613.00. (C) Molecular docking of IDO1 with palmatine. Palmatine exhibited a rigid quasi‐planar structure. After docking, the docking score is −4.867, and the MMGBSA binding free energy is −13.54 kcal/mol. Figures were generated using PYMOL 3.1.4.1.

**Table S1:** The primers for the qPCR experiment.

**Table S2:** Raw dataset for Figure S1A,B.

**Table S3:** Raw dataset for statistical analysis of all figures.

**Data S1:** Supplementary references.

**Data S2:** Supporting Information.

## Data Availability

The data used in this study are available upon request from the lead contact. The raw datasets of statistics were included in supplementary tables.
