# Supplementary figures and images for: Cancer IDO1‐Mediated Tryptophan–Kynurenine Metabolic Reprogramming to Drive Skeletal Muscle Atrophy and Cachexia Acceleration
Source: J Cachexia Sarcopenia Muscle. 2026 Apr 24;17(3):e70295. doi: 10.1002/jcsm.70295 (PMC13107547; doi:10.1002/jcsm.70295)

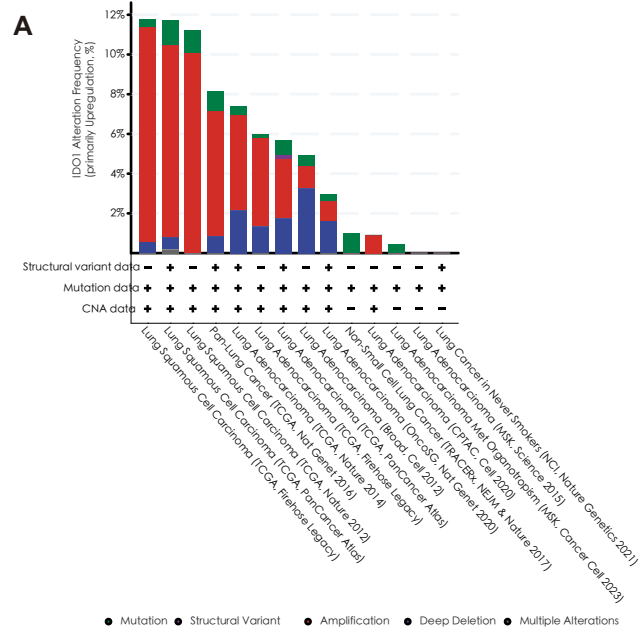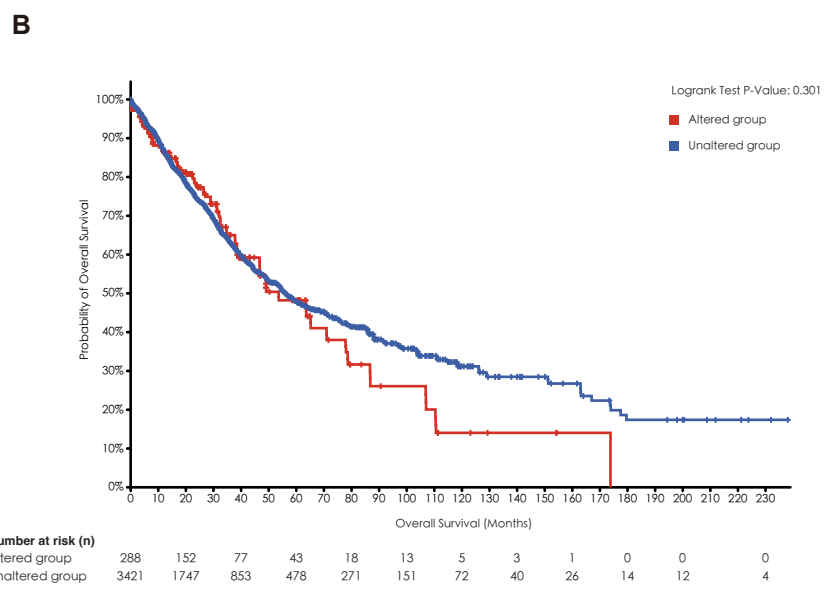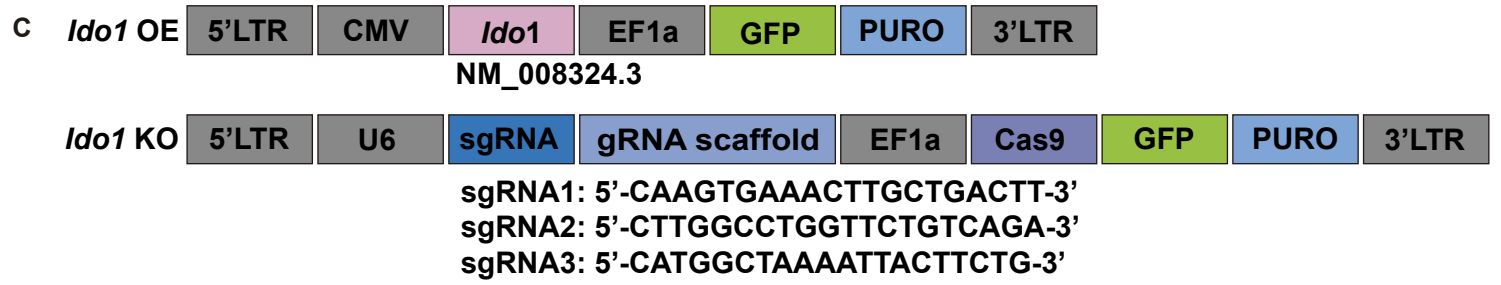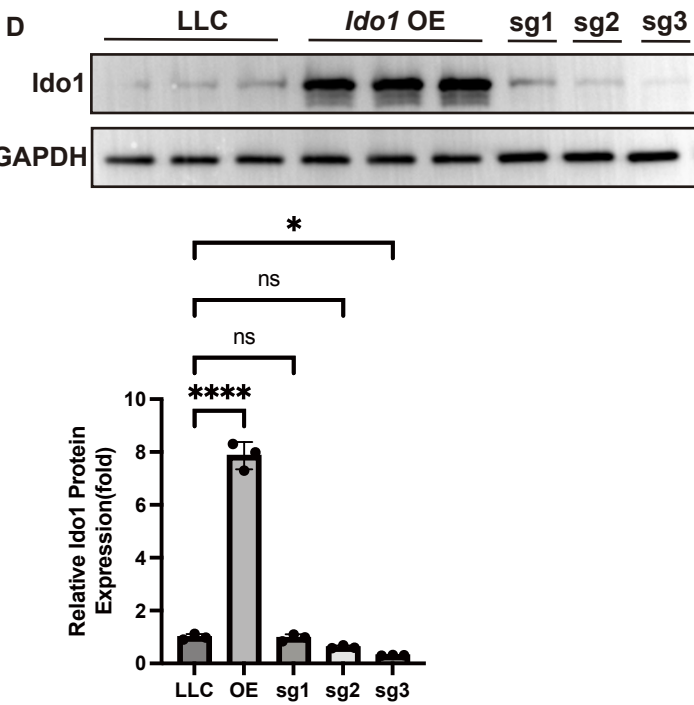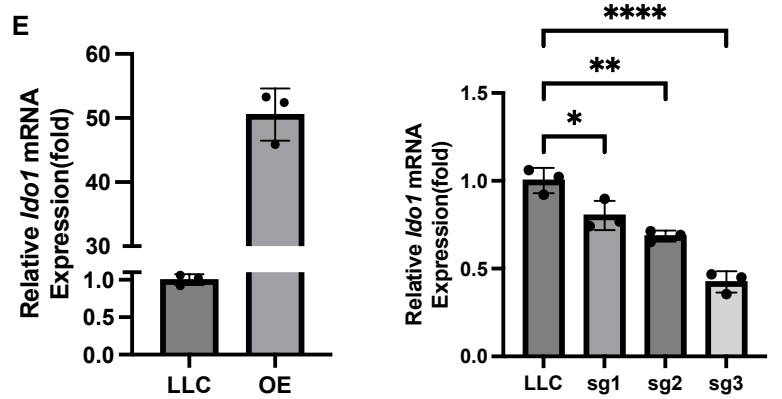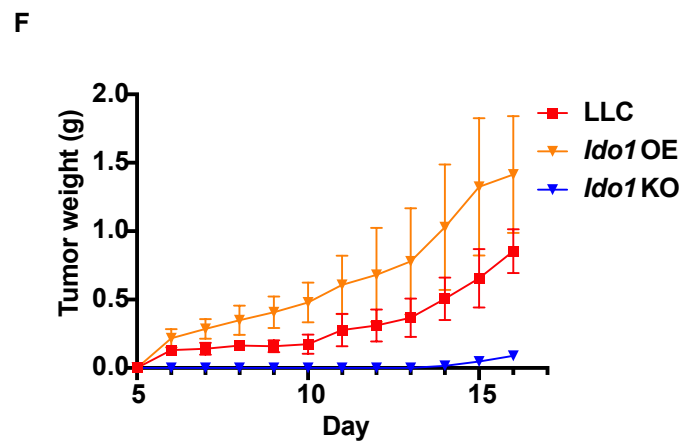

Supplement: Supplementary file 1 — Figure S1: Effects of Ido1 on genetic alterations, survival outcomes in human lung cancer models and tumour progression in animal models. (A) Frequency of IDO1 genetic alterations in the lung cancer cohort, including mutations (black), structural variations (blue), copy number amplifications (green), deep deletions (red) and multiple alterations (grey). (B) Kaplan–Meier survival analysis comparing overall survival between IDO1‐altered (red) and non‐altered (blue) patient groups. (C) Lentiviral plasmid map with Ido1 KO (based on CRISPR/CAS9 method) and OE. (D) Western blot analysis showing Ido1 protein expression levels in LLC, Ido1‐OE and three CRISPR knockout lines (sg1, sg2, sg3). GAPDH served as an internal control. One‐way ANOVA with Sidak's multiple comparisons test was used for statistical analysis. The p‐values are denoted with asterisks as follows: not significant (ns); *p < 0.05; **p < 0.01; ***, p < 0.001; ****p < 0.0001. (E) RT‐qPCR showing Ido1 mRNA expression levels in LLC, Ido1‐OE and three CRISPR knockout lines (sg1, sg2, sg3). GAPDH served as an internal control. One‐way ANOVA with Sidak's multiple comparisons test was used for statistical analysis. The p‐values are denoted with asterisks as follows: not significant (ns);*p < 0.05; **p < 0.01; ***p < 0.001; ****p < 0.0001. (F) The tumour weight curve of each group of mice. [file JCSM-17-e70295-s009.pdf]

A

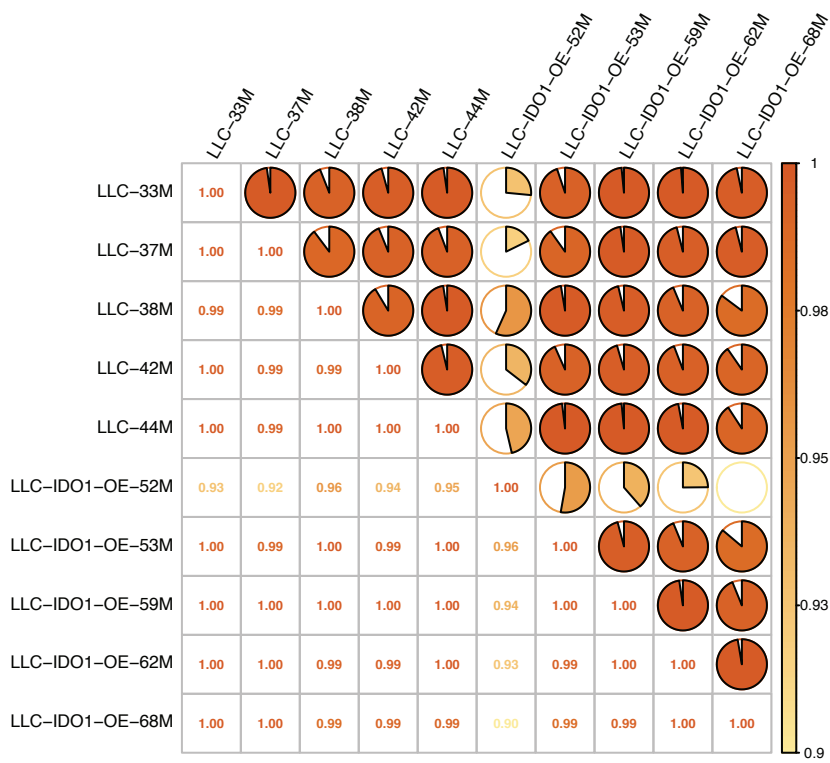

B

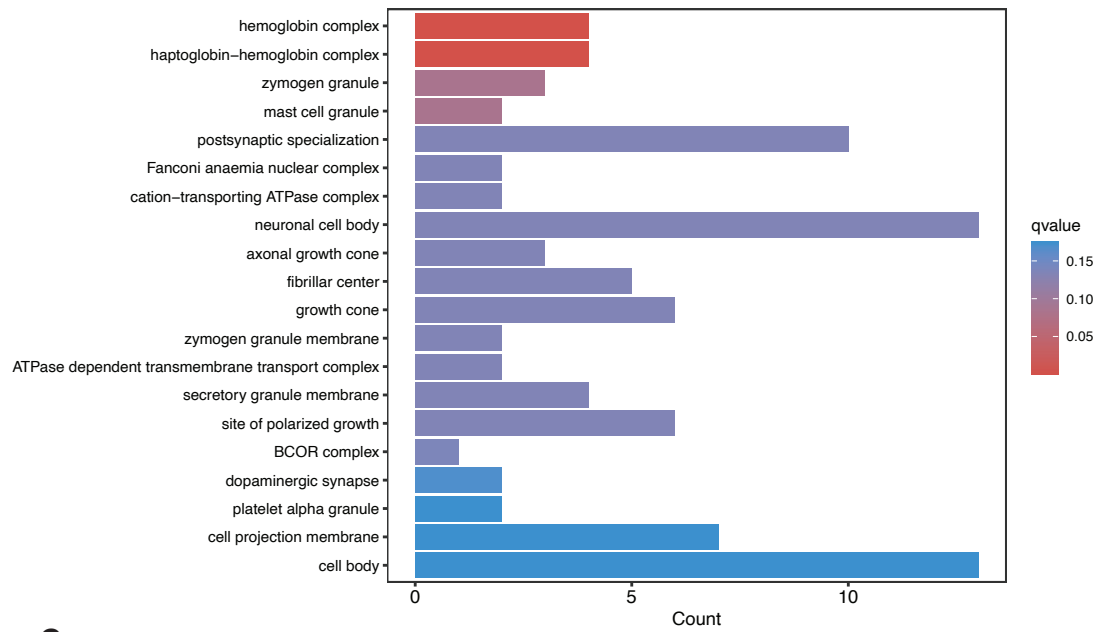

C

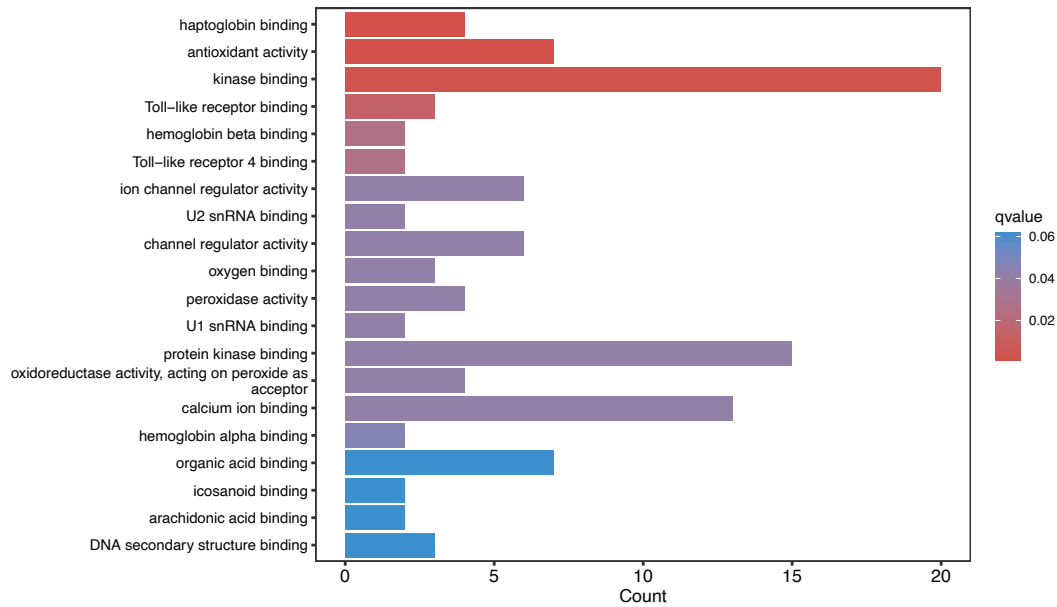

Supplement: Supplementary file 2 — Figure S2: Correlation analysis and functional enrichment of LLC and Ido1‐OE skeletal muscle samples based on transcriptomic results. (A) Correlation matrix of LLC and Ido1‐OE gastrocnemius tissue samples based on transcriptomic profiles. (B) Enrichment plot of cellular component terms identified by Gene Ontology (GO) analysis. Bar height represents the number of genes associated with each term, with colour gradient reflecting statistical significance (p‐value). (C) Enrichment plot of molecular function terms based on GO analysis. Bars indicate gene counts, colour‐coded according to q‐value thresholds. [file JCSM-17-e70295-s002.pdf]

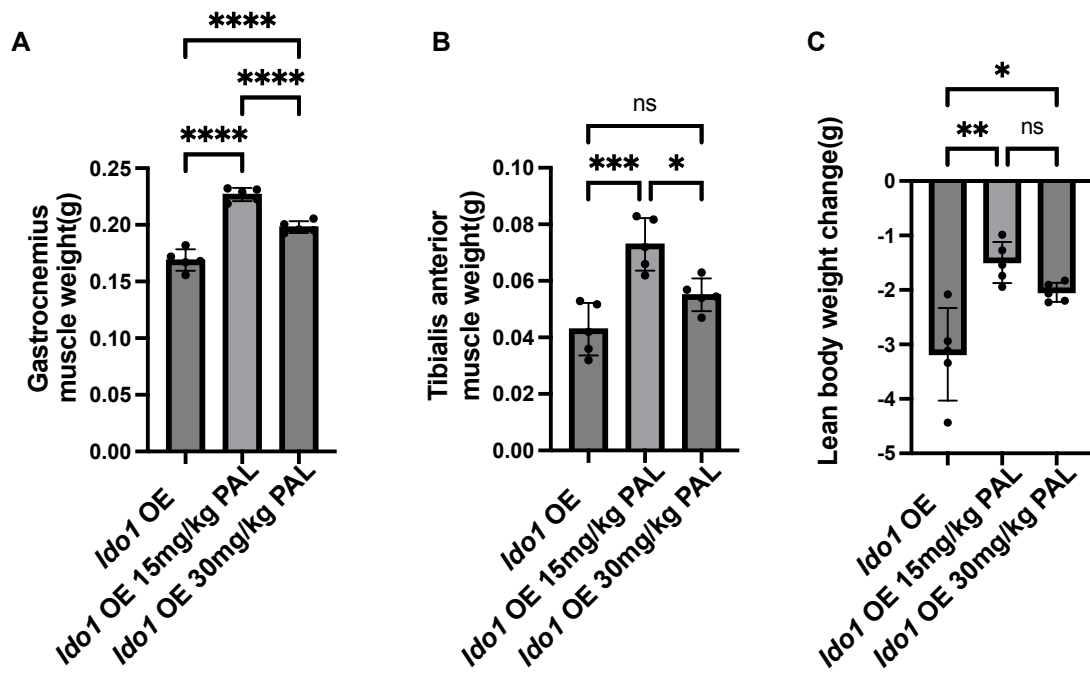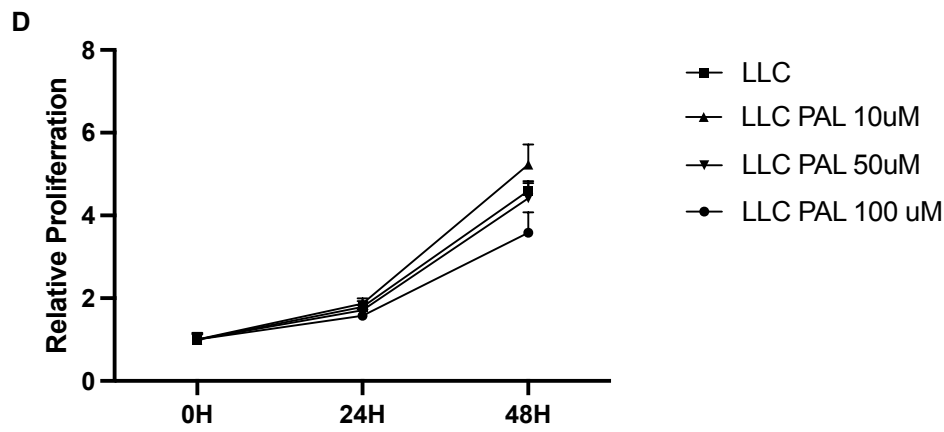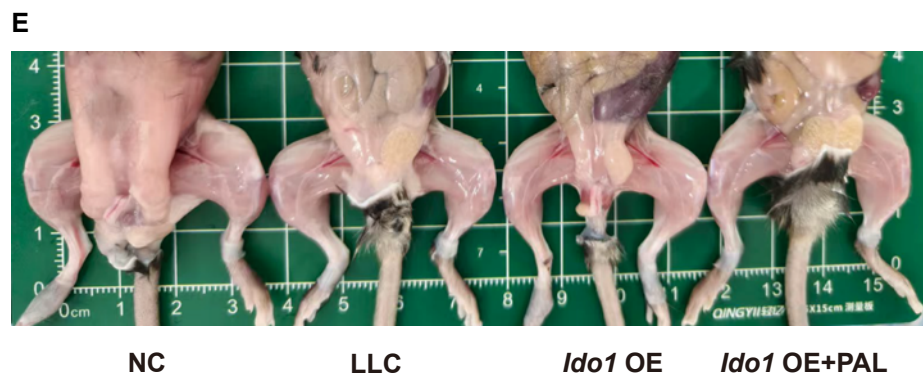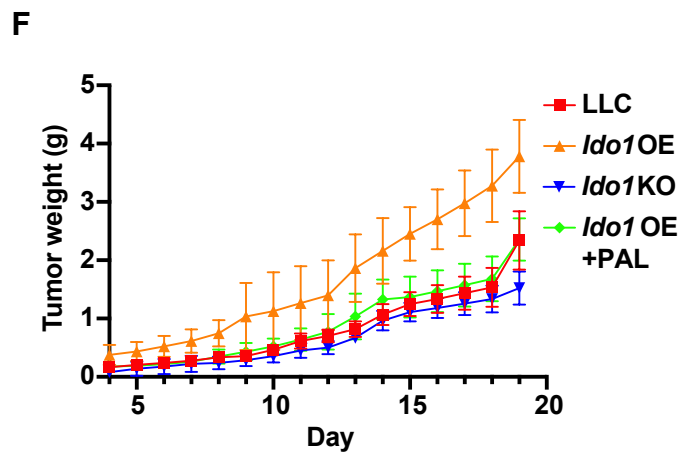

Supplement: Supplementary file 4 — Figure S4: The Ido1 inhibitor PAL relieved cancer cachexia. (A) The gastrocnemius muscle weight in each group of mice. One‐way ANOVA with Sidak's multiple comparisons test was used for statistical analysis. The p‐values are denoted with asterisks as follows: not significant (ns); *p < 0.05; **p < 0.01; ***p < 0.001; ****p < 0.0001. (B) The anterior tibial muscle weight in each group of mice. One‐way ANOVA with Sidak's multiple comparisons test was used for statistical analysis. The p‐values are denoted with asterisks as follows: not significant (ns); *p < 0.05; **p < 0.01; ***p < 0.001; ****p < 0.0001. (C) The lean body weight changes of each group of mice. One‐way ANOVA with Sidak's multiple comparisons test was used for statistical analysis. P‐values are denoted with asterisks as follows: not significant (ns); *p < 0.05; **p < 0.01; ***p < 0.001; ****p < 0.0001. (D) Growth curves over 48 h of each group. (E) The typical picture of the hindlimb muscle from each group of mice. (F) The tumour weight curve of each group of mice. One‐way ANOVA with Sidak's multiple comparisons test was used for statistical analysis. The p‐values are denoted with asterisks as follows: not significant (ns); *p < 0.05; **p < 0.01; ***p < 0.001; ****p < 0.0001. [file JCSM-17-e70295-s003.pdf]

**A**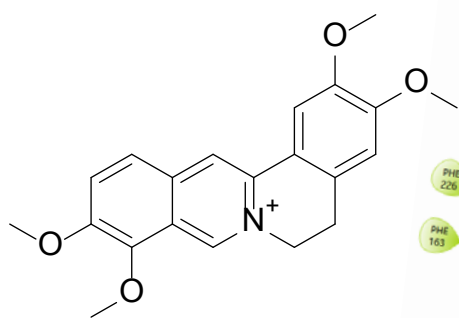

Palmatine

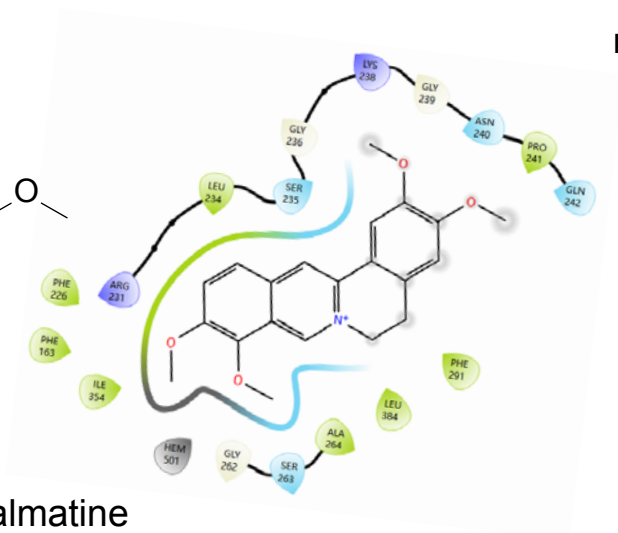**B**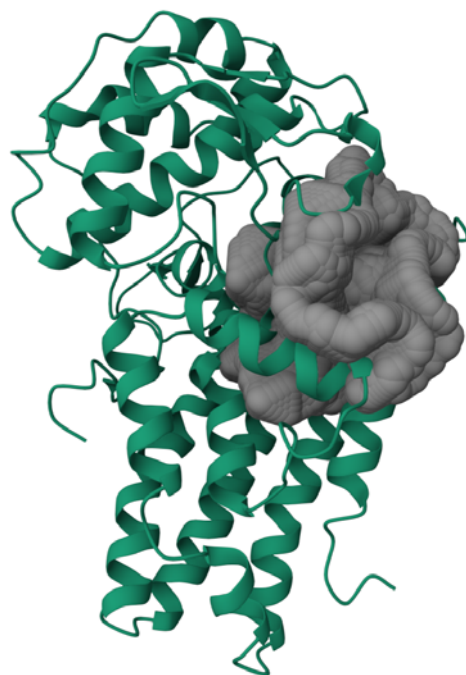**C**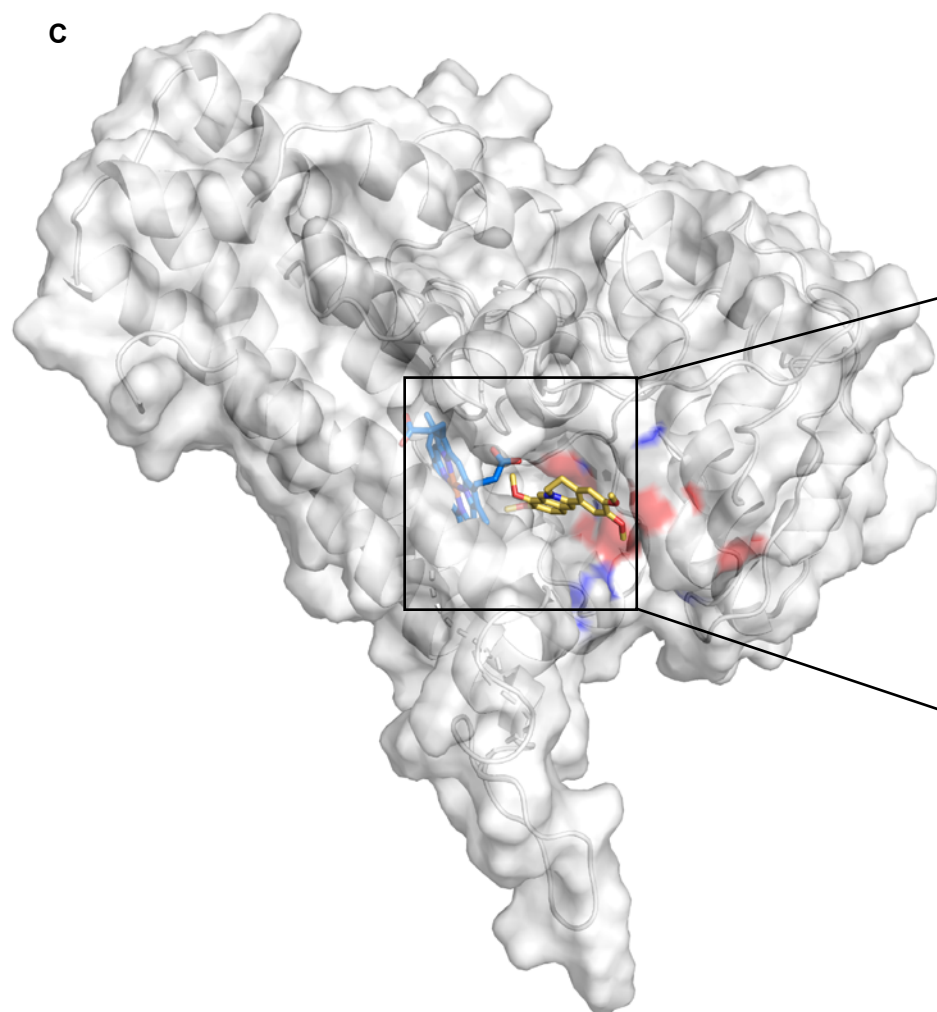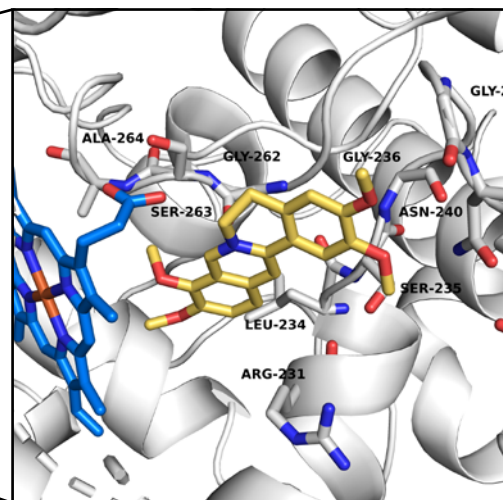

Supplement: Supplementary file 5 — Figure S5: Docking of palmatine with IDO1. (A) Chemical structure and 2D interaction diagram of palmatine. (B) The optimal binding pocket of IDO1 (PDB: 5EK2) obtained from the open‐source website Cavity Plus. Parameters: Box centre (Å) 21.25, −2.0, −22.25; box size (Å) 21.5, 23.0, 24.5; volume (Å3) 2360.25; surface area (Å2) 1613.00. (C) Molecular docking of IDO1 with palmatine. Palmatine exhibited a rigid quasi‐planar structure. After docking, the docking score is −4.867, and the MMGBSA binding free energy is −13.54 kcal/mol. Figures were generated using PYMOL 3.1.4.1. [file JCSM-17-e70295-s007.pdf]
