# Supplementary material for: Cancer IDO1‐Mediated Tryptophan–Kynurenine Metabolic Reprogramming to Drive Skeletal Muscle Atrophy and Cachexia Acceleration
Source: J Cachexia Sarcopenia Muscle. 2026 Apr 24;17(3):e70295. doi: 10.1002/jcsm.70295 (PMC13107547; doi:10.1002/jcsm.70295)
Supplement: Supplementary file 3 — Figure S3: Serum metabolome analysis and compound correlation studies. (A) Heatmap displaying pairwise correlation coefficients of detected metabolites across experimental groups. (B) PCA of metabolome data for each group. (C) Top 25 metabolites most strongly correlated with tryptophan levels. (D) The 25 metabolites most strongly correlated with kynurenine levels. (E) Violin plot comparing 5‐hydroxycanine uric acid and creatinine levels across the four experimental groups. [file JCSM-17-e70295-s010.pdf]

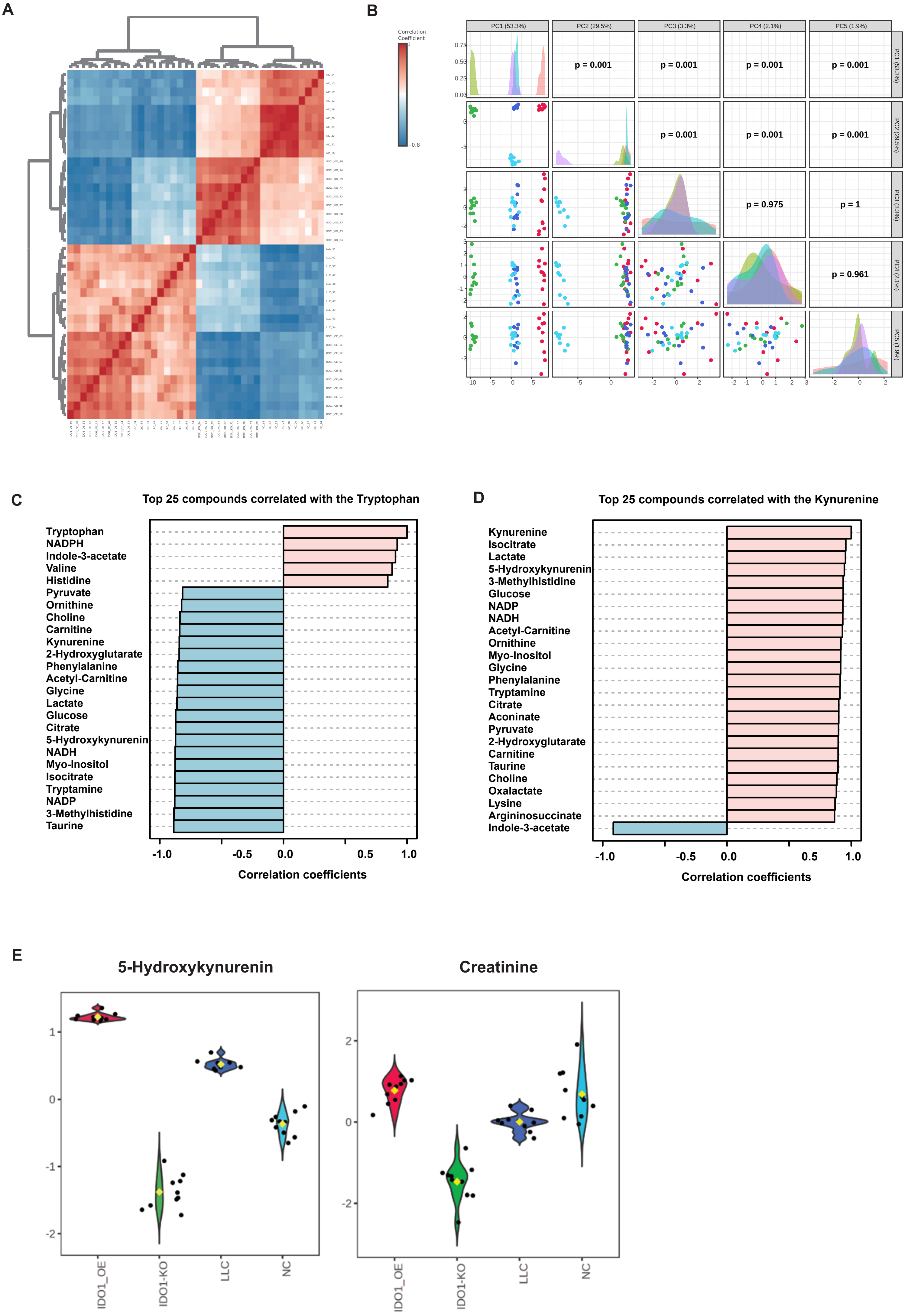

A
B
Correlation Coefficient
1
−0.8
PC1 (53.3%)
PC2 (29.5%)
PC3 (3.3%)
PC4 (2.1%)
PC5 (1.9%)
p = 0.001
p = 0.001
p = 0.001
p = 0.001
p = 0.001
p = 0.001
p = 0.001
p = 0.001
p = 0.001
p = 0.975
p = 1
p = 0.961
C
Top 25 compounds correlated with the Tryptophan
Tryptophan
NADPH
Indole-3-acetate
Valine
Histidine
Pyruvate
Ornithine
Choline
Carnitine
Kynurenine
2-Hydroxyglutarate
Phenylalanine
Acetyl-Carnitine
Glycine
Lactate
Glucose
Citrate
5-Hydroxykynurenin
NADH
Myo-Inositol
Isocitrate
Tryptamine
NADP
3-Methylhistidine
Taurine
-1.0
-0.5
0.0
0.5
1.0
Correlation coefficients
D
Top 25 compounds correlated with the Kynurenine
Kynurenine
Isocitrate
Lactate
5-Hydroxykynurenin
3-Methylhistidine
Glucose
NADP
NADH
Acetyl-Carnitine
Ornithine
Myo-Inositol
Glycine
Phenylalanine
Tryptamine
Citrate
Aconinate
Pyruvate
2-Hydroxyglutarate
Carnitine
Taurine
Choline
Oxalactate
Lysine
Argininosuccinate
Indole-3-acetate
-1.0
-0.5
0.0
0.5
1.0
Correlation coefficients
E
5-Hydroxykynurenin
Creatinine
IDO1_OE
IDO1-KO
LLC
NC
