# Supplementary material for: Cancer IDO1‐Mediated Tryptophan–Kynurenine Metabolic Reprogramming to Drive Skeletal Muscle Atrophy and Cachexia Acceleration
Source: J Cachexia Sarcopenia Muscle. 2026 Apr 24;17(3):e70295. doi: 10.1002/jcsm.70295 (PMC13107547; doi:10.1002/jcsm.70295)
Supplement: Supplementary file 6 — Table S1: The primers for the qPCR experiment. [file JCSM-17-e70295-s006.docx]

Table S1. Primer sequences for qPCR

| Primers | Forward | Reverse |
| --- | --- | --- |
| Ido1  MuRF1 | GGACTGCGACAAGGGCTTCTTC  AGTGTCCATGTCTGGAGGTCG | GTCTTGACGCTCTACTGCACTGG  ACTGGAGCACTCCTGCTTGTA |
| Atrogin1 | ACGTCGCAGCCAAGAAGAG | ATGGCGCTCCTTCGTACTTC |
| Myh | ACCCTTCCTGCTGCTGCTGAC | ACCCTTCCTGCTGCTGCTTTC |
| Myod | GGCGCCTTGCTCTGTCTTGGG | TTAGCCAGGGAATGACGCGTTAC |
| Myog | CTATGAGCGCGATGGAAGAGATG | CATTCACCTTCTTGAGCGTGGG |
| GAPDH | GGGTGTGAACCACGAGAAAT | CCTTCCACAATGCCAAAGTT |
