# Supplementary material for: Cancer IDO1‐Mediated Tryptophan–Kynurenine Metabolic Reprogramming to Drive Skeletal Muscle Atrophy and Cachexia Acceleration
Source: J Cachexia Sarcopenia Muscle. 2026 Apr 24;17(3):e70295. doi: 10.1002/jcsm.70295 (PMC13107547; doi:10.1002/jcsm.70295)
Supplement: Supplementary file 9 — Data S1: Supplementary references. [file JCSM-17-e70295-s008.docx]

Supplementary References

[S1] Cheng JJ, Ma XD, Ai GX, Yu QX, Chen XY, Yan F, et al. Palmatine Protects Against MSU-Induced Gouty Arthritis via Regulating the NF-κB/NLRP3 and Nrf2 Pathways. Drug Des Devel Ther. 2022;16:2119–32. doi:10.2147/dddt.S356307

[S2] Shi Z, Han L, Yang H, Lu G, Shi Z, Ma B. From traditional remedy to modern therapy: a comprehensive review of palmatine's multi-target mechanisms and ethnopharmacological potential. Front Pharmacol. 2025;16:1624353. doi:10.3389/fphar.2025.1624353
