# Supplementary material for: Cancer IDO1‐Mediated Tryptophan–Kynurenine Metabolic Reprogramming to Drive Skeletal Muscle Atrophy and Cachexia Acceleration
Source: J Cachexia Sarcopenia Muscle. 2026 Apr 24;17(3):e70295. doi: 10.1002/jcsm.70295 (PMC13107547; doi:10.1002/jcsm.70295)

Figure2D

MuRF1
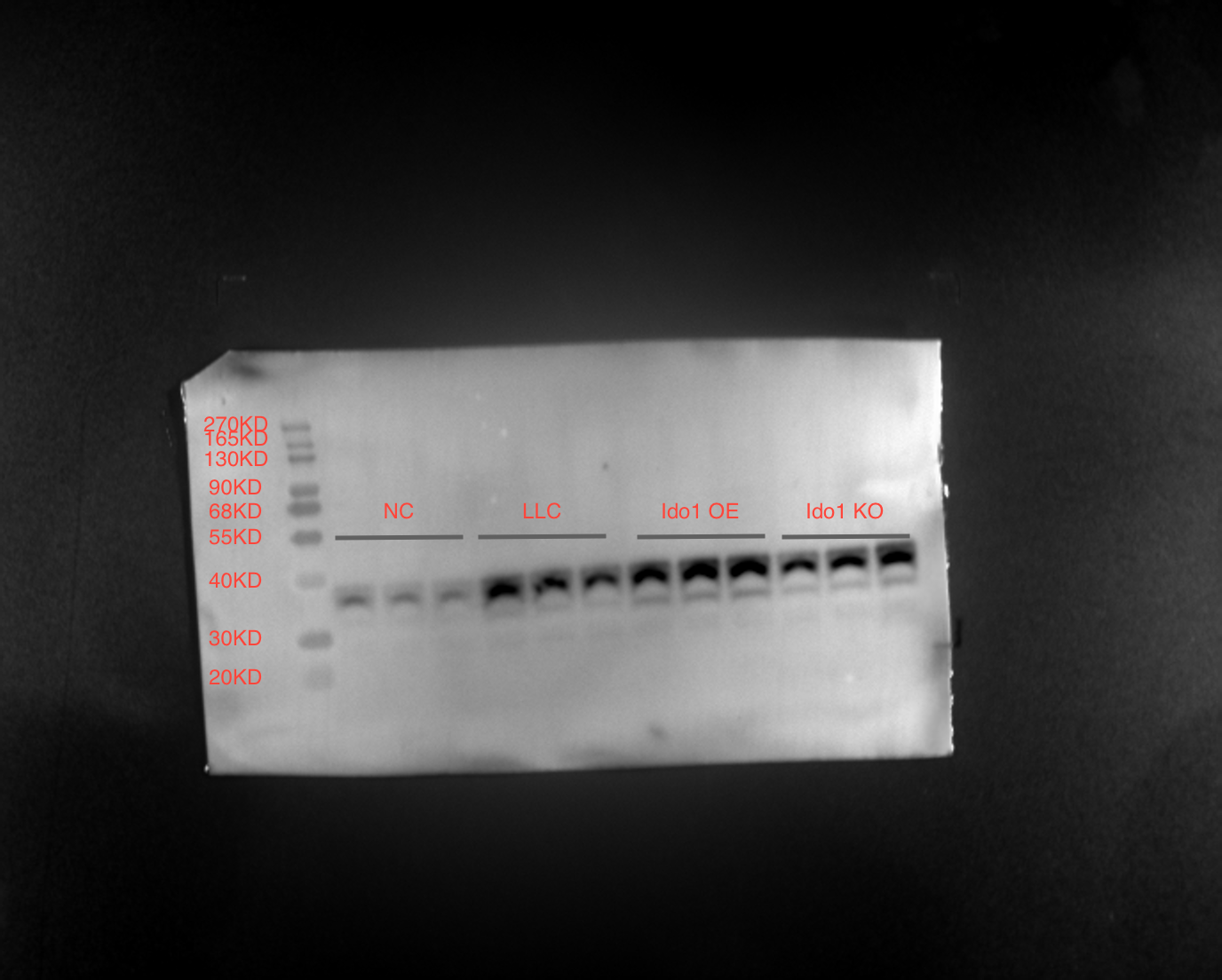


GAPDH
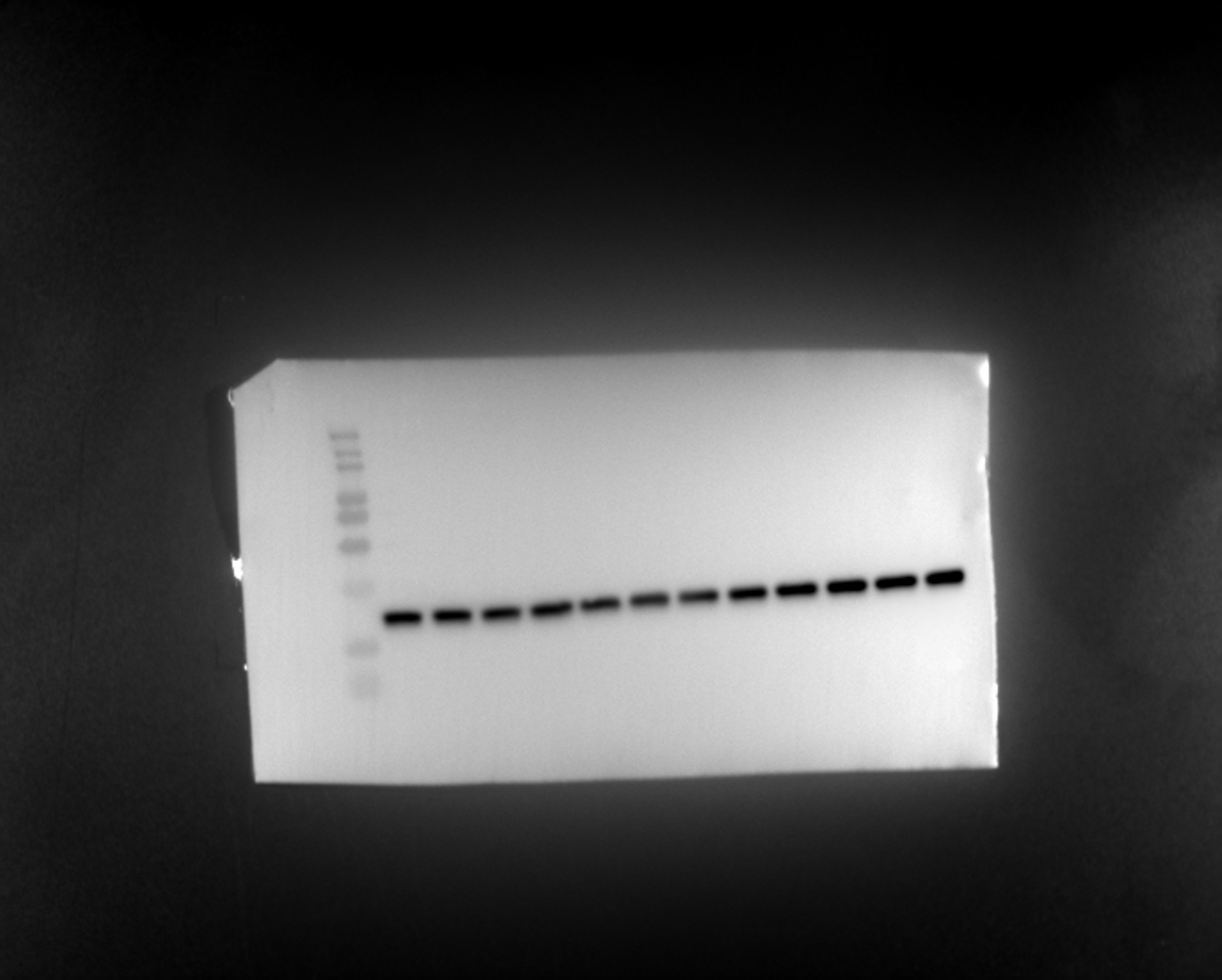


Atrogin1
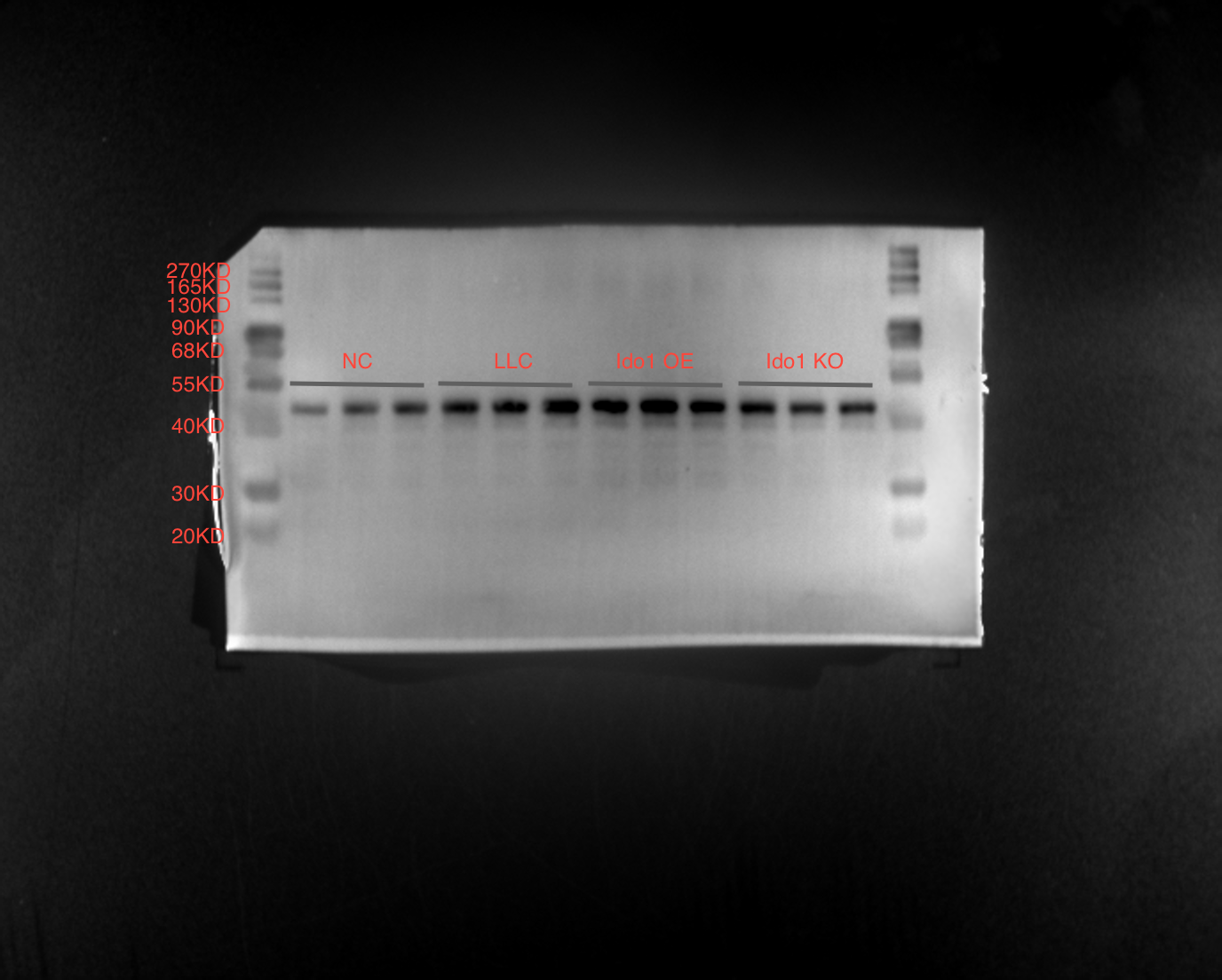


GAPDH
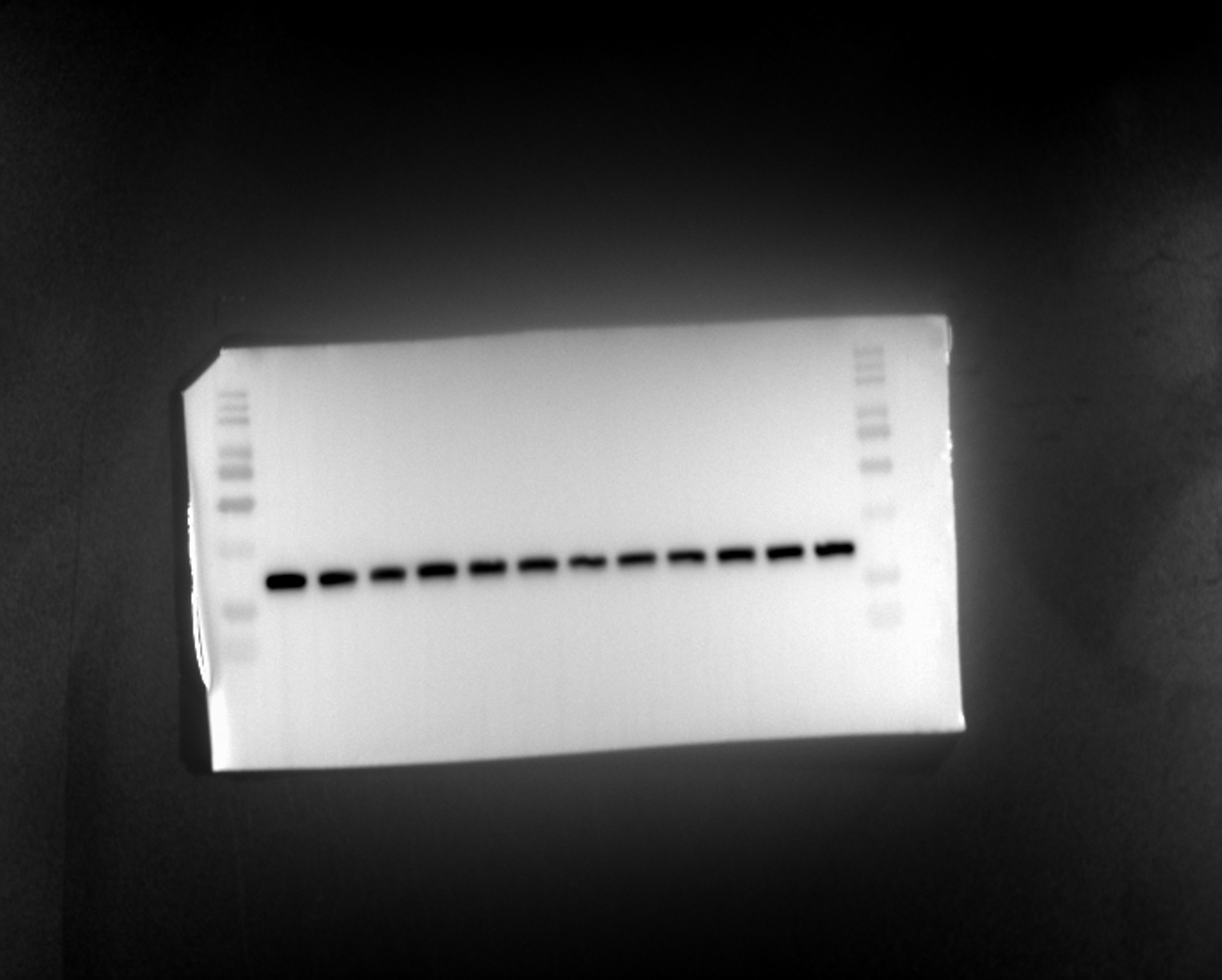


Figure3A

P70s6K
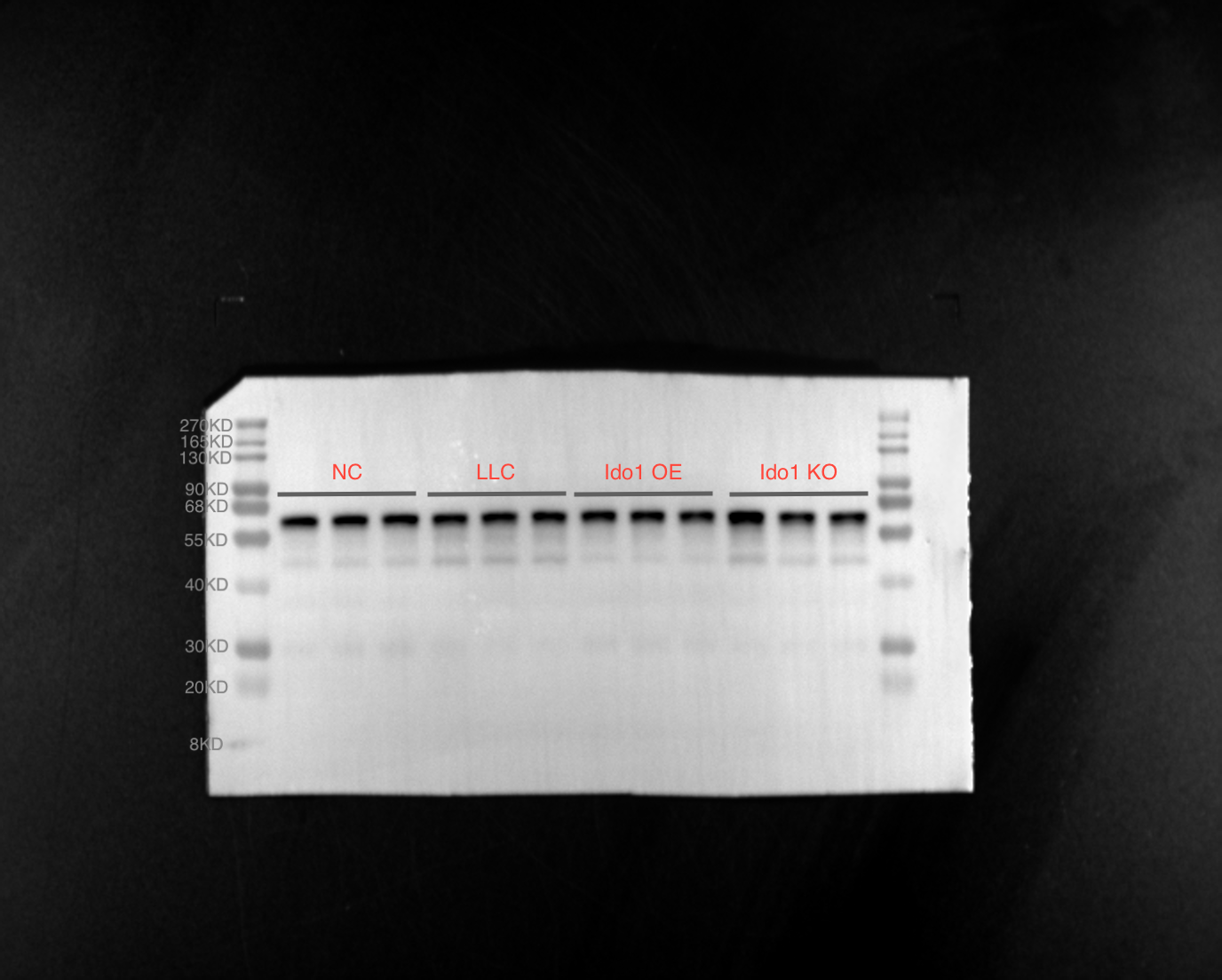


GAPDH
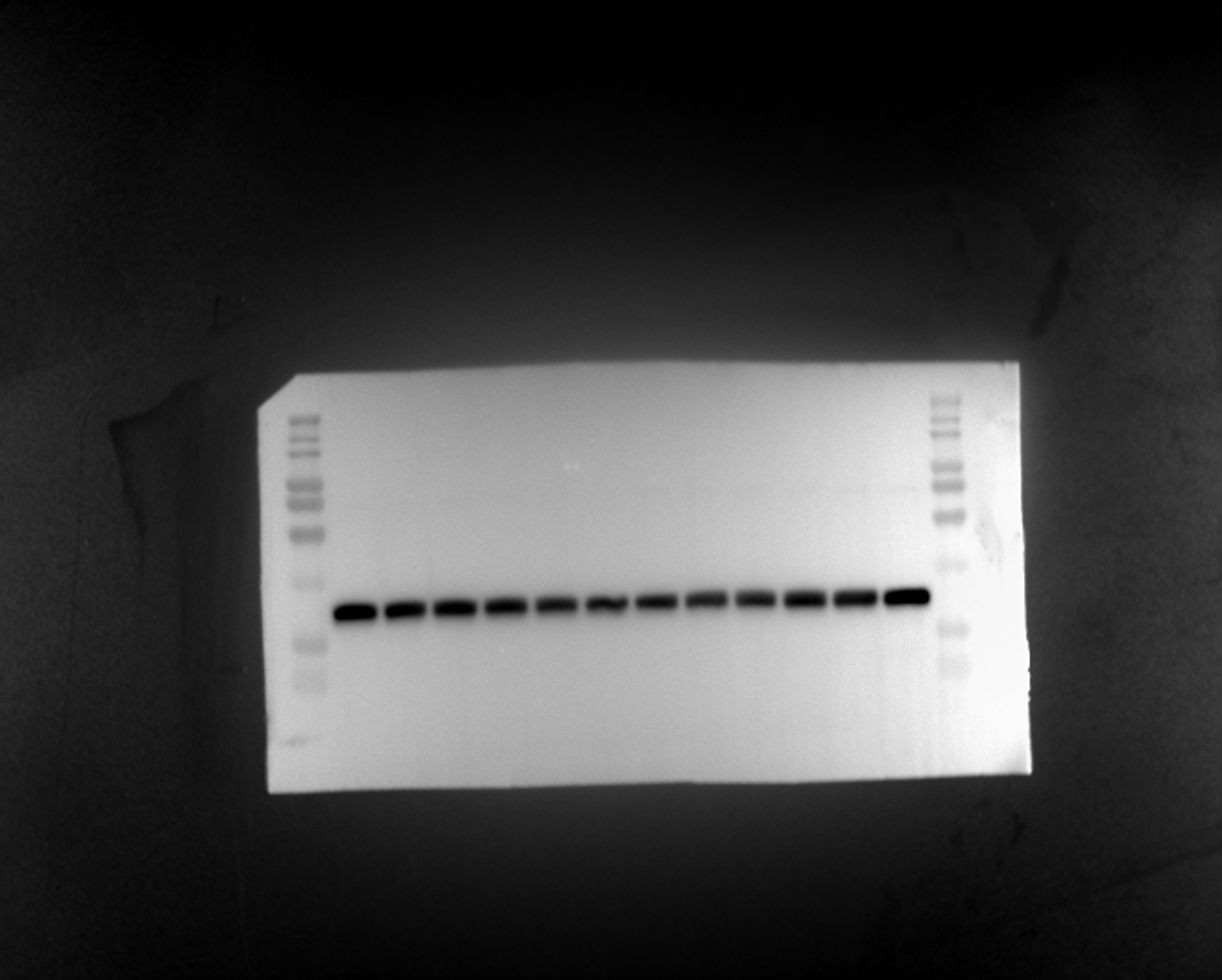


p-P70s6K
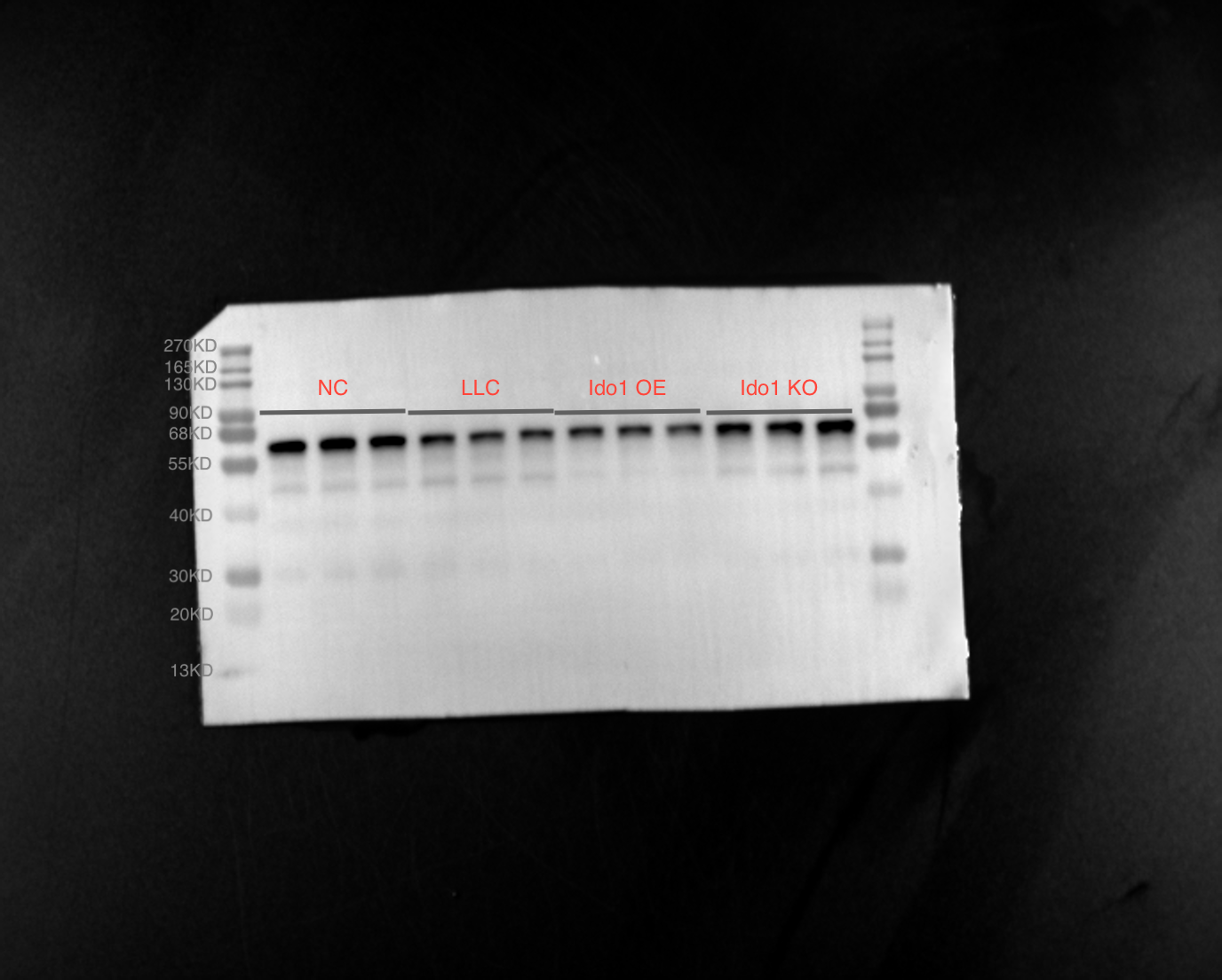


GAPDH
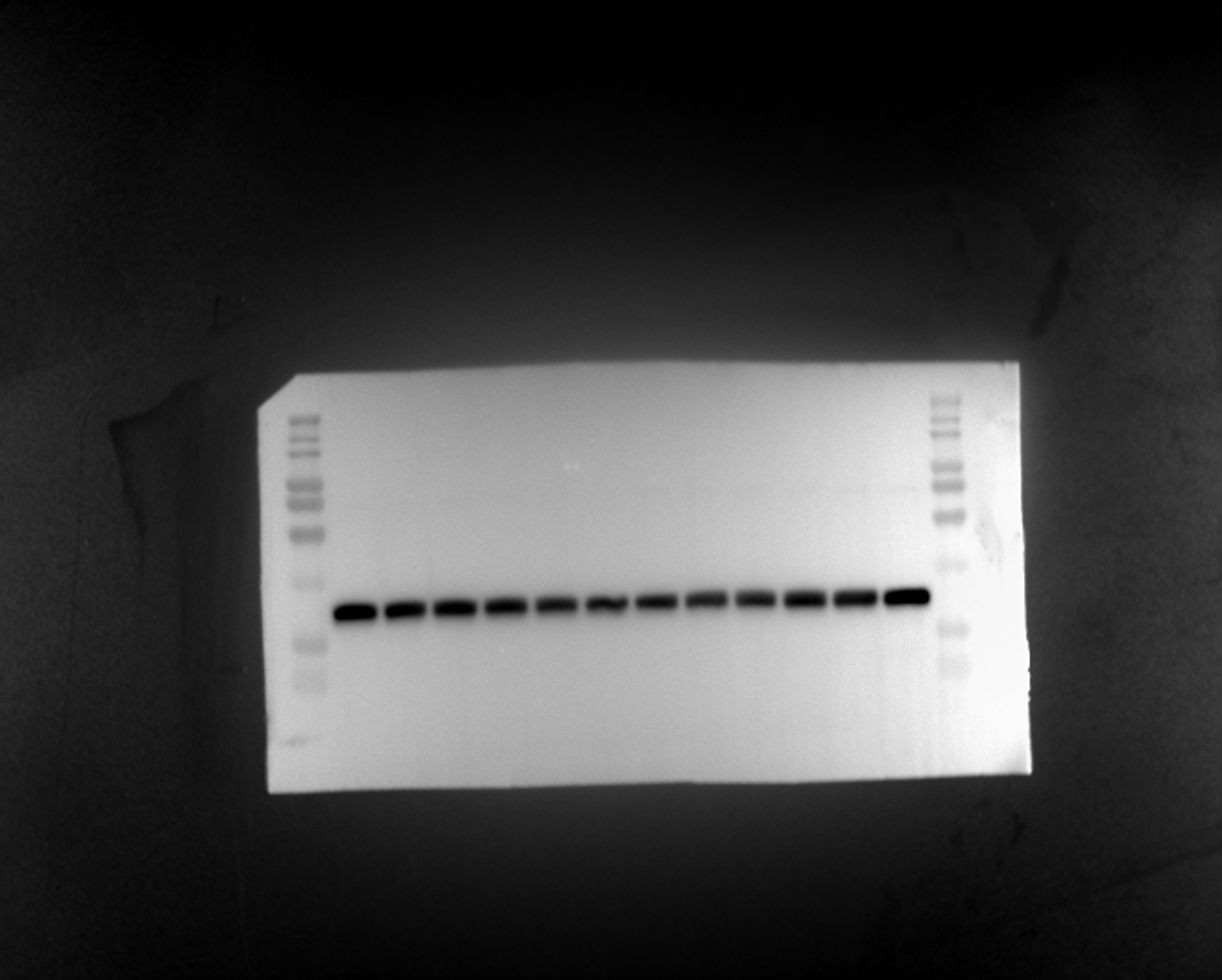


4EBP1
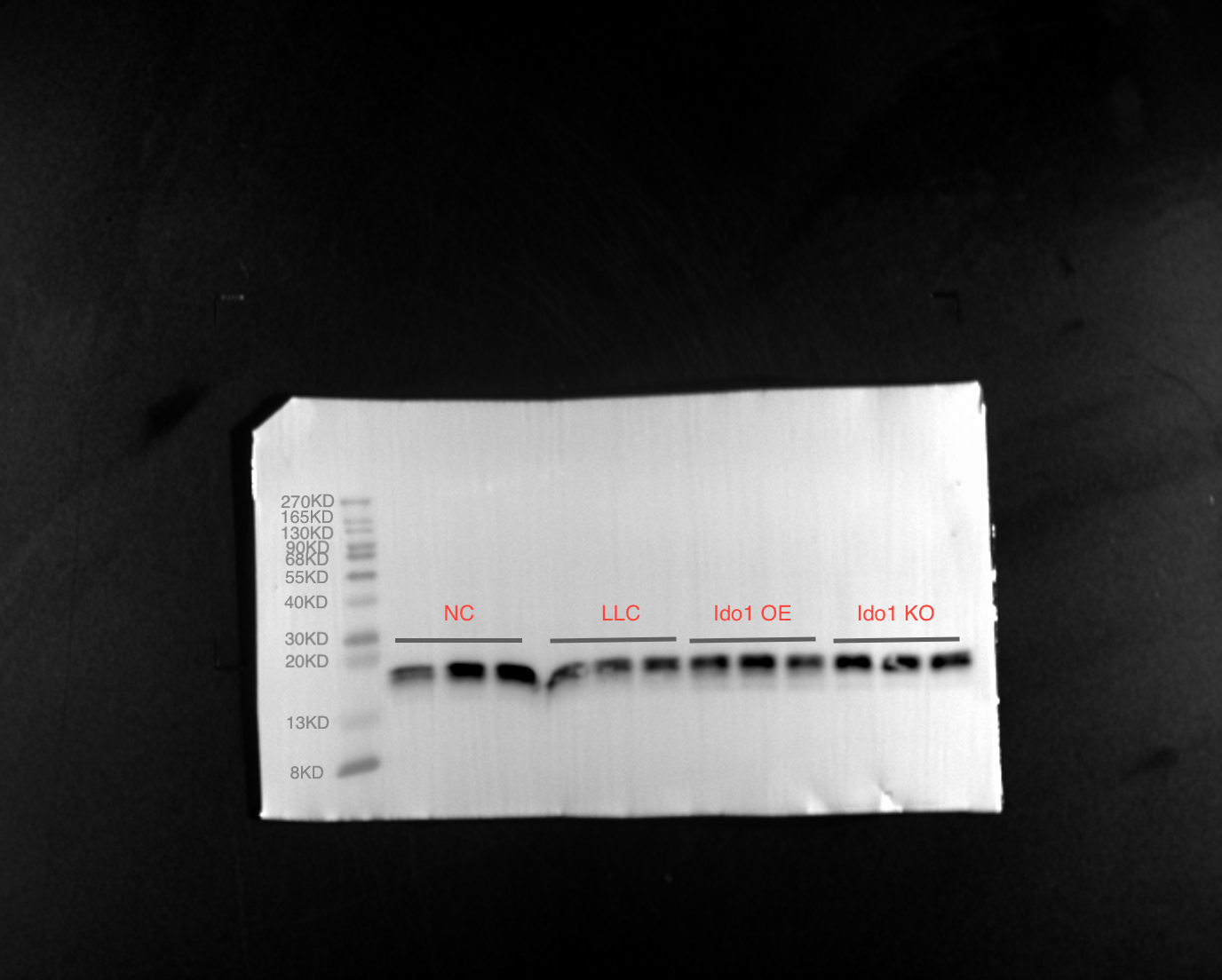


GAPDH
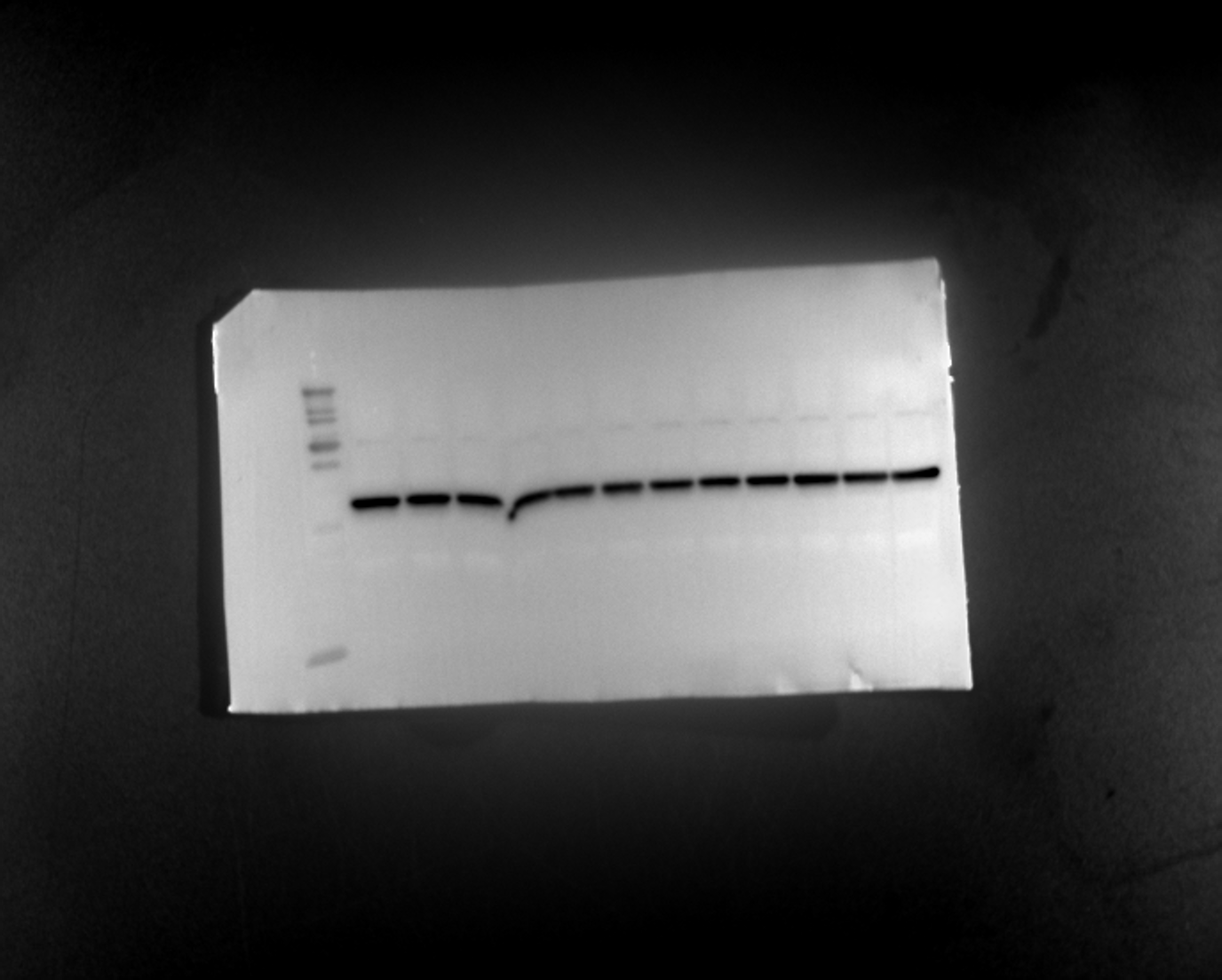


p-4EBP1
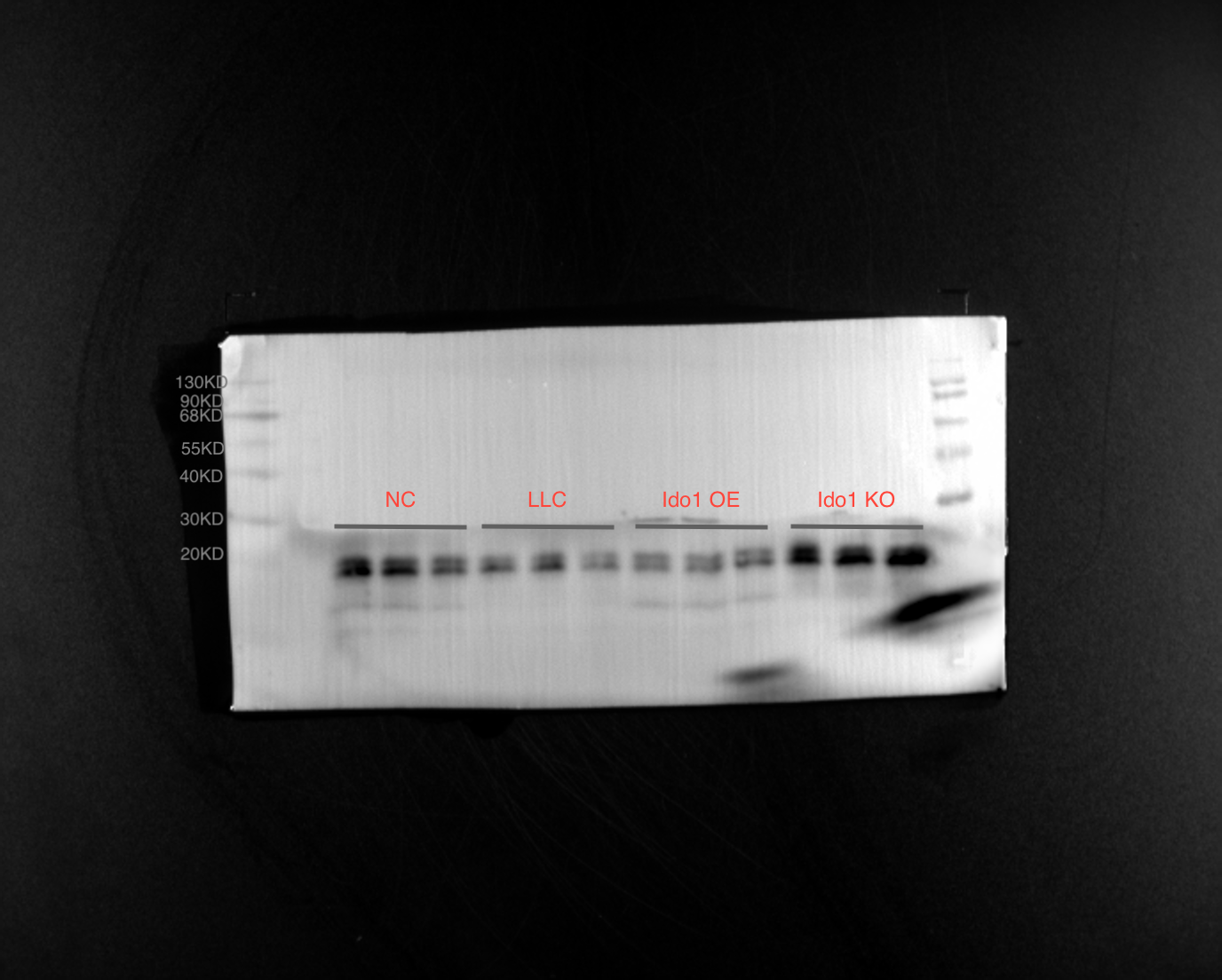


GAPDH
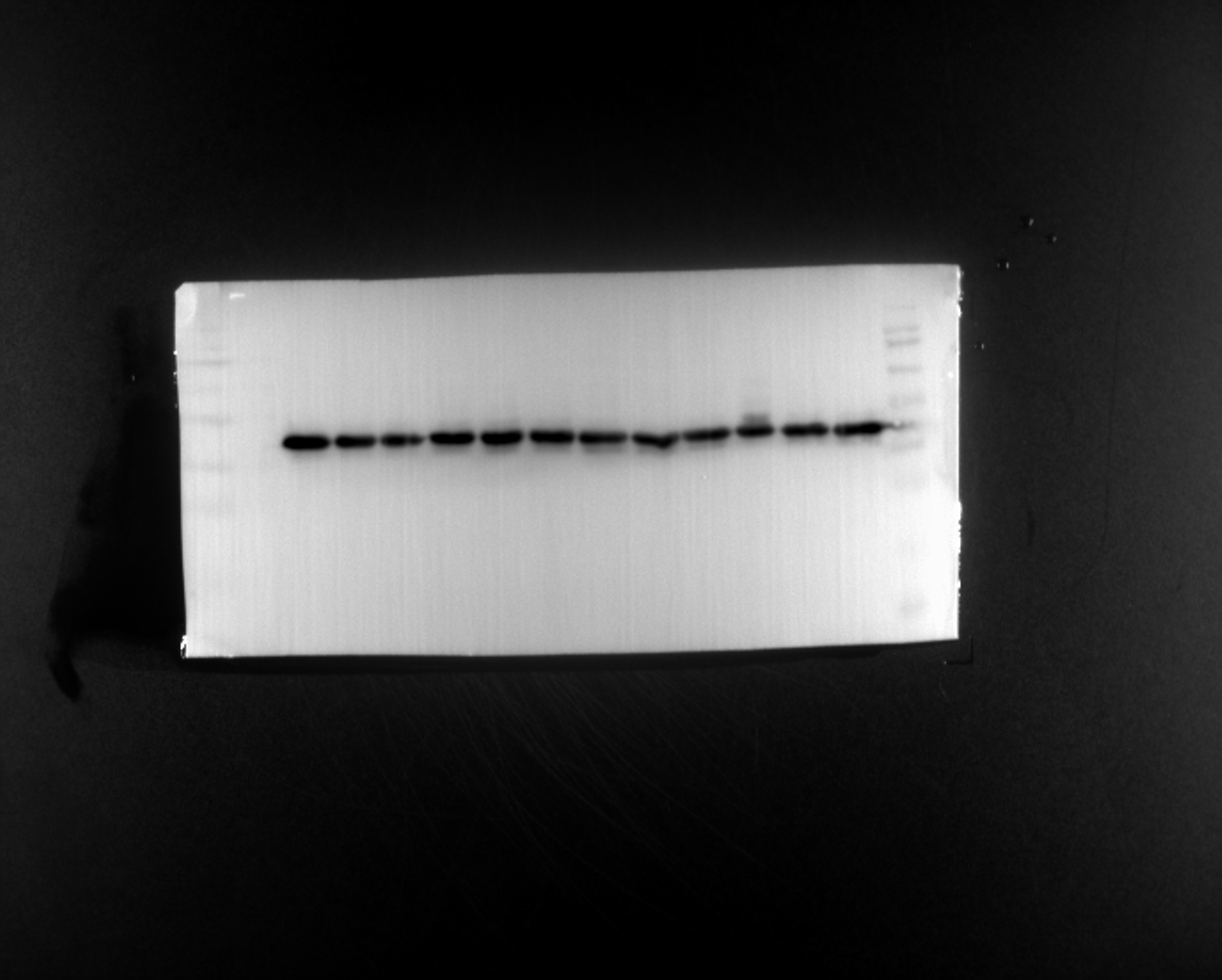


mTOR
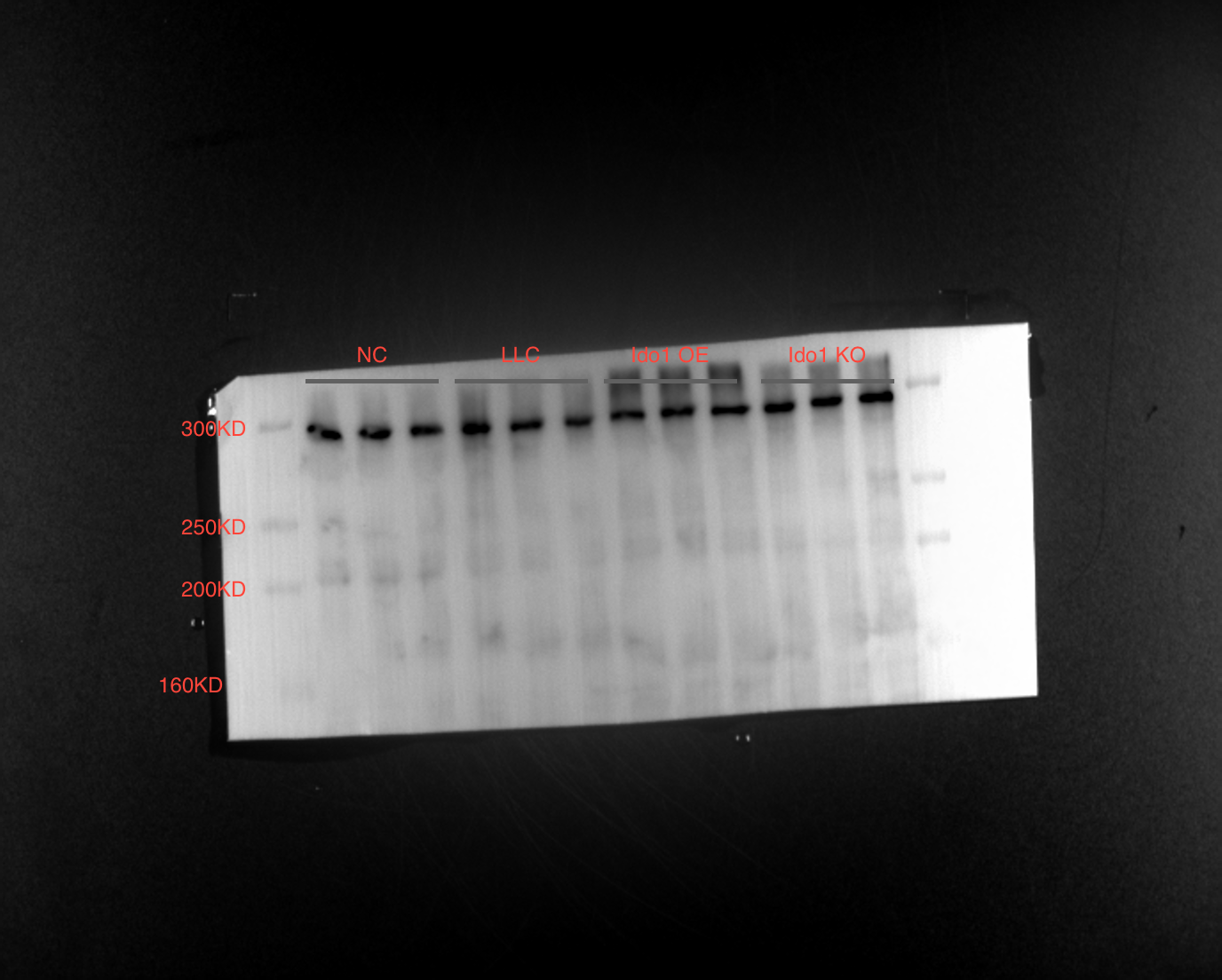


Vinculin
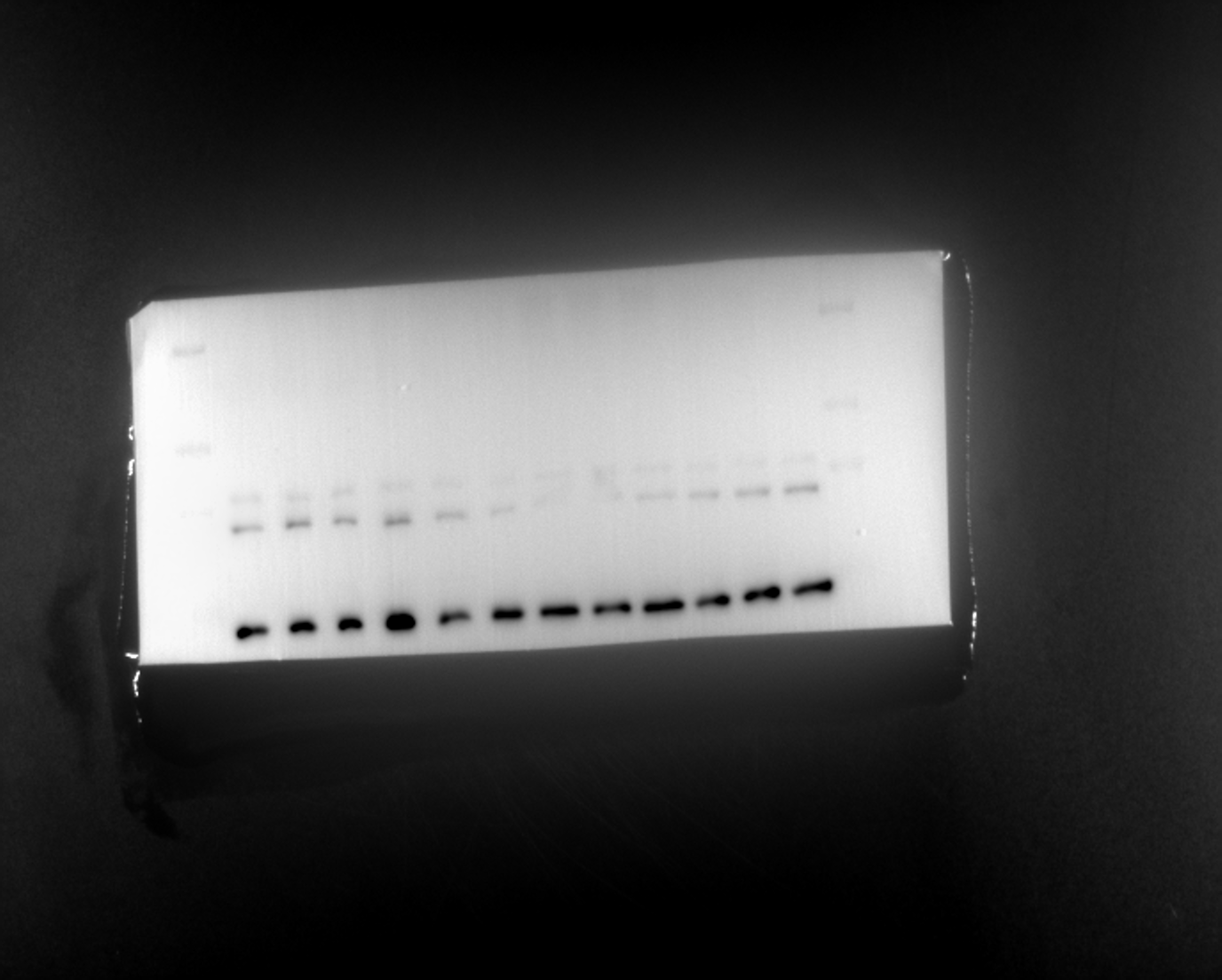


p-mTOR
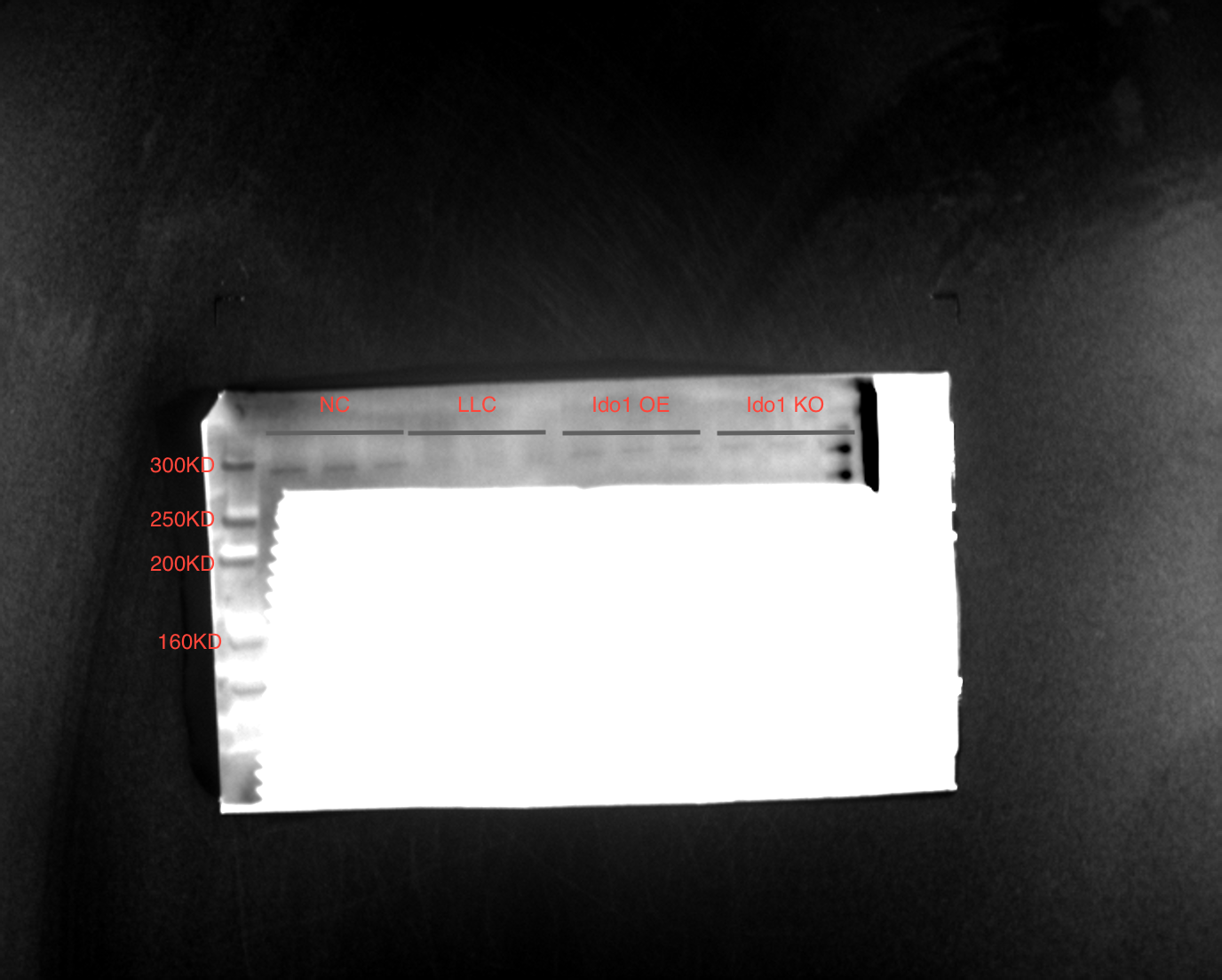


Vinculin
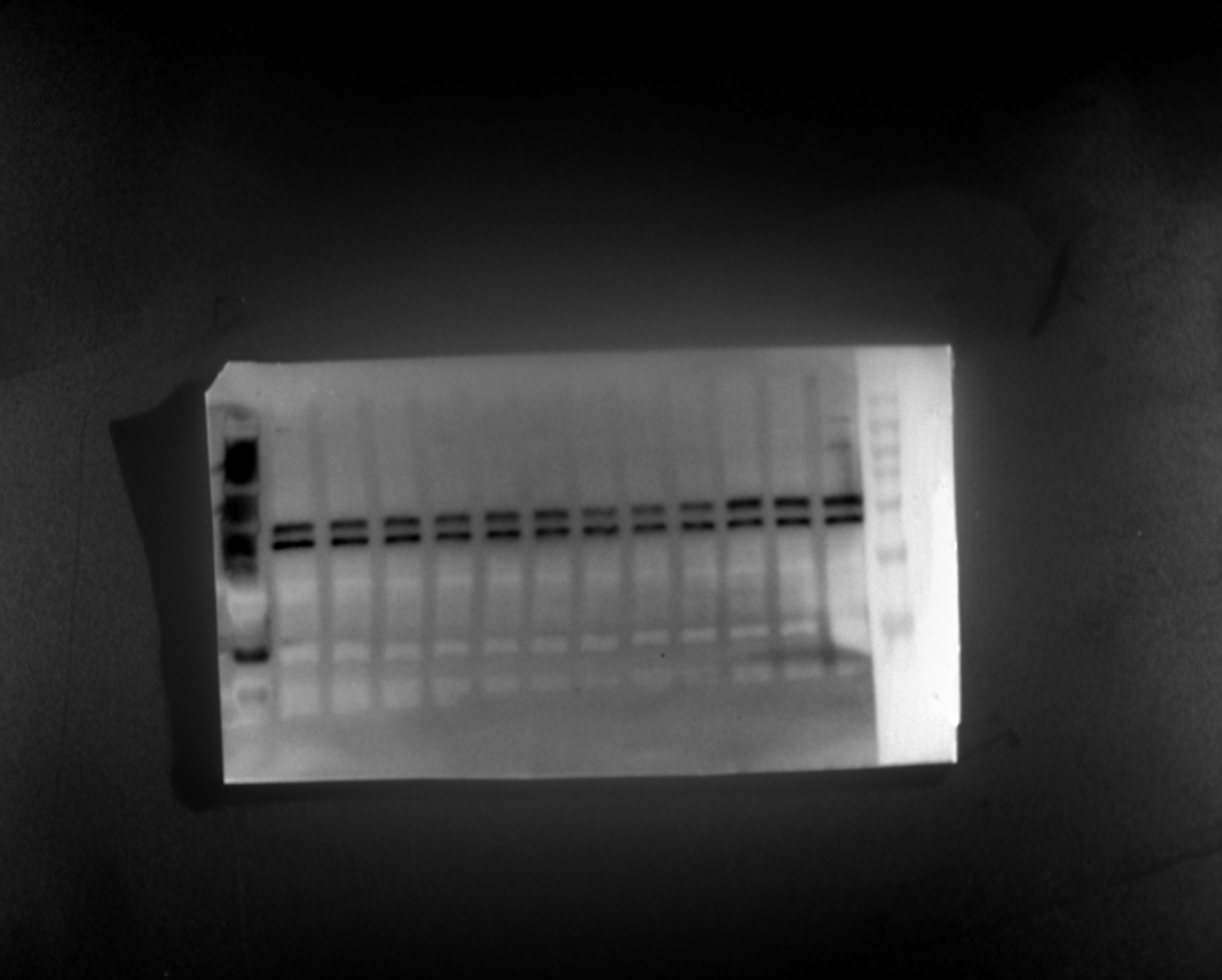


Figure8A

MuRF1
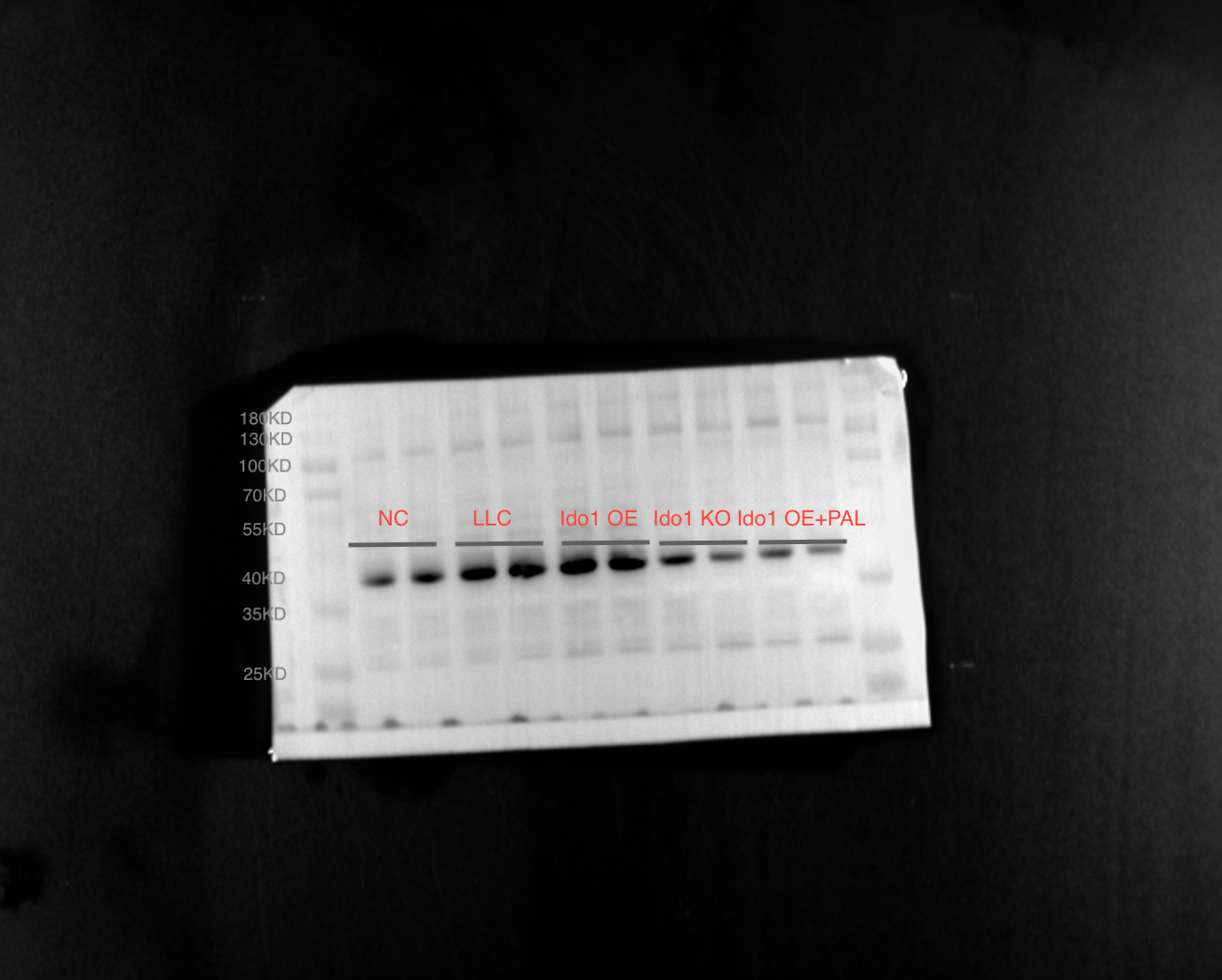


GAPDH
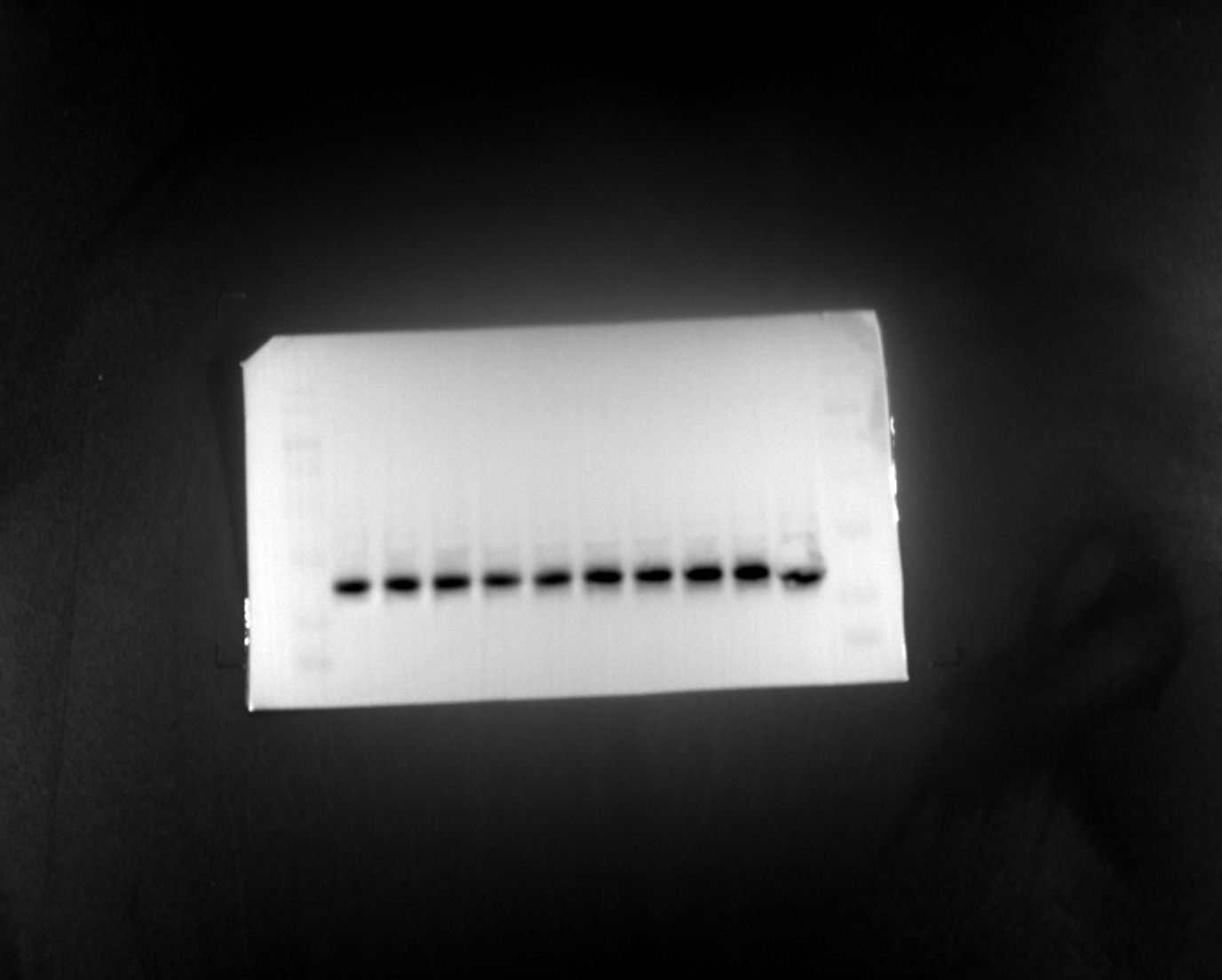


Atrogin1
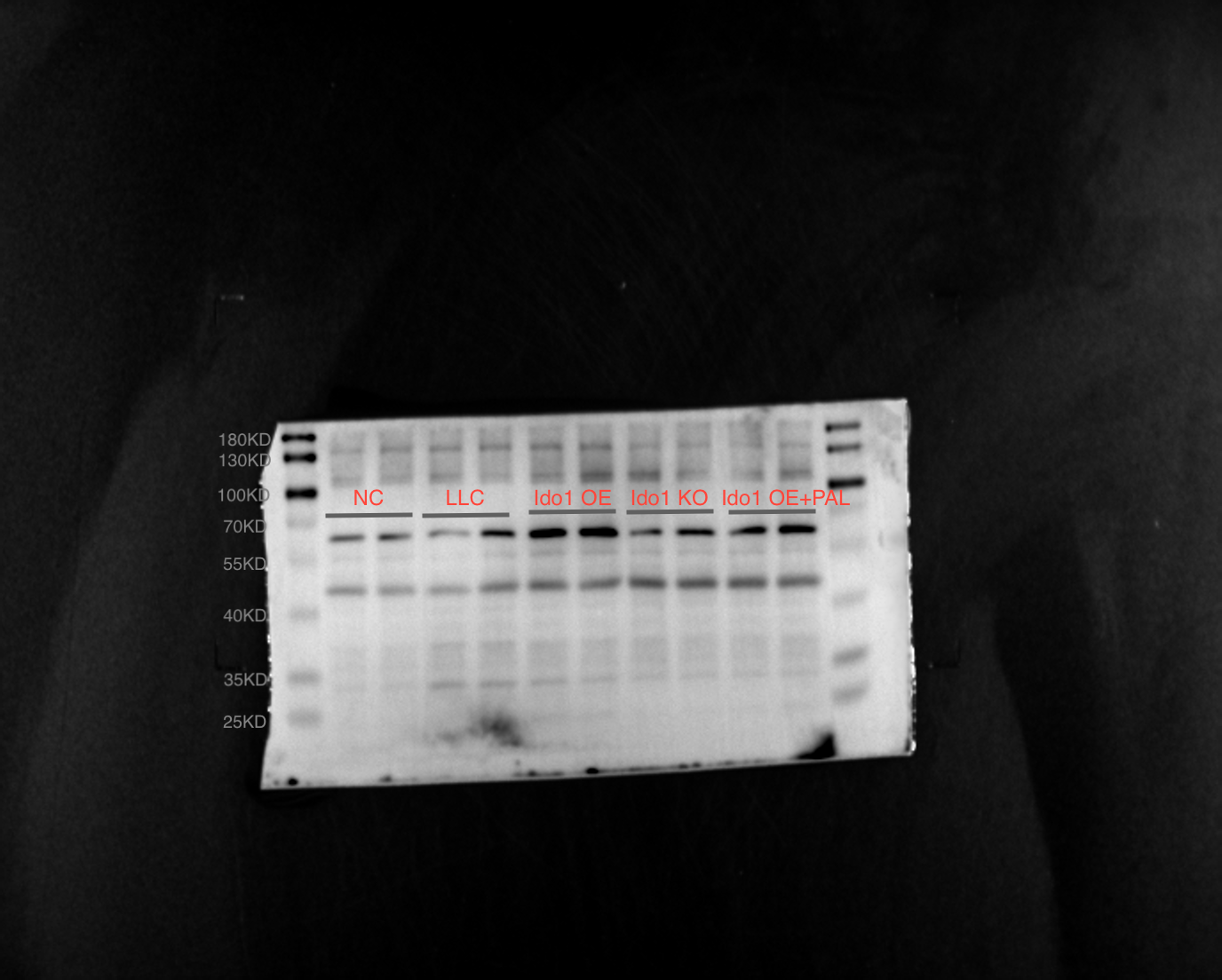


GAPDH
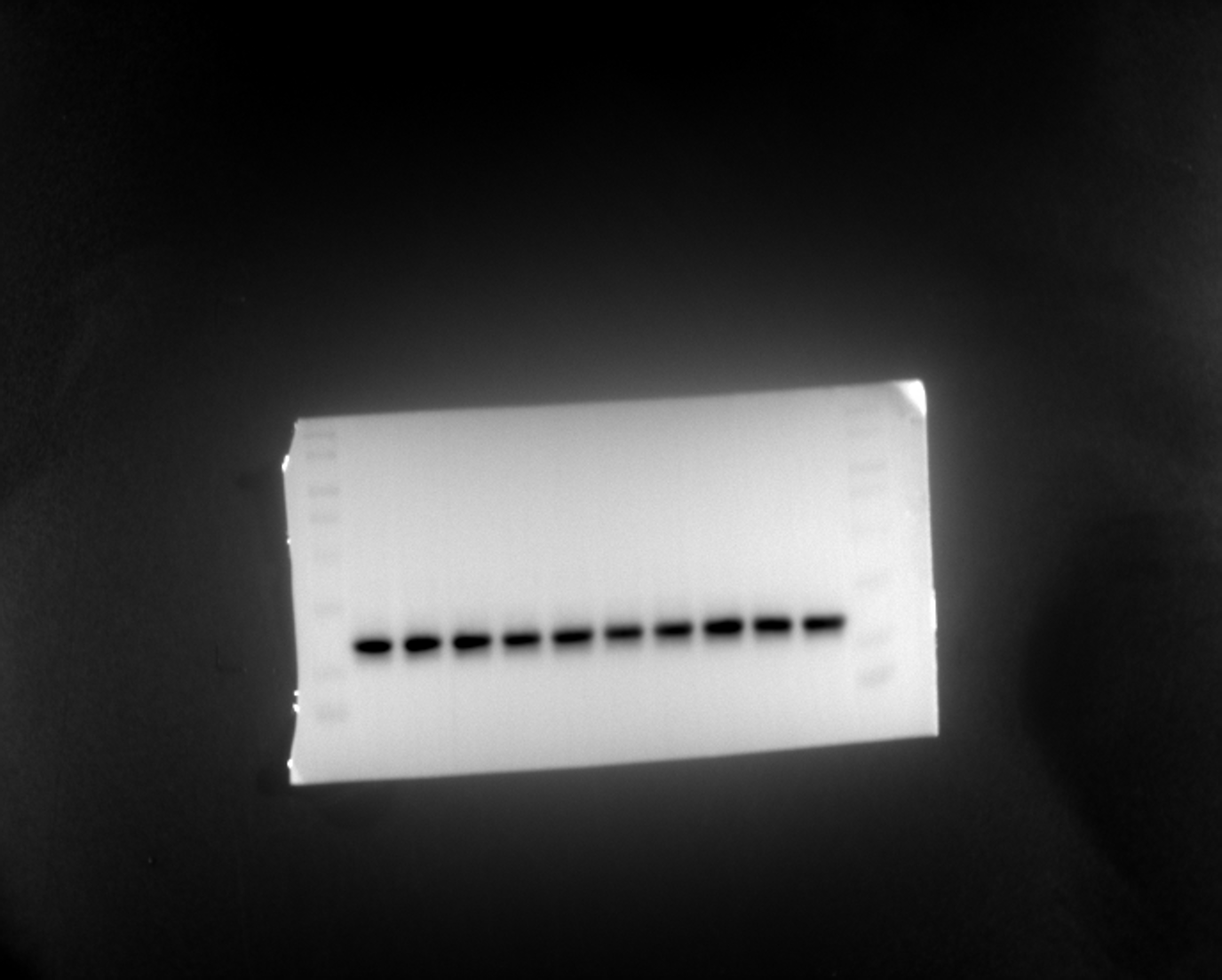


Figure 8D

mTOR
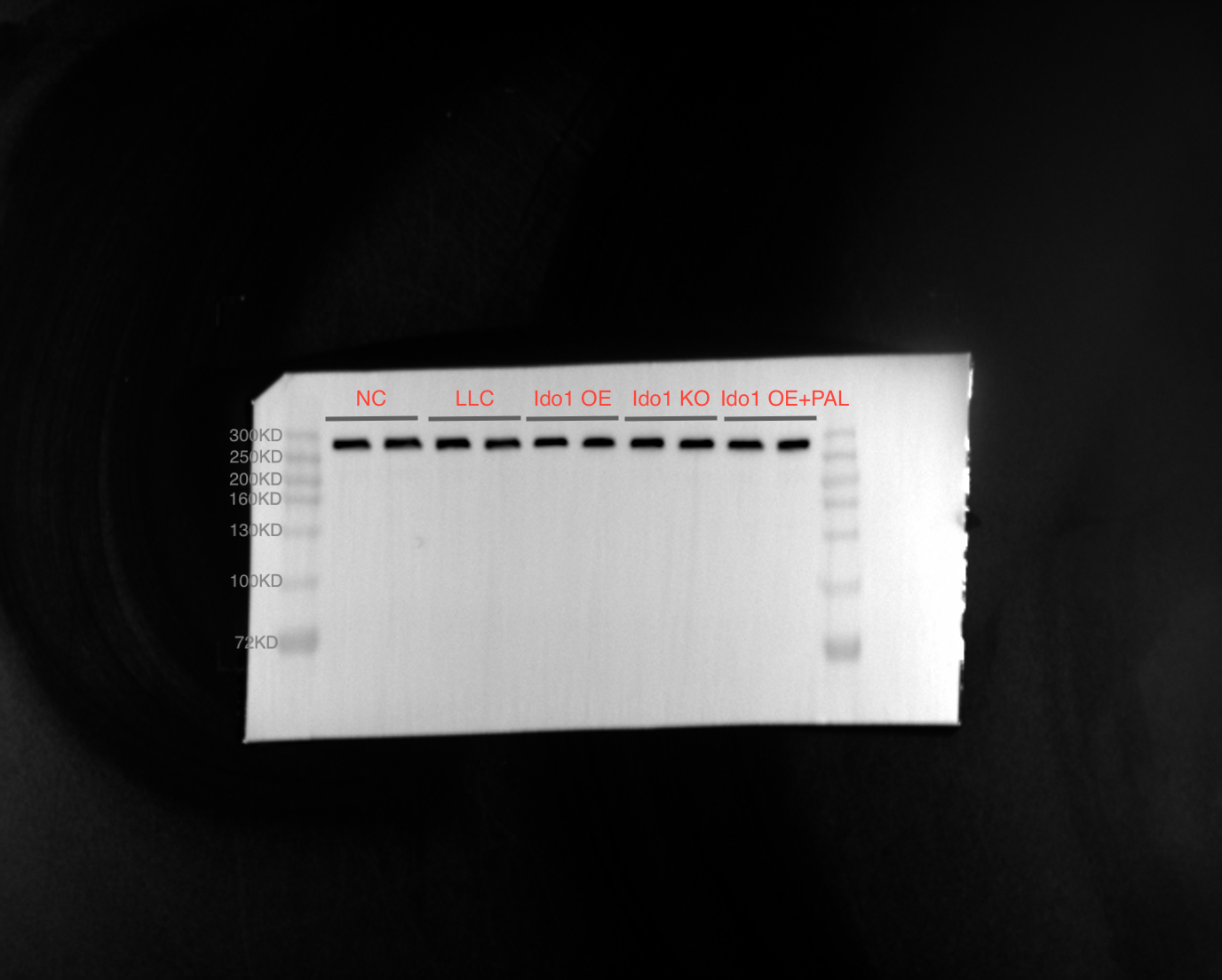


Vinculin
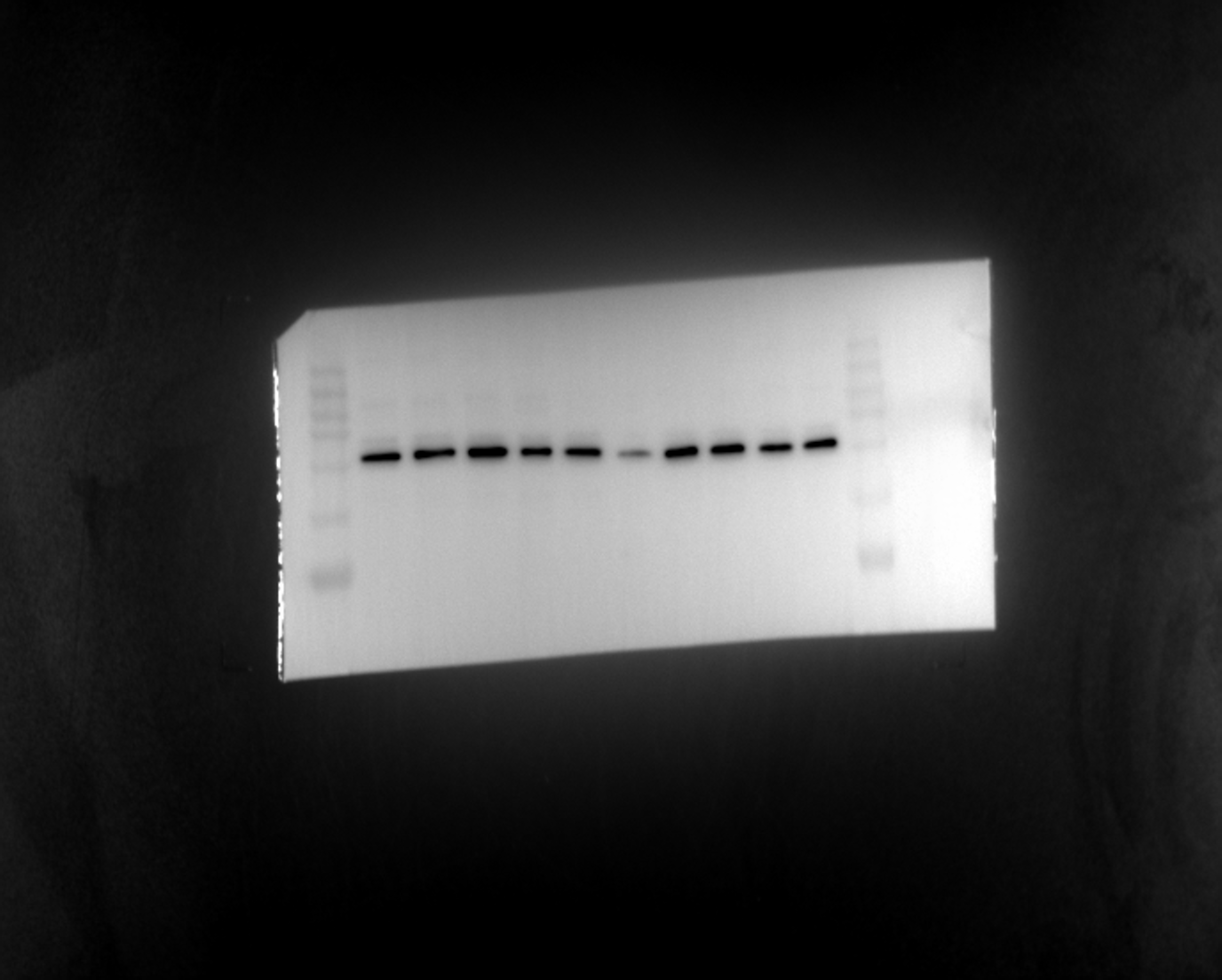


p-mTOR
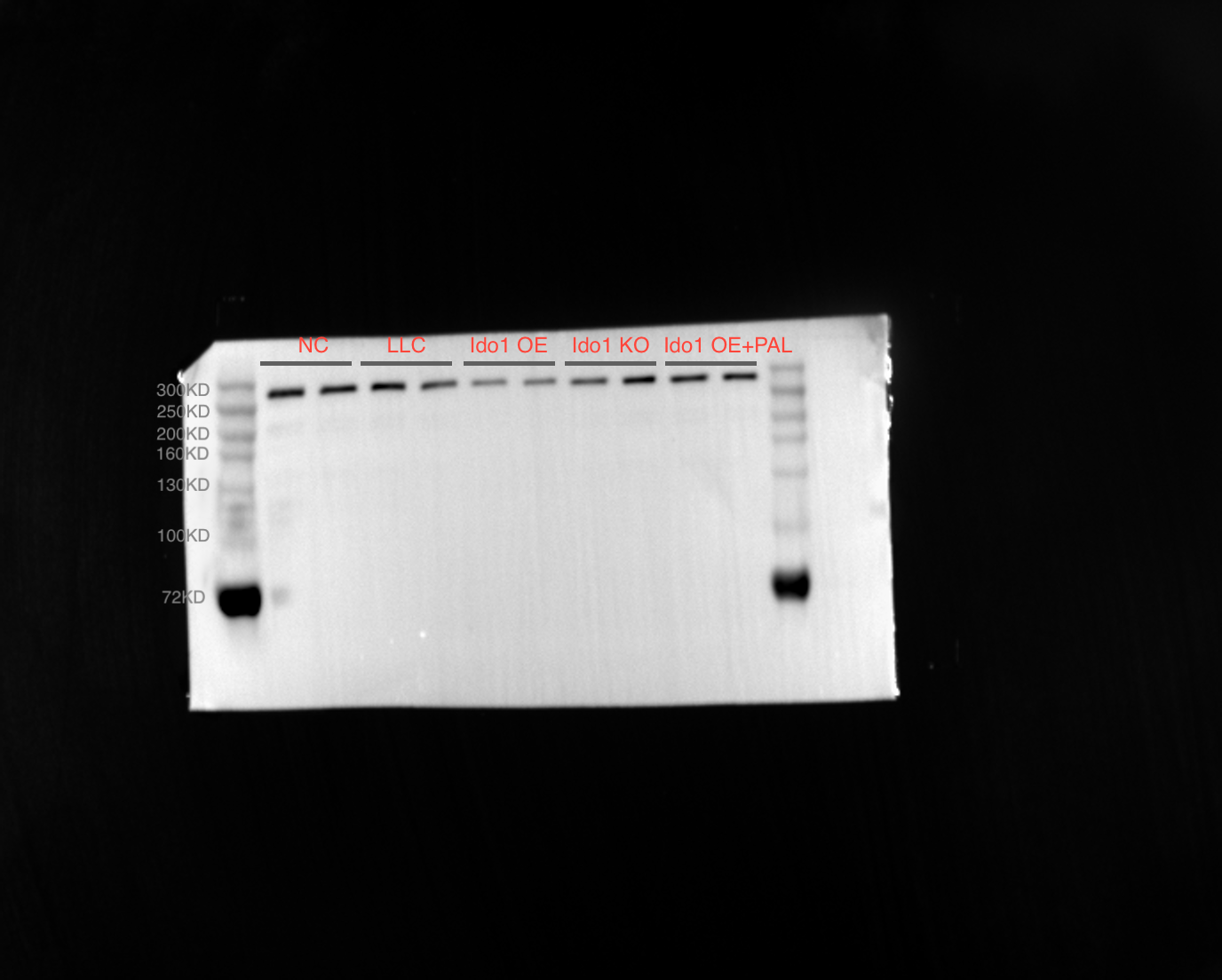


Vinculin
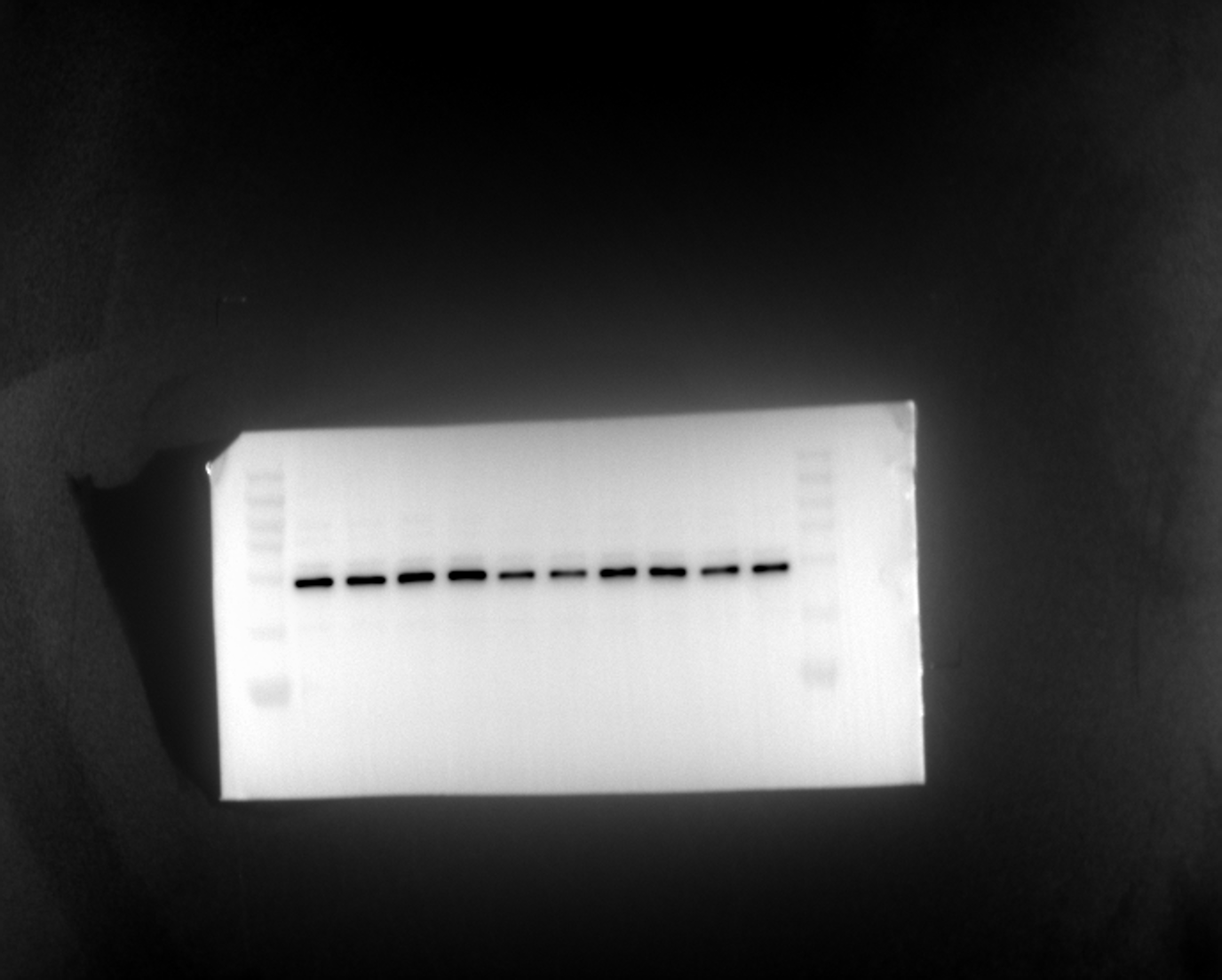


4EBP1
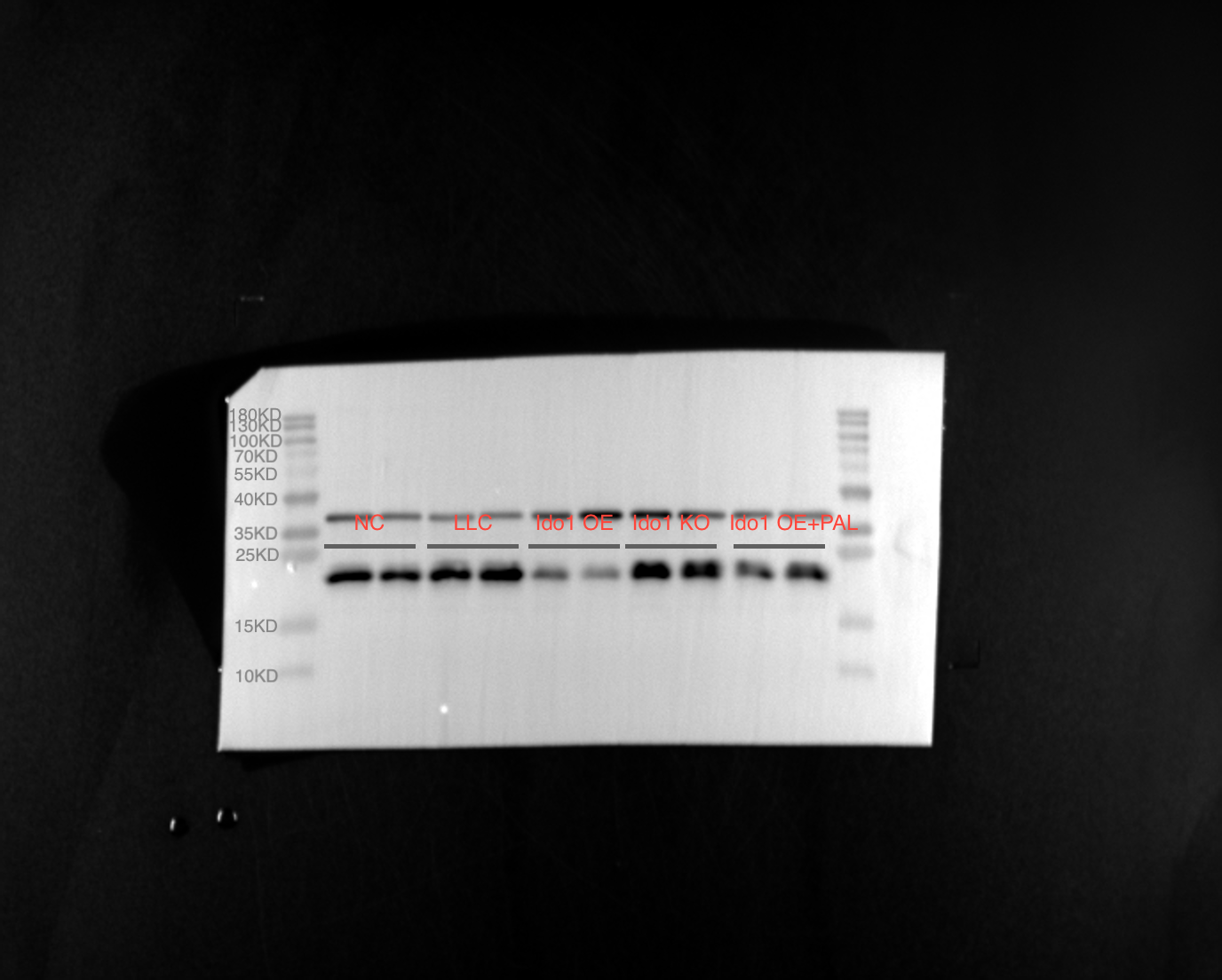


GAPDH
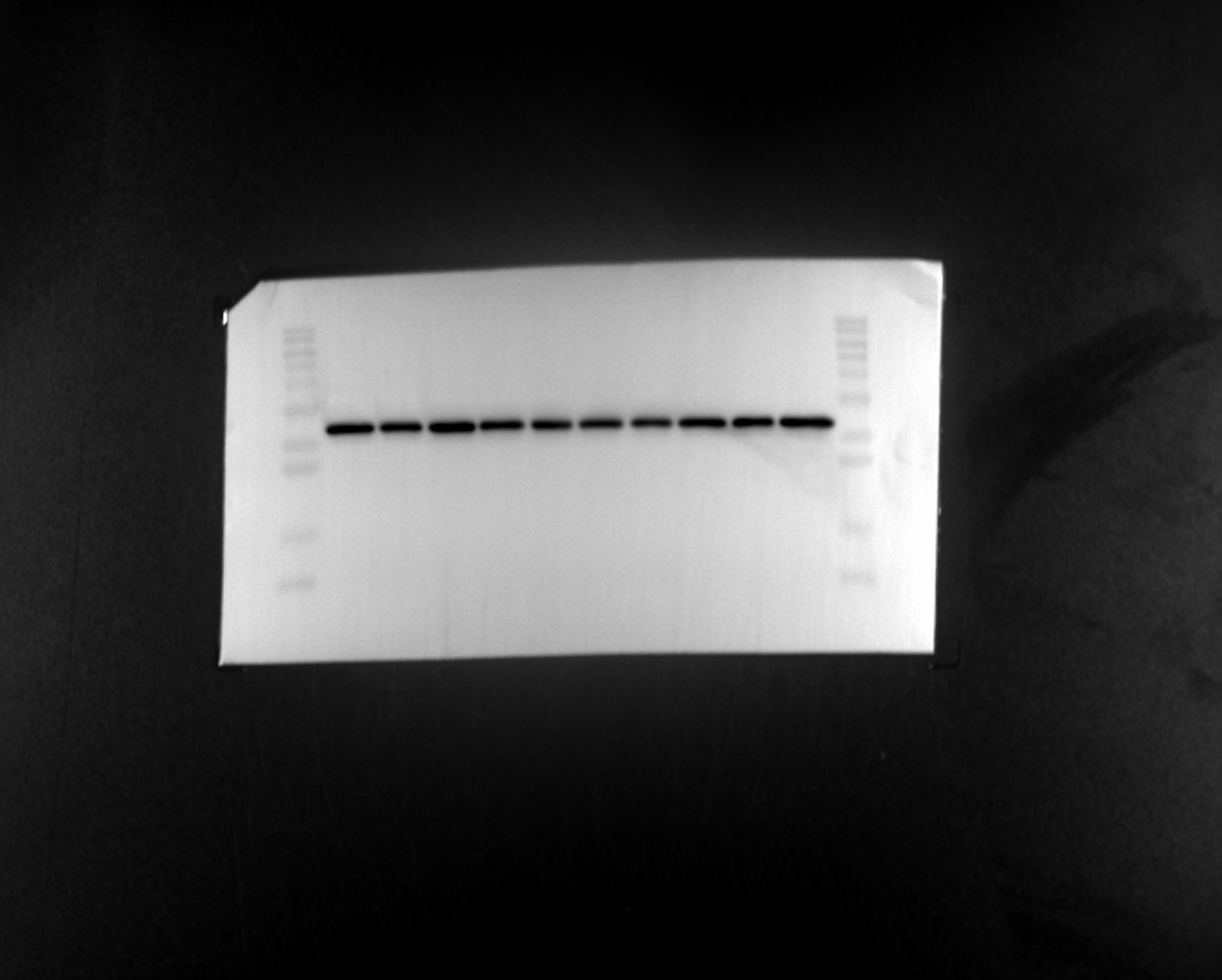


p-4EBP1
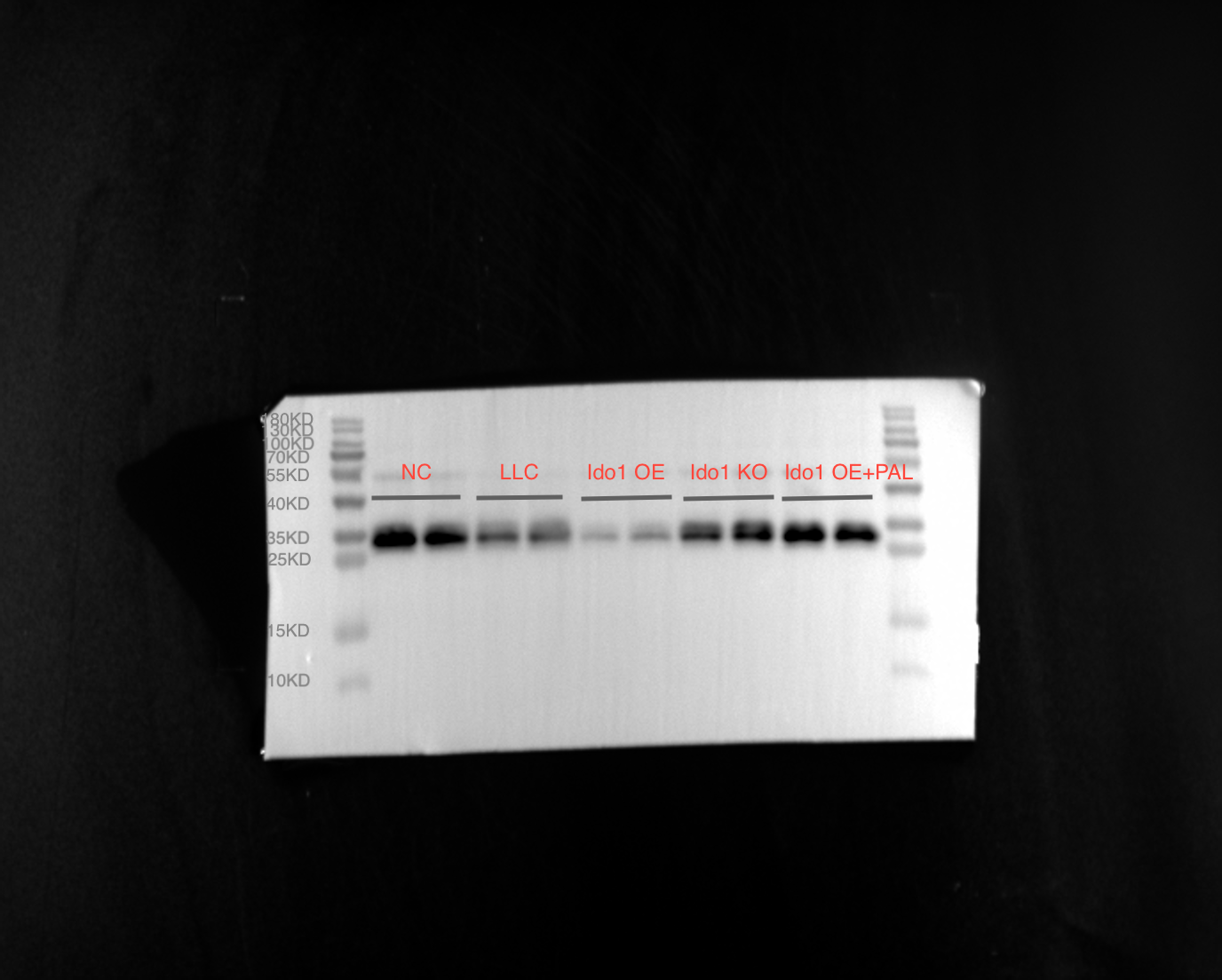


GAPDH
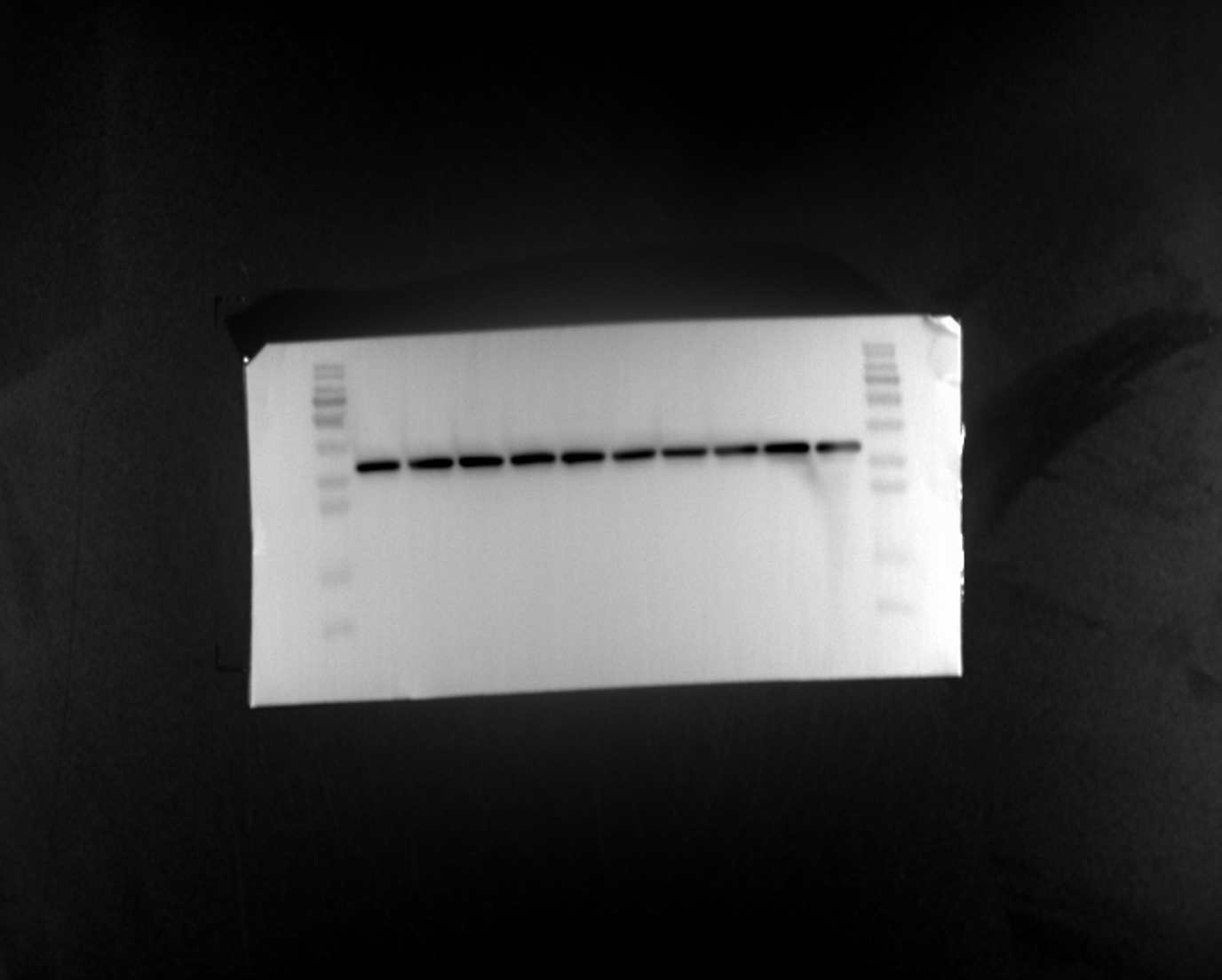


P70s6K
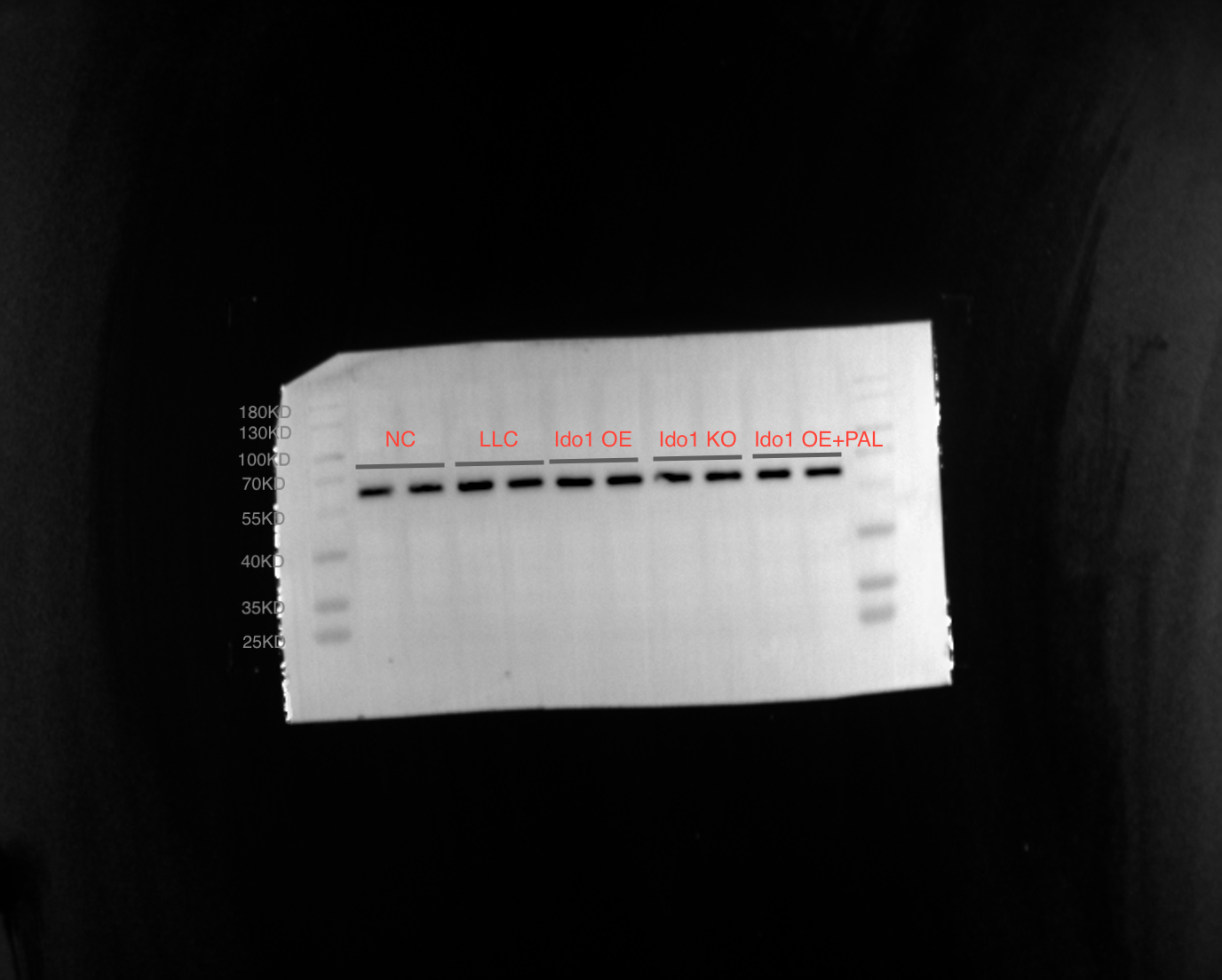


GAPDH
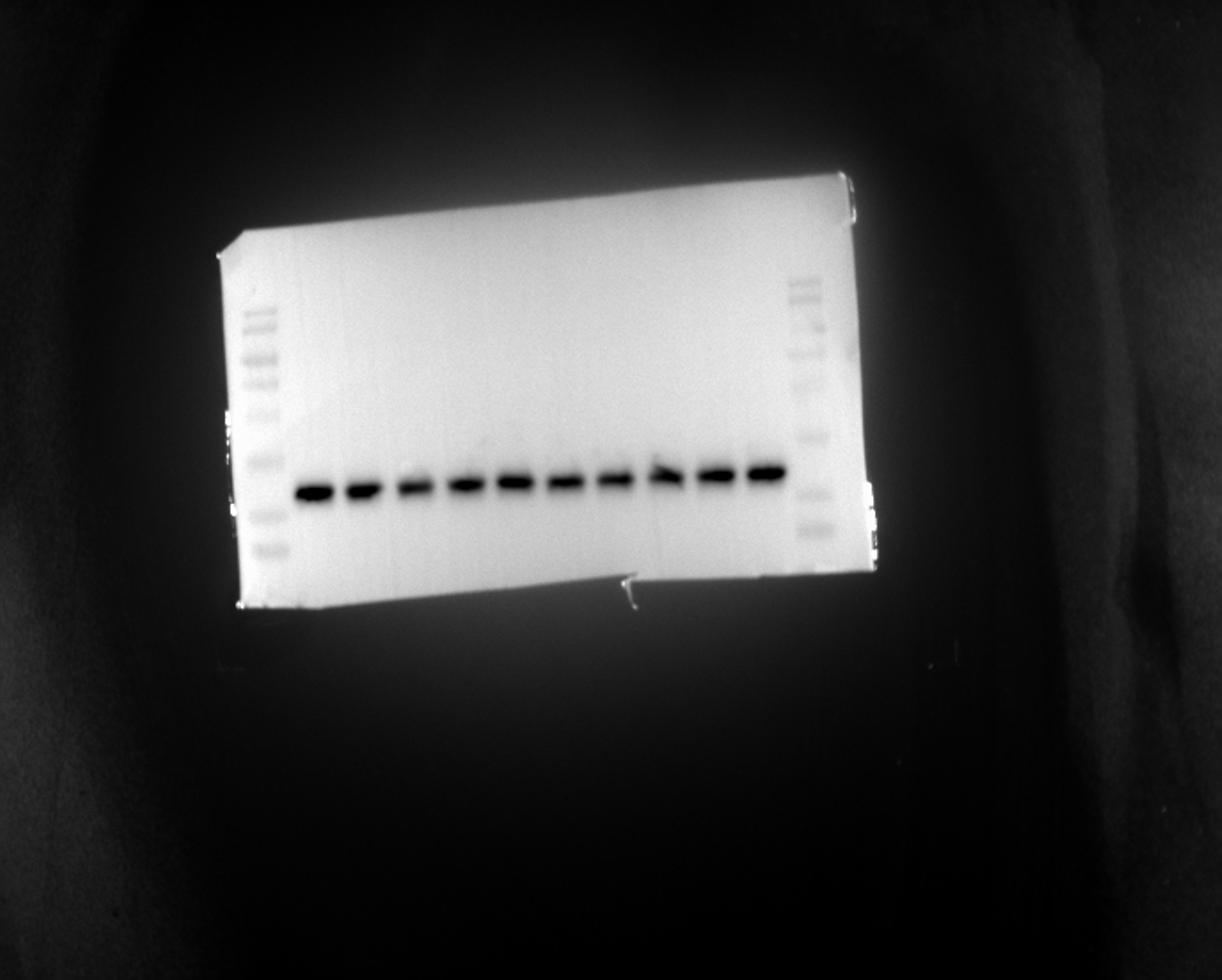


p-P70s6K
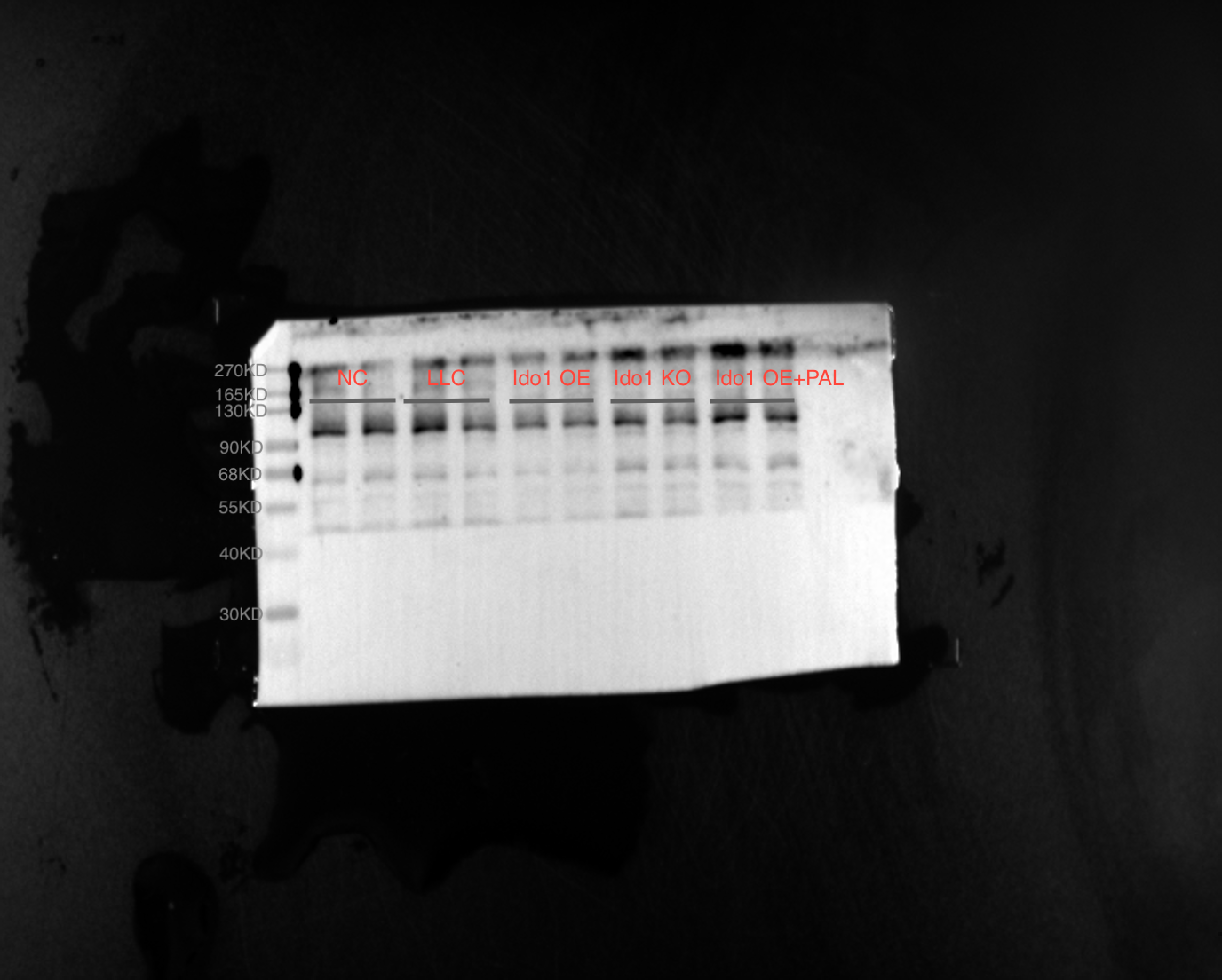


GAPDH
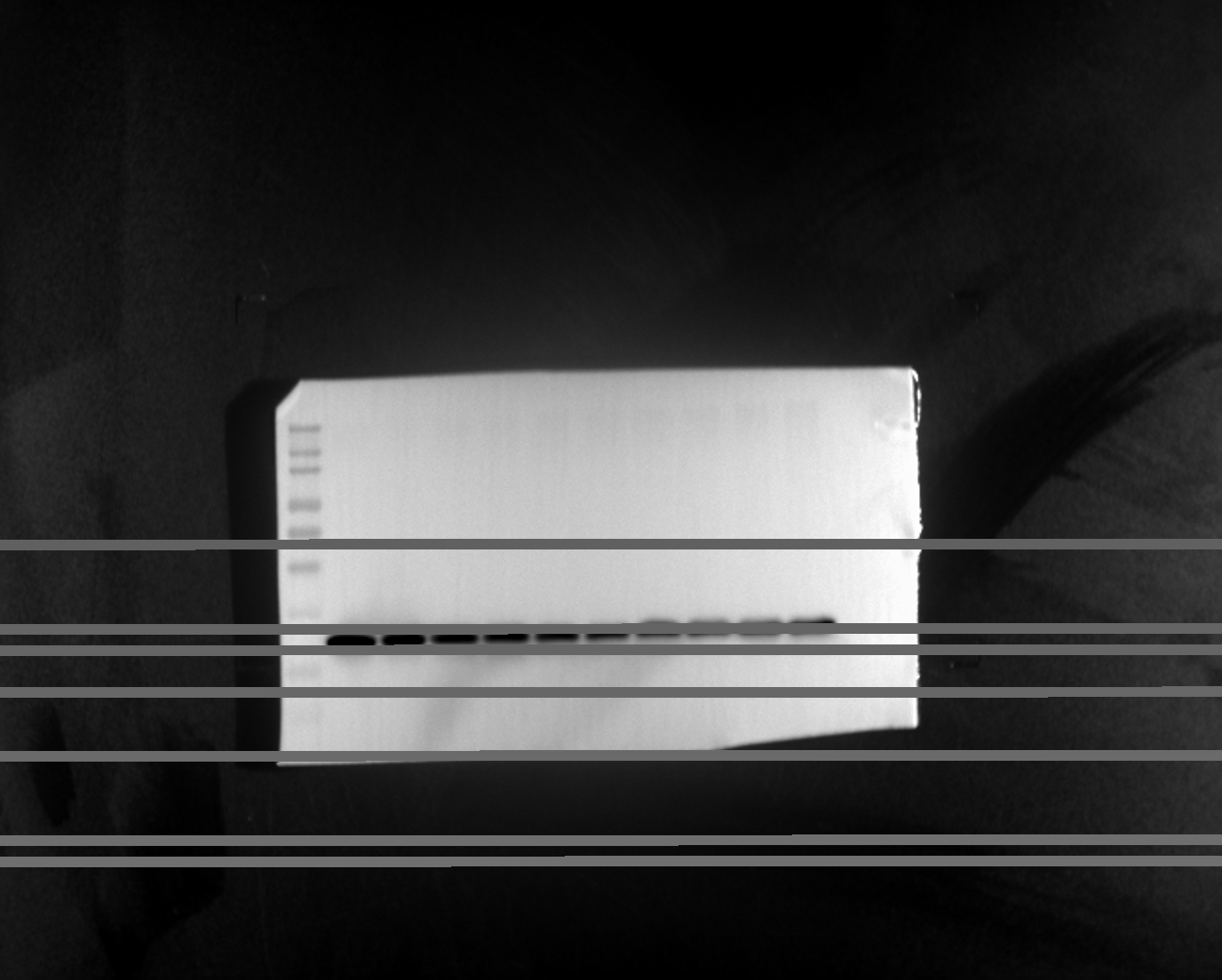

Supplement: Supplementary file 10 — Data S2: Supporting Information. [file JCSM-17-e70295-s005.docx]
